# Identification and Sequence Analysis of Metazoan tRNA 3′-End Processing Enzymes tRNase Zs

**DOI:** 10.1371/journal.pone.0044264

**Published:** 2012-09-04

**Authors:** Zhikang Wang, Jia Zheng, Xiaojie Zhang, Jingjing Peng, Jinyu Liu, Ying Huang

**Affiliations:** Jiangsu Key Laboratory for Microbes and Genomics, School of Life Sciences, Nanjing Normal University, Nanjing, China; University of Lausanne, Switzerland

## Abstract

tRNase Z is the endonuclease responsible for removing the 3'-trailer sequences from precursor tRNAs, a prerequisite for the addition of the CCA sequence. It occurs in the short (tRNase Z^S^) and long (tRNase Z^L^) forms. Here we report the identification and sequence analysis of candidate tRNase Zs from 81 metazoan species. We found that the vast majority of deuterostomes, lophotrochozoans and lower metazoans have one tRNase Z^S^ and one tRNase Z^L^ genes, whereas ecdysozoans possess only a single tRNase Z^L^ gene. Sequence analysis revealed that in metazoans, a single nuclear tRNase Z^L^ gene is likely to encode both the nuclear and mitochondrial forms of tRNA 3′-end processing enzyme through mechanisms that include alternative translation initiation from two in-frame start codons and alternative splicing. Sequence conservation analysis revealed a variant PxKxRN motif, PxPxRG, which is located in the N-terminal region of tRNase Z^S^s. We also identified a previously unappreciated motif, AxDx, present in the C-terminal region of both tRNase Z^S^s and tRNase Z^L^s. The AxDx motif consisting mainly of a very short loop is potentially close enough to form hydrogen bonds with the loop containing the PxKxRN or PxPxRG motif. Through complementation analysis, we demonstrated the likely functional importance of the AxDx motif. In conclusion, our analysis supports the notion that in metazoans a single tRNase Z^L^ has evolved to participate in both nuclear and mitochondrial tRNA 3′-end processing, whereas tRNase Z^S^ may have evolved new functions. Our analysis also unveils new evolutionarily conserved motifs in tRNase Zs, including the C-terminal AxDx motif, which may have functional significance.

## Introduction

tRNA 3′-processing enzyme tRNase Z (also termed RNase Z or 3′-tRNase) is an endonuclease involved in tRNA 3′-end maturation (for reviews see [1], [2], [3], [4]). It cleaves tRNA precursors (pre-tRNAs) after the discriminator nucleotide (the first nucleotide after the acceptor stem) to generate the tRNA 3′-end suitable for CCA addition, which is essential for tRNA aminoacylation.

There are two forms of tRNase Z encoded by distinct genes. The short forms (tRNase Z^S^) and long forms (tRNase Z^L^) vary in length from 279–554 aa and 648–997 aa, respectively. Bioinformatics analyses suggest that tRNase Z^L^ may evolve through tandem gene duplication of tRNase Z^S^ followed by sequence divergence [5], [6], [7].

The species distribution of tRNase Z is complex. tRNase Z^S^ is observed in all archaea sequenced to date and in many bacteria [3], [5], [7]. Prokaryotes only possess tRNase Z^S^ [3]. Usually there is one tRNase Z^S^ gene per genome. In contrast, eukaryotes can have both forms of tRNase Z. In fungi, tRNase Z^L^ is universally distributed, whereas tRNase Z^S^ exists only in certain taxonomic groups [7]. The vast majority of fungi examined have one gene for tRNase Z^L^, which appears to be targeted to both the nucleus and mitochondria [7]. Unlike fungi, plants are represented by multiple genes for tRNase Zs including two tRNase Z^S^s and one to two tRNase Z^L^s [5]. These plant proteins seem to have different subcellular localization. In *A. thaliana*, the two tRNase Z^S^s are localized in the cytosol and chloroplast, respectively, whereas the two tRNase Z^L^s are targeted to both the nucleus and mitochondria and to the mitochondria, respectively [8].

tRNase Z has been reported in a few metazoan species. Humans contain one tRNase Z^S^ (also called ELAC1) and one tRNase Z^L^ (also called ELAC2). Human tRNase Z^S^ is found primarily in the cytosol [9], [10], [11], whereas human tRNase Z^L^ is localized in both the nucleus and mitochondria [9], [10]. Unlike humans, the fruit fly *Drosophila melanogaster* and nematode *Caenorhabditis elegans* contain just one tRNase Z^L^. However, the phylogenetic diversity of tRNase Z in the metazoan kingdom has yet to be comprehensively studied.

tRNase Z belongs to the superfamily of metallo-*β*-lactamase (MBL) which is a large group of proteins with diverse biological functions including antibiotic degradation, stress response, DNA repair and RNA maturation [6], [12], [13], [14], [15]. The MBL superfamily members have very low sequence homology but share five conserved sequence motifs termed Motifs I-V, among which the most characteristic is Motif II (HxHxDH, also called the His motif). These motifs contain invariant histidine and/or aspartic acid residues essential for zinc binding and catalysis. Besides these catalytic motifs, both tRNase Z^S^ and tRNase Z^L^ contain the GP, PxKxRN, HEAT and HST motifs, which adopt loop structures [5]. The GP and PxKxRN motifs may play a role in cleavage specificity [16], [17], whereas the conserved Glu-His pair from the HEAT and HST motifs may facilitate proton transfer in catalysis [18], [19], [20].

The three-dimensional structures of bacterial tRNase Z^S^s from *Bacillus subtilis*, *Escherichia coli* and *T. maritima* [18], [21], [22], [23], [24] and the only crystal structure of eukaryotic tRNase Z^S^ from humans (http://www.rcsb.org/pdb/explore.do?structureId=3ZWF) available so far reveal that the enzymes form a dimer. Each monomer adopts a typical MBL fold, which comprises external *α-*helices and internal *β*-sheets forming a four-layered *αβ*/*αβ* sandwich. The zinc ligands at the active sites of these enzymes are located at loops and turns that connect the *β*-sheets, and are composed of conserved residues mainly from Motifs I-V. More importantly, the crystal structures reveal the tRNase Z-specific domain termed flexible arm (also called exosite) consisting of a compact globular domain and an extended two-stranded stalk. This flexible arm is located on the opposite side of the active site and protrudes from the protein core, and is involved in the substrate binding primarily through binding the D and T loops of the pre-tRNA [24], [25], [26]. However, no high-resolution structures have been solved for any tRNase Z^L^.

We have recently undertaken comprehensive surveys of tRNase Zs in fungi [7] and plants [5]. Here, we extend the analysis to metazoans. Our results provide insights into the origin and evolution of metazoan tRNase Zs and should facilitate further molecular evolutionary and structure-function relationship analyses of tRNase Z.

## Results

### Identification of Candidate Metazoan tRNase Z Genes

As part of our effort to explore genomic distribution and sequence conservation of metazoan tRNase Zs, we performed BLAST similarity searches of metazoan genomic and EST databases. We obtained a total of 45 candidate tRNase Z^S^ genes and 71 tRNase Z^L^ genes from 82 species, including 77 bilaterians, 4 nonbilaterian metazoans, and 1 choanoflagellate (Table S1). It should be noted that tRNase Z^L^ was identified in all metazoan species examined, although the protein sequences of some tRNase Z^L^s could not be accurately predicted due to gaps in their nucleotide sequences. Although the number of basal metazoan and protozoan species that have had their complete genome sequences reported is very limited, the inclusion of these species in our analysis would enable us to have a better understanding of the origin and evolution of metazoan tRNase Zs. Among these candidate metazoan tRNase Z genes identified, only those from *D. melanogaster* [27], [28], *C. elegans* [29] and humans have been experimentally characterized. To present the data in a concise manner, we have selected a list of tRNase Zs of representative species spanning a diverse range of taxonomic groups (Table 1).

**Table 1:** Distribution of candidate tRNase Zs from representative metazoans.

Species	Protein name	Form	Accession number	Database	No. aa#	EST
Deuterostomes						
* Branchiostoma floridae*	BflTRZ1	tRNase Z^S^	XP_002598072.1	NCBI	367*	+
* Branchiostoma floridae*	BflTRZ2	tRNase Z^L^	XP_002603583.1	NCBI	785*	
* Canis familiaris*	CfaTRZ1	tRNase Z^S^	XP_849493.1	NCBI	374	+
* Canis familiaris*	CfaTRZ2	tRNase Z^L^	XP_546630.2	NCBI	821	+
* Ciona intestinalis*	CinTRZ1	tRNase Z^S^	299192	Metazome	383	+
* Ciona intestinalis*	CinTRZ2	tRNase Z^L^	295609	Metazome	727	+
* Danio rerio*	DreTRZ1	tRNase Z^S^	NP_001003503.1	NCBI	372	+
* Danio rerio*	DreTRZ2	tRNase Z^L^	XP_003198113.1	NCBI	895	+
* Homo sapiens*	HsaTRZ1	tRNase Z^S^	NP_061166.1	NCBI	363	+
* Homo sapiens*	HsaTRZ2	tRNase Z^L^	NP_060597.4	NCBI	826	+
* Loxodonta africana*	LafTRZ1	tRNase Z^S^	ENSLAFP00000001283	Ensembl	364	
* Loxodonta africana*	LafTRZ2	tRNase Z^L^	ENSLAFP00000025345	Ensembl	819	
* Mus musculus*	MmuTRZ1	tRNase Z^S^	NP_444485.2	NCBI	362	+
* Mus musculus*	MmuTRZ2	tRNase Z^L^	CAI24609	NCBI	824	+
* Salmo salar*	SsaTRZ1	tRNase Z^S^		NCBI	370	+
* Salmo salar*	SsaTRZ2	tRNase Z^L^	C0H8T6	Uniprot	875	+
* Strongylocentrotus purpuratus*	SpuTRZ1	tRNase Z^S^	XP_001189573.1	NCBI	362	
* Strongylocentrotus purpuratus*	SpuTRZ2	tRNase Z^L^	XP_783904.2	NCBI	931*	+
* Xenopus tropicalis*	XtrTRZ1	tRNase Z^S^	476074	Metazome	363	+
* Xenopus tropicalis*	XtrTRZ2	tRNase Z^L^	157533	Metazome	835*	+
Protostomes						
* Bombyx mori*	BmoTRZ1	tRNase Z^L^	Bmb008309	Metazome	889*	+
* Caenorhabditis elegans*	CelTRZ1	tRNase Z^L^	NP_001023109.1	NCBI	833	+
* Capitella teleta*	CteTRZ1	tRNase Z^L^	228344	JGI	758	+
* Capitella teleta*	CteTRZ2	tRNase Z^L^-Like	218896	JGI	738	+
* Drosophila melanogaster*	DmeTRZ1	tRNase Z^L^	NP_724916.1	NCBI	766	+
* Lottia gigantea*	LgiTRZ1	tRNase Z^S^	218281	JGI	356	+
* Lottia gigantea*	LgiTRZ2	tRNase Z^L^	156489	JGI	738*	+
* Lottia gigantea*	LgiTRZ3	tRNase Z^L^	230203	JGI	780	+
* Tribolium castaneum*	TcaTRZ1	tRNase Z^L^	XP_968692.2	NCBI	827	+
Basal Metazoans						
* Amphimedon queenslandica*	AquTZ1	tRNase Z^S^	Aqu1.217211	JGI	373*	
* Amphimedon queenslandica*	AquTZ2	tRNase Z^L^	XP_003385508.1	NCBI	712*	
* Hydra magnipapillata*	HmaTRZ1	tRNase Z^S^	Hma2.228140	JGI	359	+
* Hydra magnipapillata*	HmaTRZ2	tRNase Z^L^	Hma2.206546	JGI	723*	
* Nematostella vectensis*	NveTRZ1	tRNase Z^S^	186754	JGI	342	+
* Nematostella vectensis*	NveTRZ2	tRNase Z^L^	11980	JGI	827*	
* Nematostella vectensis*	NveTRZ3	tRNase Z^L^	108544	JGI	769*	+
* Trichoplax adhaerens*	TadTRZ1	tRNase Z^L^	B3RMG1	Uniprot	728*	
Protozoans						
* Monosiga brevicollis*	MbrTRZ1	tRNase Z^S^	25108	JGI	380*	
* Monosiga brevicollis*	MbrTRZ2	tRNase Z^L^	23750	JGI	812*	

# The number of amino acids in metazoan tRNase Z proteins.

* Indicates that mispredicted sequences obtained from the databases have been corrected.

A plus sign indicates that gene expression is confirmed by the presence of ESTs.

It appears that the tRNase Z gene is variably distributed within the three major divisions of Bilateria (the Deuterostomia, the Ecdysozoa, and the Lophotrochozoa). All of the vertebrate deuterostomes examined to date have one tRNase Z^S^ and one tRNase Z^L^ genes. Similarly, four invertebrate deuterostomes, the amphioxus *Branchiostoma floridae* (cephalochordate), two sea squirts *Ciona intestinalis* and *Ciona savignyi* (urochordate), and the sea urchins *Strongylocentrotus purpuratus* (echinoderm) have one tRNase Z^S^ and one tRNase Z^L^ genes.

Unlike deuterostomes, protostomes contain variable numbers of tRNase Zs. Ecdysozoans, one of two major groups of protostomes and including animals such as arthropods and nematodes, appear to possess a single tRNase Z^L^ gene but lack the tRNase Z^S^ gene. Lophotrochozoa, which is another major group of protostomes and comprises annelids and molluscs, is significantly underrepresented in terms of whole genome data. Among sequenced lophotrochozoans, the marine mollusk *Lottia gigantea* appears to have the largest number of tRNase Zs: one tRNase Z^S^ and two tRNase Z^L^. Like deuterostomes, the marine mollusk *Aplysia californica* and the two parasitic flatworms (*Schistosoma mansoni* and *Schistosoma japonicum*) harbor one tRNase Z^S^ and one tRNase Z^L^ genes. Like ecdysozoans, the two marine annelids *Helobdella robusta* and *Capitella teleta* appear to have one tRNase Z^L^ gene, but have no tRNase Z^S^ gene. In addition, *C. teleta* has one tRNase Z^L^-like protein, which lacks the GP-motif.

The number of tRNase Z genes in basal metazoans examined so far seems highly variable. The freshwater hydra *Hydra magnipapillata*, a representative cnidarian, and the marine demosponge *Amphimedon queenslandica* (phylum Porifera) each contain one tRNase Z^S^ and one tRNase Z^L^ genes. In the starlet sea anemone *Nematostella vectensis*, another representative cnidarian, one tRNase Z^S^ and two tRNase Z^L^ genes were identified. *Trichoplax adhaerens*, the sole representative of the phylum Placozoa, represents the simplest known animal with the smallest known animal genome. In this species, only one tRNase Z^L^ gene was found, but no tRNase Z^S^ genes were identified. In the choanoflagellate *Monosiga brevicollis*, which is the closest known unicellular relative to metazoans, two tRNase Z genes (one tRNase Z^S^ gene and one tRNase Z^L^ gene) were identified.

### Prediction of the Number of Introns in Metazoan tRNase Z^S^ and tRNase Z^L^ Genes

To understand the structure of metazoan tRNase Z genes, we predicted their introns. Metazoan tRNase Z^S^ genes appear to have few introns. For example, all mammalian tRNase Z^S^ genes examined have just two introns (Table S2). Mammals and inverterbrate tRNase Z^L^s seem to have different numbers of introns. All mammalian tRNase Z^L^ genes examined contain 23 introns. In contrast, tRNase Z^L^ genes of 3 *Caenorhabditis* species including *C. elegant* and 9 *Drosophila* species including *D. melanogaster* contain 6 and 1 introns, respectively. The difference in intron numbers between mammalian and invertebrate tRNase Z^L^ genes can be ascribed to massive, lineage-specific intron loss in worms and insects [30]. Interestingly, the intron found in *Drosophila* tRNase Z^L^ genes splits the gene into two fragments that encode roughly the N- and C-terminal halves of the protein. This is consistent with the notion that proteins are often made from independently-folded functional domains encoded by separate exons, which can be separated by introns [31], [32]. Unexpectedly, we found that the ascidian *C. intestinalis* tRNase Z^L^ gene encoding a protein of 727-aa apparently does not have any intron by comparing the gene sequence with corresponding EST sequences in NCBI databases.

### Alternative Splicing of tRNase Z^L^ Transcripts

**Figure 1:**
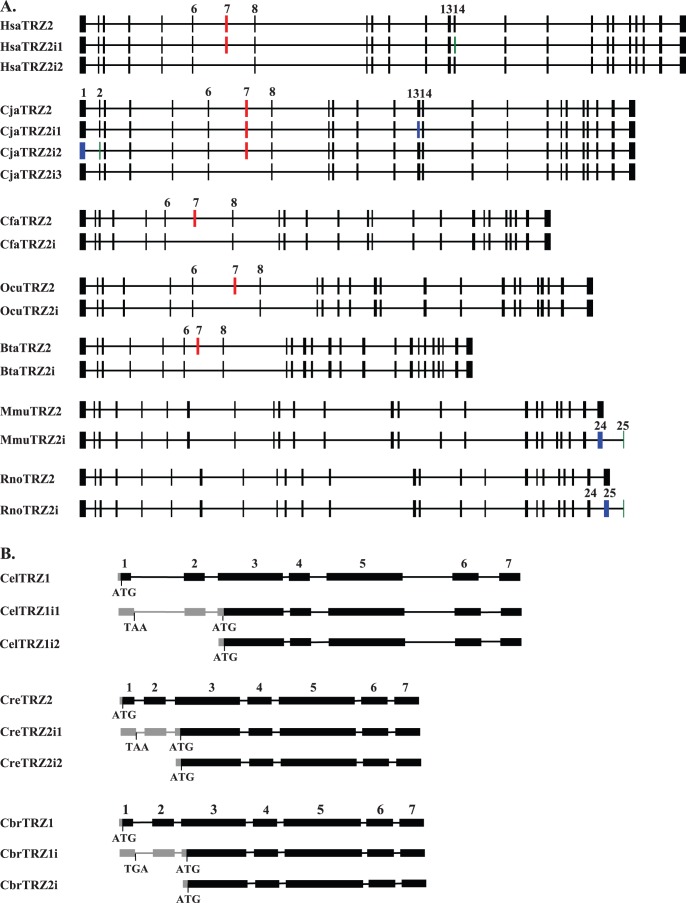
Schematic representation of the predicted alternative splice variants of mammalian and nematode tRNase Z^L^ genes (drawn to scale). (A) Mammalian tRNase Z^L^s are from *H. sapiens* (Hsa), *C. jacchus* (Cja), *C. familiaris* (Cfa), *O. cuniculus* (Ocu), *B. taurus* (Bta), *M. musculus* (Mmu) and *R. norvegicus* (Rno). HsaTRZ2i1, HsaTRZ2i2, CjaTRZ2i1, CjaTRZ2i2, CjaTRZ2i3, CfaTRZ2i, OcuTRZ2i, BtaTRZ2i, MmuTRZ2i, and RnoTRZ2i correspond to alternatively spliced variants. Constitutive exons and introns are represented by the filled boxes and intervening horizontal lines respectively. Alternative exons are colored as follows: exon skipping in red, exon with alternative donors in blue and exon with alternative acceptors in green. The locations of the alternative exons in the tRNase Z^L^ genes and its splice variants are shown on the top of the exon. (B) Nematode tRNase Zs are from *C. elegans* (Cel), *C. remanei* (Cre) and *C. briggsae* (Cbr). CelTRZ1i1, CelTRZ1i2, CreTRZ1i1, CreTRZ1i2, CbrTRZ1i1 and CbrTRZ1i2 are splice variants. Exons are shaded black, introns are indicated by lines and the 5′-untranslated region (5′-UTR) sequences are indicated by gray boxes. The exon number indicated on top. The ATG start codon and TAA stop codon are indicated.

Alternative splicing can play a major role in modulating gene structure and function. To identify potential alternative spliced isoforms of tRNase Z^L^, we performed BLAST searches against the NCBI EST database. Candidate splice variants of tRNase Z^L^ were largely found in vertebrates, consistent with the observation that vertebrates have substantially higher rates of alternative splicing compared to other species. Figure 1 shows a schematic representation of predicted alternatively spliced variants of metazoan tRNase Z^L^. The common marmoset (*Callithrix jacchus*) appears to have three alternatively spliced isoforms of tRNase Z^L^, designated CjaTRZ2i1, CjaTRZ2i2 and CjaTRZ2i3. CjaTRZ2i1 (mRNAs) is created by using an alternative splice donor within exon 13. The use of this alternative acceptor site (within tRNase Z^L^ exon) causes a deletion of V703. CjaTRZ2i2 uses an alternative donor site within exon 1 and an acceptor site within exon 2 that results in a deletion of 57 nt. CjaTRZ2i3 is generated by skipping of exon 7 that causes an internal 120-nt deletion.

Alternative splicing of tRNase Z^L^ may also occur in humans and may result in the expression of two splicing variants, designated HsaTRZ2i1 and HsaTRZ2i2. Whereas HsaTRZ2i1 is produced by using an alternative in-frame acceptor site within exon 14 that leads to loss of one amino acid (aa) residue Lys407 compared to the human tRNase Z^L^, HsaTRZ2i2 is generated as a result of exon 7 skipping that deletes 120 nt. Evidence of skipping of exon 7 is provided by annotated GenBank cDNAs isolated from the cDNA libraries of human brain, spleen and lung (GenBank accession numbers: DA954223.1, DA178234.1 and AW206103.1).

Alternative splicing appears to generate two tRNase Z^L^ isoforms in the domestic dog (*Canis familiaris*) and European rabbit (*Oryctolagus cuniculus*). Similar to CjaTRZ2i3 and HsaTRZ2i1, both these two alternatively spliced tRNase Z^L^s are produced by skipping exon 7.

The mouse tRNase Z^L^ has two alternative spliced transcripts (MmuTrz2 and MmuTrz2i), which has similar organization of the exons and introns except for the alternatively spliced form (MmuTrz2i), which contains a truncated (24th) exon 24 and an additional new exon of 33 bp (exon 25) at the end of the gene (Figure 1). Similar alternative splicing occurs in the rat tRNase Z^L^. As a result, the major mouse and rat tRNase Z^L^ transcripts contain 24 exons encoding proteins of 824 and 827 aa respectively, whereas their alternative spliced transcripts contain 25 exons and encode proteins of 831 and 834 aa respectively. The amino acid sequences encoded by the additional exon 25 are poorly conserved between rodents (mouse and rat). The generation of this alternate splice form appears to occur only in rodents, but not in humans, and both splice forms are present in most mouse and rat tissues analyzed [33] (see Discussion).

In addition to vertebrates, splice variants appear to be present in three *Caenorhabditis* nematodes (*C. elegans*, *Caenorhabditis briggsae* and *Caenorhabditis remanei*). In *C. elegans*, three different splice transcripts of tRNase Z^L^ (CelTrz1, CelTrz2i1 and CelTrz2i2 corresponding to E04A4.A, E04A4.4B and E04A4.4C described in [29], respectively) were detected by reverse transcriptase (RT)-PCR [29]. CelTrz1, which can be trans-spliced to one of two short leader RNAs, SL1 or SL2, encodes the full length protein containing a putative mitochondrial targeting sequence (MTS). CelTrz2i1 is trans-spliced to either SL1 or SL2. Although CelTrz2i1 begins with the first exon, translation begins from a start codon within the third exon since it contains an in-frame stop codon (TAA) in the 5′-end of its pre-mRNAs (Figure 1). CelTrz2i2 is trans-spliced to SL1 and begins at the start of exon 2. Like CelTrz2i1, the translation of CelTrz2i2 starts from an ATG present in exon 3. Both CelTrz2i1 and CelTrz2i2 transcripts apparently encode the same tRNase Z^L^ isoform that lacks an N-terminal MTS and is likely targeted to the nucleus. Comparison of the tRNase Z^L^ gene structures of three *Caenorhabditis* nematodes suggested that similar mechanisms to generate splice variants a single tRNase Z^L^ gene may operate in other two nematodes (Figure 1).

### Phylogenetic Analysis

**Figure 2:**
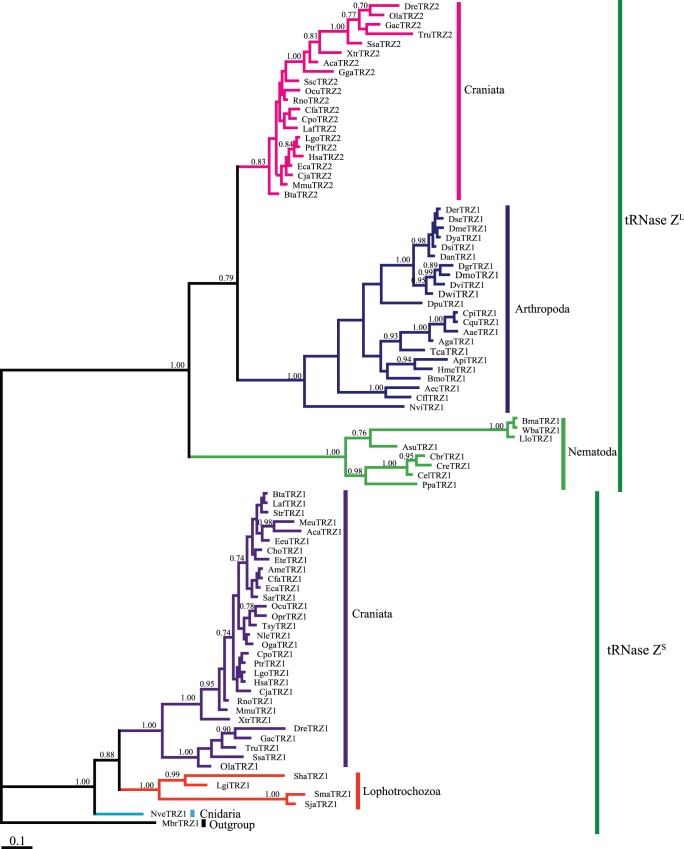
Bayesian phylogenetic tree of predicted metazoan tRNase Zs. This analysis is based on the sequence alignment of the full-length metazoan tRNase Z^S^s with the C-terminal half of metazoan tRNase Z^L^s. The accession number for each candidate tRNase Z is identified in Table S1. Bayesian posterior probabilities are indicated at the nodes. The scale bar indicates 0.1 nucleotide substitutions per site. Taxonomic designations are indicated on the right side of the tree.

To evaluate the evolutionary relationship among the metazoan tRNase Zs, we performed a Bayesian phylogenetic analysis using a data set composed of 86 representative tRNase Zs from taxonomically diverse metazoans. In addition to the metazoan species, the premetazoan choanoflagellate *M. brevicollis* tRNase Z^S^ was included as an outgroup. Our phylogenetic analysis showed that candidate sequences cluster into two well-supported sister clades (Figure 2). One clade, termed the tRNase Z^L^ clade, comprises all tRNase Z^L^s, and the other one, termed the tRNase Z^S^ clade, includes all tRNase Z^S^s. This phylogenetic tree suggests that the metazoan tRNase Z^S^s and tRNase Z^L^s share a common ancestor. Moreover, the phylogenetic relationships of tRNase Zs within each of the clades generally follow the species phylogeny [34] except for nematode tRNase Z^L^s (Figure 2).

### Prediction of Subcellular Localization of Metazoan tRNase Zs

To understand the functions of metazoan tRNase Zs, we predicted subcellar localization of candidate metazoan tRNase Zs. All metazoan tRNase Z^S^s examined do not contain any predictable subcellular localization signal and are expected to be cytoplasmic proteins. In contrast, the vast majority of analyzed candidate tRNase Z^L^s contain both an N-terminal MTS and a nuclear localization signal (NLS), and are predicted to be targeted to both the nucleus and mitochondria (Table S3). Indeed, subcellular localization studies using fluorescence microscopy have shown that human tRNase Z^L^ localizes to both the nucleus and mitochondria, whereas human tRNase Z^S^ exists in the cytosol [9], [35], [36]. Also consistent with the prediction of subcellular localization, the *Drosophila* tRNase Z^L^ has been shown to participate in both nuclear and mitochondrial tRNA 3′-end processing [28].

### Analysis of Metazoan tRNase Z Protein Sequences

**Figure 3:**
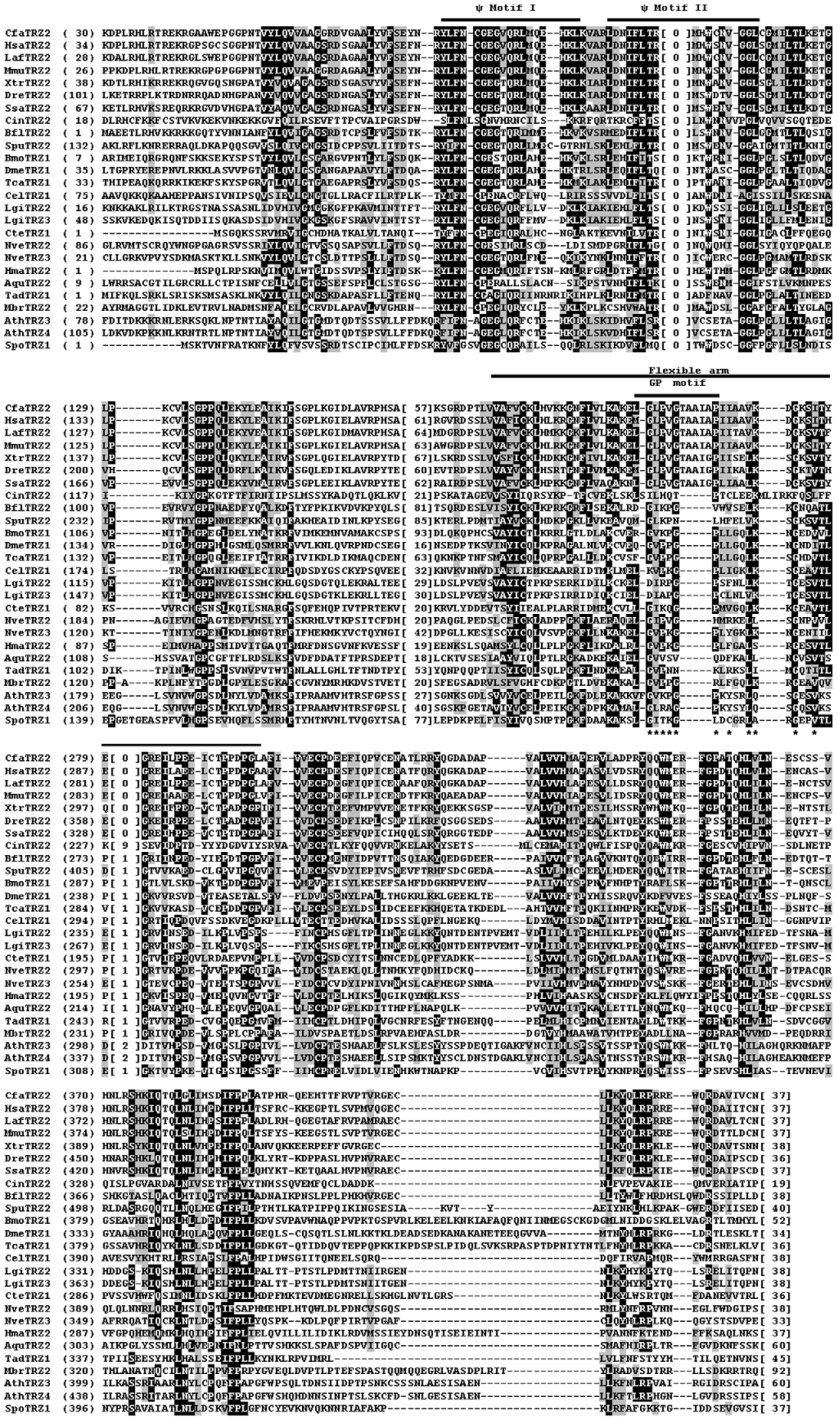
Multiple sequence alignment of representatives of N-terminal halves of the metazoan and non-metazoan tRNase Z^L^s. Representative metazoan tRNase Z^L^s are from ten deuteromes including *C. familiaris* (Cfa), *H. sapiens* (Hsa), *L. Africana* (Laf), *M. musculus* (Mmu), *X. tropicalis* (Xtr), *D. rerio* (Dre), *S. salar* (Ssa), *C. intestinalis* (Cin), *B. floridae* (Bfl), and *S. purpuratus* (Spu), six protostomes including *B. mori* (Bmo), *D. melanogaster* (Dme), *T. castaneum* (Tca), *C. elegans* (Cel), *L. gigantean* (Lgi), and *C. teleta* (Cte), and four basal metazoans including *N. vectensis* (Nve), *H. magnipapillata* (Hma), *A. queenslandica* (Aqu), and *T. adhaerens* (Tad). Non-metazoan tRNase Z^L^s are from the unicellular choanoflagellate *M. brevicollis* (Mbr), green plant *A. thaliana* (Ath) and fungal *S. pombe* (Spo). See Table 1 for more information. The alignment was constructed using the computer program Clustal W [52]. Identical residues are on a black ground and conserved residues shaded in gray. The conserved motifs of tRNase Zs indicated above the alignment are labeled according to references [17], [19], [58]. The numbers in brackets indicate the length of the region in the protein, which are species-specific and could not be correctly aligned. Hyphens represent gaps introduced into sequences for maximum alignment. Amino acid residues predicted to be critical for activity are indicated by a star.

**Figure 4:**
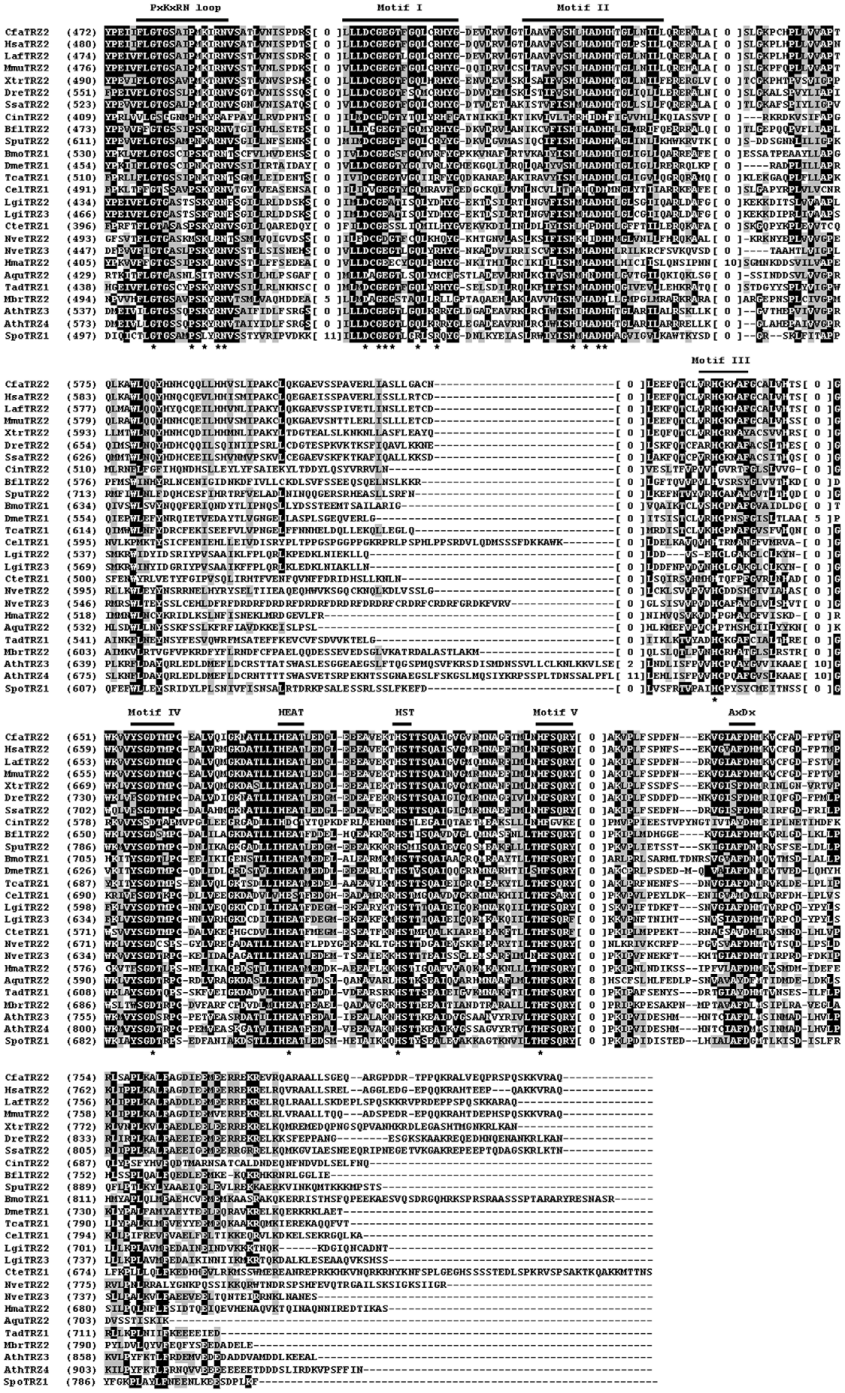
Multiple sequence alignment of representatives of C-terminal halves of the metazoan tRNase Z^L^s. Same legend as in Figure 3.

To assess the sequence conservation and structural features of metazoan tRNase Zs, we performed multiple sequence alignments of candidate sequences. We analyzed candidate tRNase Z^L^s first since they are most likely candidate endonucleases for tRNA 3′-end processing. Since tRNase Z^L^ can be separated into the N- and C-terminal halves with weak sequence similarity to each other, we aligned these two halves separately. Figures 3 and 4 show the alignments of the N- and C-terminal halves of tRNase Z^L^s from representative metazoan species (see Figure S1 for a complete list). Amino acid sequence comparison shows that the coding sequences of the vertebrate tRNase Z^L^s are highly conserved. For example, the human tRNase Z^L^ exhibits an overall sequence identity of 79–82% to dog (*C. familiaris*), bovine (*S. scrofa*), rabbit (*O*. *cuniculus*), mouse (*M. musculus*), Xenopus (*X. tropicalis*) and zebrafish (*D. rerio*) tRNase Z^L^s (summarized in Table S4).

In contrast, the primary sequences of vertebrate and invertebrate tRNase Z^L^s are highly divergent. As summarized in Table S4, the sequence identities between the human and invertebrate tRNase Z^L^s are below ∼32%. Notably, human tRNase Z^L^ shares 19% and 20% sequence identity, respectively, with its orthologs from two ascidian species *C. intestinalis* and *C. savignyi* (marine invertebrates of the chordate subphylum *Urochordata*), suggesting a very distant phylogenetic relationship.

Sequence conservation between the metazoan and plant, yeast or protozoan tRNase Z^L^s is low (∼24% identity between human tRNase Z^L^ and either plant or yeast, tRNase Z^L^, ∼22% between human and protozoan tRNase Z^L^s). Moreover, the conserved amino acid sequences in these tRNase Z^L^s are primarily located at the sequence motifs common to all eukaryotic tRNase Z^L^s, suggesting that the overall fold of tRNase Z^L^s is most likely to be evolutionarily conserved across diverse species from unicellular eukaryotes to metazoans.

Interestingly, all 42 candidate tRNase Z^L^s from Craniata (hagfishes, lampreys and vertebrates), the largest subphylum of chordates, contain a distinct GP motif, in which a 5-aa peptide is inserted between conserved residues Gly and Pro in the GP motif. These insertion sequences in tRNase Z^L^s from mammals, birds, reptiles, amphibians and fishes that have been examined are TAAIA, TPAIL, TAAIA, TAAIG and TAAIG, respectively. This 5-aa insertion seems to be unique to craniate tRNase Z^L^s since this insertion is only found in craniate tRNase Z^L^s, but not found in any of other eukaryotic tRNase Z^L^s. It is unknown why craniate tRNase Z^L^s have a diagnostic 5-aa insertion, which does not seem to be needed for enzyme activity since they are not found in many other tRNase Z^L^s.

**Figure 5:**
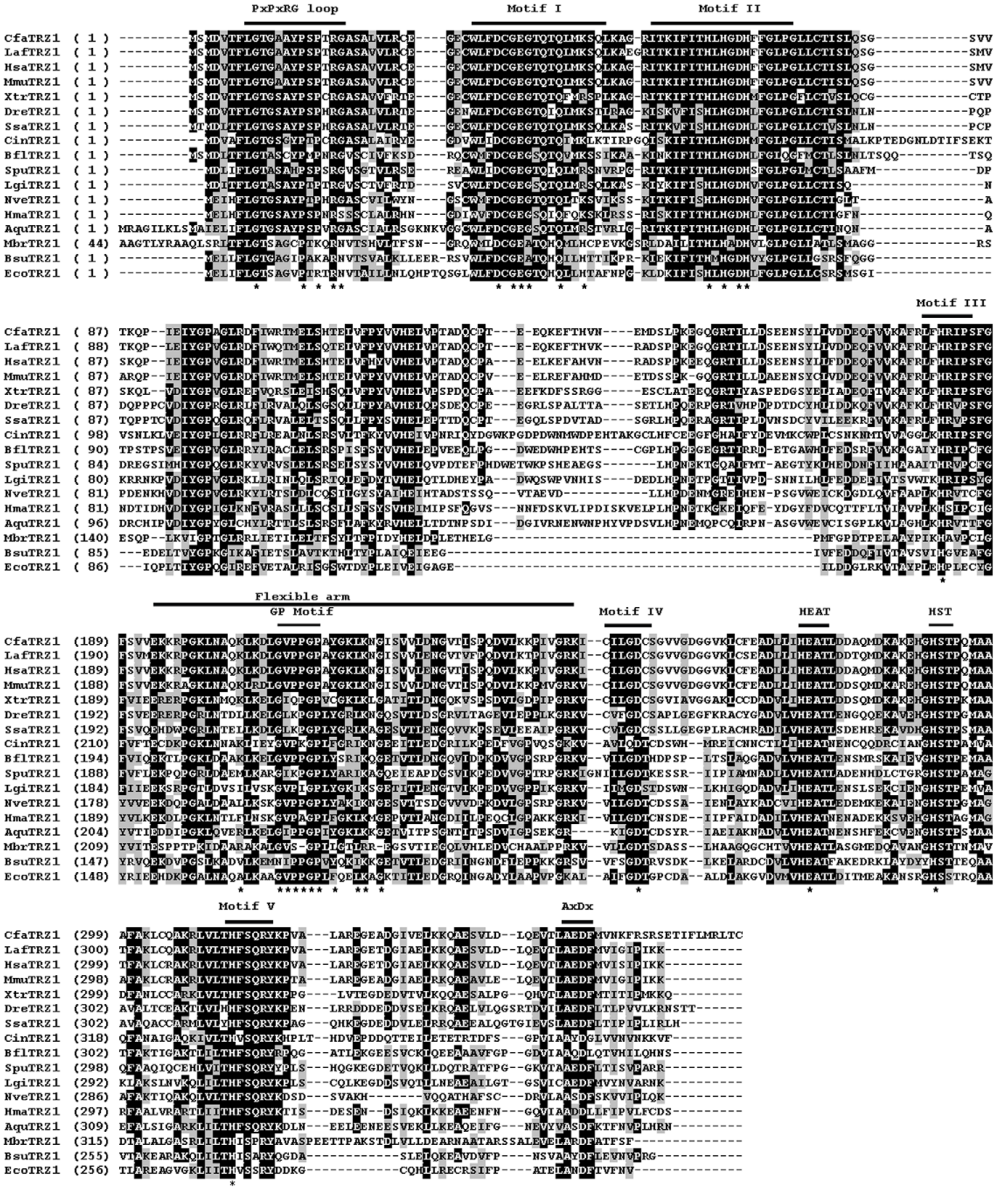
Multiple sequence alignment of representatives of the metazoan and non-metazoan tRNase Z^S^s. Metazoan tRNase Z^S^s are from ten deuteromes including *C. familiaris* (Cfa), *L. africana* (Laf), *H. sapiens* (Hsa), *M. musculus* (Mmu), *X. tropicalis* (Xtr), *D. rerio* (Dre), *S. salar* (Ssa), *C. intestinalis* (Cin), *B. floridae* (Bfl), and *S. purpuratus* (Spu), one protostome, *L. gigantean* (Lgi), and three basal metazoans, *N. vectensis* (Nve), *H. magnipapillata* (Hma), and *A. queenslandica* (Aqu). Non-metazoan tRNase Z^S^s are from the unicellular choanoflagellate *M. brevicollis* (Mbr), *B. subtilis* (Bsu) and *E. coli* (Eco). The annotation of the alignment is as described in the legend to Figure 3.

Figure 5 shows alignments of representative metazoan tRNase Z^S^s (for a complete list, see Figure S2). Sequence alignment revealed that similar to tRNase Z^L^, tRNase Z^S^ is highly conserved throughout vertebrate evolution (see Figure 5 and Table S5). For example, the human tRNase Z^S^ shares approximately 92% and 57% sequence identity with the mouse and zebrafish tRNase Z^S^s, respectively. In contrast, vertebrate tRNase Z^S^s show much lower overall sequence identity to tRNase Z^S^s from protostome invertebrates and basal metazoans. The amino acid sequence identity of the human tRNase Z^S^ with the tRNase Z^S^s from the human parasite *Schistosoma mansoni* and starlet sea anemone *Nematostella vectensis* is 35% and 45%, respectively. The amino acid sequences of vertebrate tRNase Z^S^s are also markedly diverged from those of bacterial tRNase Z^S^s. The sequence of the human tRNase Z^S^ shares 34% and 47% sequence identity to those from *B. subtilis* and *E. coli*, respectively.

**Figure 6:**
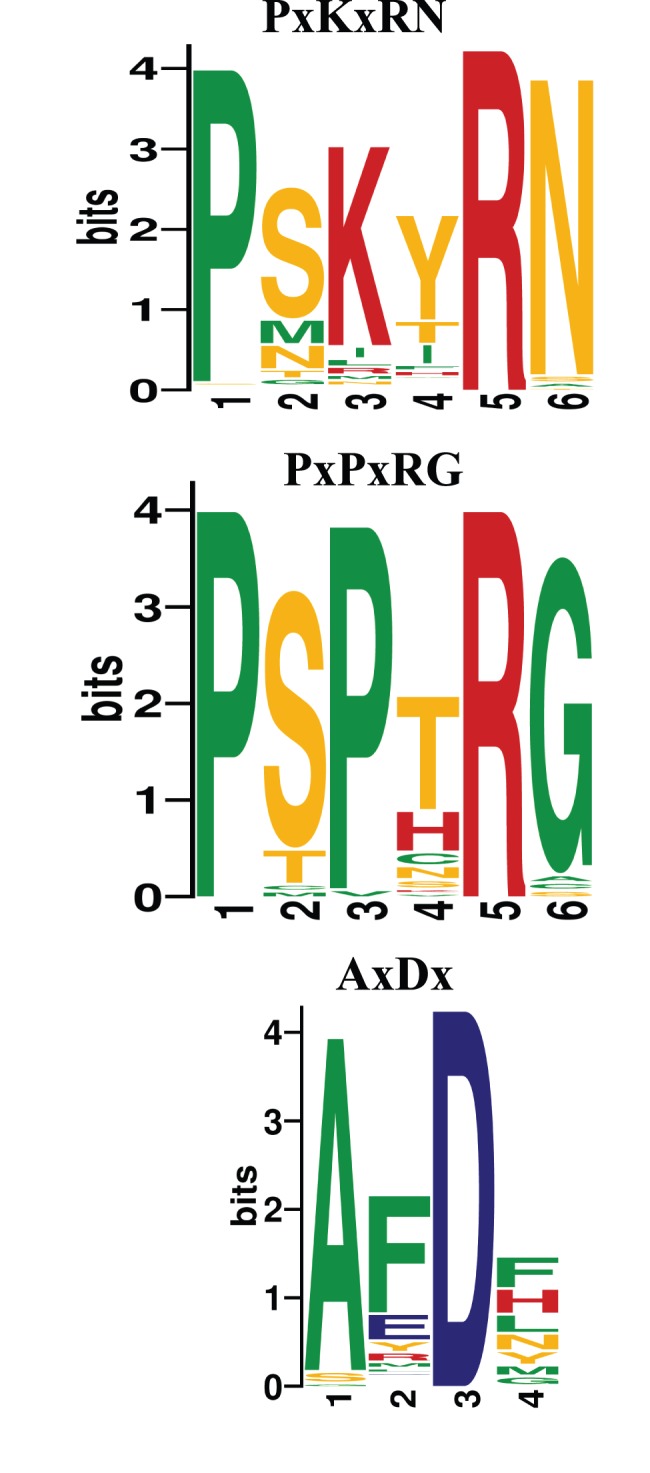
Sequence logos for the predicted PxRxRN, PxPxRG and AxDx motifs. The sequence logo of the PxRxRN motif was derived from 174 tRNase Z^L^s from 75 fungi, 21 plants and 68 metazoans. The sequence log of the PxPxRG motif is derived from alignment of 44 metazoan tRNase Z^S^s. The sequence logo of the AxDx motif is derived from 240 alignments including 178 tRNase Z^L^s from 71 metazoan, 21 green plants, 77 fungi, and 62 tRNase Z^S^s from 30 metazoans, 9 fungi, 2 bacteria and *M. brevicollis*. The sequence logos were created using WebLogo (http://weblogo.berkeley.edu). The height of each amino acid indicates the level of conservation at that position. Amino acids are colored as follows: red, basic; blue, acidic; orange, polar; and green, hydrophobic.

Unlike bacterial tRNase Z^S^s and eukaryotic tRNase Z^L^s, metazoan tRNase Z^S^s appear to contain a variant PxKxRN motif (i.e., the PxPxRG motif) that contains Pro and Gly in place of Lys and Asn respectively (Figure 6). The Lys to Pro substitution is expected to increase the rigidity of the PxKxRN loop, whereas the Asn to Gly substitution may decrease the rigidity of the loop.

Careful examination of the sequences of metazoan tRNase Z^S^s revealed an AxDx motif located near the C-terminal region of the proteins (Figure 5). A reexamination of all previously identified tRNase Z sequences revealed that this motif is also present in the C-terminal regions of tRNase Z^L^s and bacterial-type tRNase Z^S^s but not in *Thermotoga maritima* (TM)-type tRNase Z^S^s [5], [7]. The AxDx motif contains an absolutely conserved Asp residue and a highly conserved Ala residue (Figure 6).

### The Effect of the Point Mutation in the AxDx Motif on the Complementation Ability of the *S. pombe* tRNase Z^L^

**Figure 7:**
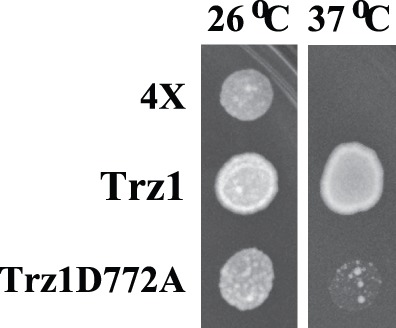
Growth complementation analysis of *S. pombe* Trz1 with a mutation in the AxDx motif. A *S. pombe* strain bearing a temperature-sensitive allele of *trz1* (*trz1-1*) was transformed with empty vector (4X) or vector expressing the wild-type Trz1 or the Trz1D772A mutant, and equal amounts of cells were spotted on EMM medium supplemented with leucine and adenine and grown at 26°C or 37°C.

To establish the functional significance of the AxDx motif, we employed a *Schizosaccharomyces pombe*-based functional complementation assay to assess the functional consequence of the point mutation in the conserved AxDx motif of the *S. pombe* Trz1. As such, we introduced the D772A mutation into the AxDx motif of the *S. pombe* Trz1 and tested if these mutants could rescue a *S. pombe trz1* ^+^ temperature-sensitive (ts) mutant strain *trz1-1* as described before [37]. As shown in Figure 7, the wild type *S. pombe* Trz1 could complement the temperature sensitive growth phenotype of *trz1-1* when expressed from the pREP4X plasmid. On the other hand, the D772A mutant protein showed an impaired ability to complement the temperature-sensitive growth defect of *trz1-1*. Based on these results, we concluded that the highly conserved AxDx motif and particularly Asp772 is vital for the *S. pombe* Trz1 activity and function.

## Discussion

### The Origin and Evolution of tRNase Z in Metazoans

Our survey of available metazoan genomes revealed that the tRNase Z^L^ gene is ubiquitous across the metazoan kingdom, including nonbilaterian metazoans. On the other hand, the tRNase Z^S^ gene does not exist in any of the sequenced ecdysozoan genomes, which is the largest clade of metazoans. The presence of the tRNase Z^S^ and tRNase Z^L^ genes in many higher metazoans such as lophotrochozoans and deuterostomes, and in lower metazoans including poriferans and cnidarians indicates that the tRNase Z^S^ and tRNase Z^L^ genes were already present very early in the evolution of metazoans, and that the absence of tRNase Z^S^ in ecdysozoans is likely due to gene loss. This hypothesis is further reinforced by our finding that both tRNase Z^S^ and tRNase Z^L^ genes already existed in the unicellular choanoflagellate *M. brevicollis*, which is the closest known relative of metazoans. Our findings are consistent with the observations that ecdysozoans have high rates of gene loss [38], [39].

There are two possible explanations for the loss of the tRNase Z^S^ gene in ecdysozoans. First, the function of the tRNase Z^S^ gene might largely represent the vertebrate-specific feature underlying the biological complexity of vertebrates. However, the role of the vertebrate tRNase Z^S^ gene has not been fully addressed (see below discussion). Second, the tRNase Z^S^ gene could be an animal ancestral gene that has been lost during Ecdysozoan evolution.

### The Role of Metazoan tRNase Zs

In our early bioinformatics studies of fungi and plant tRNase Zs, we concluded that tRNase Z^L^ takes over the essential role of tRNase Z^S^ in the nuclear and mitochondrial tRNA 3′-end processing. The present bioinformatics analysis for metazoan tRNase Zs extends and reinforces this conclusion.

The conclusion that tRNase Z^L^ is responsible for the 3′-end processing of nuclear and mitochondrial tRNA precursors in metazoans is further supported by the results of both in vitro and in vivo functional studies. In humans, tRNase Z^L^ exhibits tRNA processing efficiency ∼2000 fold higher than tRNase Z^S^ in vitro [40]. Recent in vivo studies of tRNase Z^L^ from *Drosophila* and humans provide direct evidence that tRNase Z^L^ is responsible for endonucleolytic processing of the 3′-end of pre-tRNAs in metazoan nucleus and mitochondria [28]. The *Drosophila* tRNase Z^L^ is encoded by the juvenile hormone (JH) inducible gene, *JhI-1* [41]. RNA interference (RNAi)-mediated silencing of the *JhI-1* gene expression in *D. melanogaster* results in the accumulation of 3′-end unprocessed tRNAs in both the nucleus and mitochondria [28], indicating that tRNase Z^L^ is responsible for generating the mature 3′-end of tRNAs in the nucleus and mitochondria of *D. melanogaster*. Likewise, in humans, tRNase Z^L^ is the primary enzyme responsible for mitochondrial tRNA 3′-end since silencing of the human tRNase Z^L^ gene inhibits the 3′-end processing of mitochondrial tRNAs [9], [11]. However, among some of the mitochondrial tRNAs analyzed, siRNA against the human tRNase Z^L^ gene only affects the mature level of a few mitochondrial tRNAs in humans [9], [11].

In *Drosophila*, knockdown of the tRNase Z^L^ gene significantly decreases the mature levels of the mitochondrial tRNAs but not the mature levels of all five tested nuclear tRNAs [28]. It is not known why the steady-state levels of many tested mature forms of tRNAs are not visibly affected. One possibility is that additional enzymes may also be involved in 3′-end processing of tRNAs.

Besides its primary role in tRNA maturation, the human tRNase Z^L^ functions in other cellular pathways. For example, the human tRNase Z^L^ has been demonstrated to be involved in the biogenesis of non-coding RNAs (ncRNAs), other than tRNA, such as metastasis associated lung adenocarcinoma transcript 1 (MALAT1) [42], tRNA-derived small RNAs [43], [44] and viral microRNAs (miRNAs) [45], [46]. It would be interesting to see if other metazoan tRNase Z^L^s also have additional functions apart from tRNA 3′-end processing.

So far, the metazoan tRNase Z^S^ has only been studied in humans and its function is still poorly understood. The human tRNase Z^S^ gene appears to be nonessential since this gene was found to be deleted in a lung cancer cell line, Ma29 [47]. The human tRNase Z^S^ is localized in the cytoplasm and has been proposed to play a role in fine-tuning of gene expression by processing and degradation of unstructured RNAs [11]. It will be important to determine the RNA substrates of the metazoan tRNase Z^S^s by various methods including coimmunoprecipitation with an antibody against tRNase Z^S^, followed by sequencing of associated RNAs.

### Mechanisms for Dual Localization of Metazoan tRNase Z^L^s

How does the metazoan tRNase Z^L^ participate in both the mitochondrial and nuclear tRNA 3′-end processing? A recent study on the subcellular localization of tRNase Z^L^ suggests that mammalian tRNase Z^L^ genes encode both nuclear and mitochondrial forms of tRNase Z^L^ by using alternative translation initiation sites [36]. Our results extend this conclusion to include other metazoan tRNase Z^L^s. We found that like mammalian tRNase Z^L^s, most non-mammalian metazoan tRNase Z^L^s also contain a putative downstream AUG start codon in-frame with the first one (Figure S3). In additional, the nucleotide context around the first start codon is apparently suboptimal for translational initiation as defined by the consensus sequences [48], [49]. We also found that most metazoan tRNase Z^L^s analyzed so far are characterized by the presence of the N-terminal MTS. Thus, it is likely that a single metazoan tRNase Z^L^ mRNA is translated via leaky scanning to make proteins with or without an MTS by initiating translation at two in-frame AUG codons that should produce essentially the same proteins, one mitochondrial and the other nuclear. Indeed, changing the nucleotide sequences surrounding the first AUG start codon of the human tRNase Z^L^ to conform to the eukaryotic consensus sequence results in exclusive mitochondrial localization of the protein, whereas removing the N-terminal region of the human tRNase Z^L^ encoding most of the predicted MTS causes the protein to be targeted exclusively to the nucleus [36].

Besides leaky scanning, other mechanisms including alternative splicing [50] may also be used to produce the nuclear and mitochondrial forms of the metazoan tRNase Z^L^ from the same gene. Examination of the tRNase Z^L^ genes from three *Caenorhabditis* nematodes revealed that all of the candidate genes contain a second putative in-frame start codon located in exon 3 (Figure 1). The two AUG start codons are separated by 406 nt (*C. remanei* tRNase Z^L^), 1248 nt (*C. elegans*) and 413 nt. (*C. briggsae*), respectively. These distances seem too long for efficient leaky scanning [51]. In *C. elegans*, RT-PCR analysis showed that the full length tRNase Z^L^ and its splice variants that differ by the presence or absence of a an N-terminal putative MTS are likely generated by alternative splicing of 5' exons [29]. Thus, it is possible that both the nuclear and mitochondrial forms of the *Caenorhabditis* tRNase Z^L^ genes could be translated from a single mRNA species through an alternative splicing mechanism.

### The Role of the AxDx Motif

**Figure 8:**
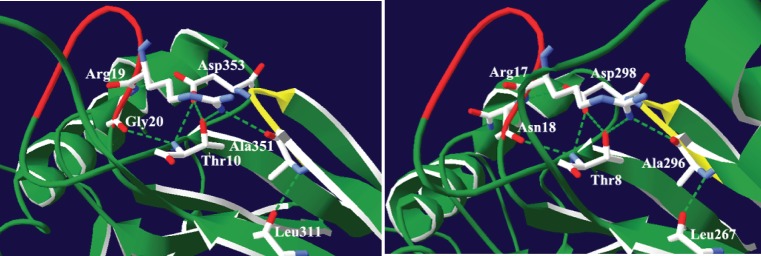
Depiction of the predicted hydrogen bonding network in the AxDx strand/loop region of human and *B. subtilus* tRNase Z^S^s. The atomic coordinates of human and *B. subtilus* tRNase Z^S^s were obtained from the Protein Data Bank, www.rcsb.org (PDB accession codes 3ZWF and 1Y44, respectively). Images were made with Swiss-PdbViewer [59]. Potential hydrogen bonds were determined using the Swiss-PdbViewer and Insight II. Secondary structure elements are colored green. The AxDx strand/loop is colored yellow and the PxPxRG or PxKxRN loop is colored red. Hydrogen bonds are represented by a dashed green line. (A) In the human tRNase Z^S^, the AxDx strand/loop-centered hydrogen bond network involves Ala351 (O) and Arg19 (NH1), Asp353 (OD2) and Gly20 (HN), Ala351 (HN) and Leu311 (O), Asp353 (OD1) and Thr10 (HN and OG1), and Gly20 (O) and Thr10 (HN). (B) In *B. subtilus*, the AxDx strand/loop-centered hydrogen bond network includes Ala296 (O) and Arg17 (NH1), Asp298 (OD2) and Asn18 (HN), Ala 296 (HN) and Leu267 (O), Asp298 (OD2) and Thr8 (HN and OG1), and Asn18 (O) and Thr8 (HN).

Our sequence analysis revealed an AxDx sequence motif, which is conserved in the eukaryotic tRNase Z^L^s and bacterial tRNase Z^S^s, but not in the TM-type tRNase Z^S^s. In the crystal structures of human and *B. subtilus* tRNase Z^S^s, the two AxDx motifs have entirely the same folding topology (http://www.rcsb.org/pdb/explore.do?structureId=3ZWF and [18]). The first residue of the motif (Ala) is located at the end of *β*15 while other residues are found in a short loop connecting *β*15 and *β*16. We noted that the AxDx motifs of human and *Bacillus* tRNase Z^S^s and the PxKxRN motif or its variant PxPxRG are in close enough proximity to make hydrogen bonding interactions (Figure 8). The PxKxRN motif or its variant PxPxRG forms part of a loop which acts like a flap covering the entrance to the active site. Strikingly, the hydrogen bonding networks formed by the AxDx motif and the PxPxRG or PxKxRN motif are very similar with one notable difference: the OD2 atom of Asp353 and the main-chain amino group of Thr10 in the human tRNase Z^S^ make hydrogen bonds with the main-chain amino and carbonyl groups of Gly20 of the PxPxRG motif, respectively, whereas the corresponding residues in the *Bacillus* tRNase Z^S^ form hydrogen bonds with the main-chain amino and carbonyl groups of Asn18 in the PxKxRN motifs (Figure 8). Interestingly, our homology modeling of the human tRNase Z^L^ based on the crystal structure of the human tRNase Z^S^ suggests that the potential hydrogen bonding network involving the AxDx motif is likely to be conserved in the structure of the human tRNase Z^L^ (Figure S4).

The PxKxRN motif, in particular, the two basic residues in the motif, has been proposed to may play a role in cleavage site specificity and, by inference, CCA anti-determination, which prevents removal of mature 3′-end of tRNAs by tRNase Z, and thus avoids the repeated addition and removal of the CCA sequence [17], [18]. In *D. melanogaster*, alanine substitutions of conserved residues in the PxKxRN motif significantly increased the Km value [17]. Considering its location and the possible role of the PxKxRN motif, we speculate that the AxDx motif may be indirectly involved in cleavage specificity through stabilization of an appropriate conformation of the PxKxRN/PxPxRG loop for tRNA processing.

Using a *S. pombe*-based functional complementation assay we have demonstrated the importance of the conserved Asp in the AxDx motif for the function of the *S. pombe* nuclear tRNase Z^L^. However, its exact role in tRNA processing is unclear and awaits further biochemical characterization.

### Alternative Splicing

Our database searches revealed candidate alternatively spliced isoforms of tRNase Z^L^ in mammalian and nematode species. Of the types of alternative splicing of the mammalian tRNase Z^L^, skipping of exon 7 appears to be the most common type of alternative splicing. This exon is skipped due a downstream splice site of exon 8. The sizes of exon 7 of mammalian tRNase Z^L^s range from 108 (in dog) to 135 bp (in rat). The region encoded by exon 7 is located upstream from the exon 8 encoding the flexible arm. Further, the region encoded by exon 7 is not found in the fly and nematode tRNase Z^L^s. Thus, exon 7 apparently does not required for the tRNA 3′-end processing activity of the metazoan tRNase Z^L^.

So far, alternative splicing of the mammalian tRNase Z^L^ mRNA has only been experimentally verified in mouse and rat tissues [33]. The mouse and rat tRNase Z^L^s have two mRNA splice forms. Real-time quantitative PCR analysis revealed that both splice forms of tRNase Z^L^ are widely expressed in most mouse and rat tissues. In the mouse, the highest levels of expression of the two splice forms (MmuTRZ2 and MmuTRZ2i) were detected in the testis and spleen, respectively, whereas the lowest levels of MmuTRZ2 and MmuTRZ2i were observed in both the prostate and muscle, and the muscle, respectively. In the prostate and muscle, the MmuTRZ2i mRNA is the predominant transcript, whereas in other tissues, the MmuTRZ2 transcript is predominant. Similarly, in the prostate, the rat tRNase Z^L^ expression is dramatically reduced and the alternative form of tRNase Z^L^ is more abundant than the normally spliced form. However, the spliced variants of mouse and rat tRNase Z^L^s have been experimentally validated on the mRNA level, but not on the protein level. Furthermore, the function of these spliced variants of tRNase Z^L^ remains to be characterized.

### Conclusions

The present study complements our previous work by identifying and analyzing metazoan tRNase Zs. We found that although both tRNase Z^S^ and tRNase Z^L^ arose very early in metazoan evolution, only tRNase Z^L^ has been retained throughout metazoan evolution. Similar to the situation in fungi and plants, in metazoans, a single tRNase Z^L^ seems to be responsible for both nuclear and mitochondrial tRNA 3′-end processing. In contrast, tRNase Z^S^, which is retained only in deuterostomes, lophotrochozoans and lower metazoans, may have a function other than tRNA 3′-end processing. We also found the existence of the predicted alternative splice variants of the mammalian and nematode tRNase Z^L^ genes in the GenBank database. Using sequence conservation analysis, we identified a conserved AxDx motif in all metazoan tRNase Zs examined. We predicted that potential hydrogen bonding interactions involving the AxDx and PxKxRN/PxPxRG motif are a highly conserved structural feature of tRNase Zs. We showed that the AxDx motif is important to the function of the *S. pombe* tRNase Z^L^ in a functional complementation assay. Our results should open up interesting new perspectives for future studies of tRNase Z.

## Methods

### Metazoan Genome Database Searches and Protein Sequence Analyses

Candidate tRNase Zs were identified by blastp and tblastn searches of genomic, protein and EST databases, which include the National Center for Biotechnology Information nonredundant protein sequence database (NCBI; http://www.ncbi.nlm.nih.gov/sutils/genom_table.cgi?organism=euk), Metazome (http://www.metazome.net/search.php?show=blast), Ensemble (http://www.ensembl.org/index.html), ExPASy (http://www.expasy.ch/), the Joint Genome Institute (JGI; http://www.jgi.doe.gov/), the Broad Institute (http://www.broadinstitute.org/scientific-community/data), Wormbase (http://www.wormbase.org/), the Universal Protein Resource (UniProt; http://www.uniprot.org/), and SpongeBase (http://spongebase.unimainz.de/), using default parameters. The resulting sequences were subject to validation as described [5], [7]. Briefly, each prediction was first subjected to reciprocal BLAST searches against the GenBank database. In back-searches, a candidate was confirmed if the best hits from reciprocal BLAST are tRNase Zs. Accuracy of prediction was further evaluated by multiple sequence alignment. All discordant candidate sequences were checked manually for possible errors including sequencing errors, intron mispredictions and existence of gaps in the genome sequences. When possible, we also used mRNA/EST sequences to check the predicted intron positions. We kept the candidate if it could be accurately predicted, otherwise we discarded it. However, despite our best efforts, we could not conclusively rule out the possibility that some of candidates might contain errors. The splicing pattern was verified using the Fgenesh and Fgenesh_GC programs provided at the Softberry website (http://linux1.softberry.com/berry.phtml?topic=fgenesh). Prediction of subcellular localization of proteins was made using web-based prediction programs such as MITOPROT (http://ihg2.helmholtz-muenchen.de/ihg/mitoprot.html) and PSORT (http://psort.hgc.jp/form.html). Multiple sequence alignments were done by Clustal W [52].

### Phylogenetic Analysis

The phylogenetic tree for candidate metazoan tRNase Zs was constructed as described [5], [7]. Accession numbers for the sequences used in the analysis are summarized in Table S1. Briefly, a multiple amino acid sequence alignment of metazoan tRNase Z^S^s and C-terminal halves of metazoan tRNase Z^L^s was generated with Clustal W implemented in MEGA5.0 [53] were manually adjusted. Model selection was performed using ProtTest 2.4 [54]. The Bayesian phylogenetic tree was inferred by using MrBayes version 3.1.2 [55] and a mixture of the fixed amino acid models and I + G distribution. Statistical confidence was assessed by using Markov Chain Monte Carlo (MCMC) sampling approaches. Two separate runs including a total of four independent tree searches were conducted. All searches consisted of one ‘cold’ and three ‘heated’ Markov chains estimated for 10^7^ generations, and every 100 generations were sampled. The burn-in parameter was estimated by plotting -ln*L* against the generation number using Tracer (v1.4.1, http://beast.bio.ed.ac.uk/Tracer), and the retained trees were used to estimate the consensus tree and the Bayesian posterior probabilities.

### PCR Mutagenesis

The aspartate to alanine mutation in the ^770^AxDx^773^ motif of SpTrz1 was generated by the overlapping PCR method [56]. Briefly, the plasmid expressing wild type Sptrz1 [57] was used as template for PCR. The upstream region of the mutant was generated using the following pairs of primers: 5'-GACTGAGTCGACATGGATTACAAAGACGATGACGAC (designated SalI) and 5'-GACTTACATATAGCTTTGGCGTTTGCTGGAATGACTCTTAAAATATCGG (designated spTrz1-D-fo). The downstream region of the mutant was created using the following primer pairs : 5'-CCGATATTTTAAGAGTCATTCCAgCAAACGCCAAAGCTATATGTAAGTC (designated spTrz1-D-re) and 5'-ACGTCGTCCCGGGTTAGAACTTTAAAGGATCCGACTCCTC (designated spTrz1-re). The up and downstream fragments of the mutant from the first round of PCR were mixed in a 1∶1 molar ratio, diluted and used as template for the second round of PCR. The primer pair SalI and spTrz1-re was used in the second round of PCR. The resulting PCR product containing mutated *sptrz1* (*sptrz1-D772A*) was digested and cloned into the Sal I/Sma I sites of pREP4X. The point mutation was verified by DNA sequencing.

## Supporting Information

Figure S1 **Alignment of candidate tRNase Z^L^s identified in metazoans.** The accession numbers for the candidates are listed in Table S1. The annotation of the alignment is described in the legend to Figure 3.
(DOC)

Figure S2 **Alignment of candidate tRNase Z^S^s identified in metazoans.** The accession numbers for the candidates are listed in Table S1. The annotation of the alignment is described in the legend to Figure 3.
(DOC)

Figure S3 **Comparison of the sequences flanking the two in-frame AUG start codons in 58 metazoan tRNase Z^L^ mRNAs.** Sequences were aligned by the start codon. The AUG start codons are indicated in red. In each sequence, nucleotides at positions −3 and +4, which are the most important determinants of context are shown in blue and green, respectively. Although the consensus sequences surrounding AUG start codons vary considerably between eukaryotic groups, they share a strong preference for purines at the position −3 and G at the position +4 for translational initiation.
(DOC)

Figure S4 **The predicted hydrogen bonding network in the AxDx strand/loop region of human tRNase Z^L^.** The published structure of human tRNase Z^S^ (PDB code 3ZWF) was used as a template to build a modeled structure of C-terminal region of human tRNase Z^L^ (residues 481–754) using the homology modeling server SWISSMODEL (http://swissmodel.expasy.org/). The picture is labeled as described in the legend to Figure 8. The potential hydrogen bond network is formed by the O atom of Ala746 and the NH1 atom of Arg497 (3.07Å), the OD2 atom of Asp748 and the HN atom of Asn498 (2.78Å), the HN atom of Ala 746 and the O atom of Leu722 (2.86Å), the OD2 atom of Asp748 and the HN atom of Thr488 (3.06Å), and the OD2 atom of Asp748 and the OG1 atom of Thr488 (2.71Å), the OG1 atom of Thr488 and the NH1 atom of Arg497 (2.81Å) and the O atom of Asn498 and the HN atom of Thr488 (3.01Å). The figure was prepared using Swiss-PdbViewer [59].
(TIF)

Table S1 **Distribution of candidate tRNase Zs identified in metazoans.** Abbreviations for species names are indicated in the parentheses. **^+^**The number of amino acids in metazoan tRNase Z and tRNase Z-like proteins. *Indicates that mispredicted sequences obtained from the databases have been corrected. ^?^Indicates the sequence could not be correctly predicted.
(DOC)

Table S2 **Prediction of the number of introns in metazoan tRNase Z genes.**
(DOC)

Table S3 **Subcellular localization prediction of metazoan tRNase Z^L^s.** The putative NLSs were predicted using PSORT (http://psort.hgc.jp/form.html), while the putative MTSs were predicted using MITOPROT (http://ihg2.helmholtz-muenchen.de/ihg/mitoprot.html). Nuc: stand for the nucleus; Mito: mitochondria. N indicates nuclear localization and M denotes mitochondrial localization. “-”, the localization or targeting sequence could not be predicted. The numbers refer to amino acid positions starting from the N-terminus.
(DOC)

Table S4 **Percentage amino acid identity among tRNase Z^L^s from selected metazoans.** The pairwise percent identity scores were generated with Clustal W [52]. tRNase Z^L^s are from *H. sapiens* (Hsa), *C. familiaris* (Cfa), *M. musculus* (Mmu), *R. norvegicus* (Rno), *O. cuniculus* (Ocu), *S. scrofa* (Ssc), *A. carolinensis* (Aca), *X. tropicalis* (Xtr), *D. rerio* (Dre), *G. aculeatus* (Gac), *C. intestinalis* (Cin), *C. savignyi* (Csa), *B. mori* (Bmo), *D. melanogaster* (Dme), *C. elegans* (Cel), *H. robusta* (Hro), *S. mansoni* (Sma), *T. adhaerens* (Tad), *M. brevicollis*, (Mbr), *A. thaliana* (Ath), and *S. pome* (Spo).
(DOC)

Table S5 **Percentage amino acid identity among tRNase Z^S^s from selected metazoans.** The pairwise percent identity scores were generated with Clustal W [52]. *H. sapiens*, Hsa; *M. musculus*, Mmu; *R. norvegicus*, Rno; *O. cuniculus*, Ocu; *S. araneus*, Sar; *T. syrichta*, Tsy; *A. carolinensis*, Aca; *X. tropicalis*, Xtr; *D. rerio,* Dre; *G. aculeatus*, Gac; *C. intestinalis*, Cin; *C. savignyi*, Csa; *B. floridae*, Bfl, *L. gigantean*, Lgi; *S. mansoni*, Sma; *A. queenslandica*, Aqu; *N. vectensis*, Nve; *M. brevicollis*, Mbr; *B. subtilis*, Bsu; *E. coli*, Eco.
(DOC)
