# Supplementary material for: Identification and Sequence Analysis of Metazoan tRNA 3′-End Processing Enzymes tRNase Zs
Source: PLoS One. 2012 Sep 4;7(9):e44264. doi: 10.1371/journal.pone.0044264 (PMC3433465; doi:10.1371/journal.pone.0044264)
Supplement: Figure S1 — Alignment of candidate tRNase ZLs identified in metazoans. The accession numbers for the candidates are listed in Table S1. The annotation of the alignment is described in the legend to Figure 3. (DOC) [file pone.0044264.s001.doc]

Figure S1: Alignment of candidate tRNase Z^L^s identified in metazoans

**BtaTRZ2 (1) --------------------------------------------------------------------MWALRS------------LLGLRCAAGRTMSQG-----------PARRPRPP**

**CfaTRZ2 (1) --------------------------------------------------------------------MWLLRS------------LLLLRSAAGRAMSQG-----------PARRQRPP**

**CpoTRZ2 (1) --------------------------------------------------------------------MWALR--------------SVLRSVAGRTMSQG-----------PARRQRPP**

**CjaTRZ2 (1) --------------------------------------------------------------------MWALC--------------CLLRFAARRTMSQERTL-----SQGSARRQRPP**

**EcaTRZ2 (1) --------------------------------------------------------------------MWALR-------------SLLLRAAAGRAMSQG-----------PARCQRPP**

**LafTRZ2 (1) --------------------------------------------------------------------MWALR--------------SLLRAAAGGTMSQG-----------PARRQRPA**

**LgoTRZ2 (1) --------------------------------------------------------------------MWALC--------------SLLRSAAGRTMSQGRTI-----SQAPARRERPR**

**HsaTRZ2 (1) --------------------------------------------------------------------MWALC--------------SLLRSAAGRTMSQGRTI-----SQAPARRERPR**

**MmuTRZ2 (1) --------------------------------------------------------------------MWALR--------------SLLRPLGLRTMSQG-----------SARRPRPP**

**PtrTRZ2 (1) --------------------------------------------------------------------MWALC--------------SLLRSAAGRTMSQGRTI-----SQAPARRERPR**

**OcuTRZ2 (1) --------------------------------------------------------------------MWTLR--------------SLWRSVAGRTMSQG-----------PARRQRPP**

**RnoTRZ2 (1) --------------------------------------------------------------------MWALR--------------SLLRPLGLRTMSQG-----------SARRPRPP**

**SscTRZ2 (1) --------------------------------------------------------------------MWALRS------------LLGLRSVAGRTMSQG-----------PARRQRPP**

**GgaTRZ2 (1) ----------------------------------------------------------------MWRLAWTLR--------------RGLAWGAGTVMAEG-----------PSAARRP-**

**AcaTRZ2 (1) --------------------------------------------------------------------MWRRL--------------LGSVAGAGTTMSVEG---------APCRRPRPP**

**XtrTRZ2 (1) ---------------------------------------------------------------------MWLRLA----------LCERPHGLSRFAMSEPSNL-----VRAFSSRKDKP**

**DreTRZ2 (1) -----------MRDIRTESTCVLPPSHALSSSPRVGSVCVHSINTRESFKPLFFRQLDLETFVLFIGQTLGFRC-----------SAPRSRALYCLCIMLARLR-----RSLSVFLMDAH**

**GacTRZ2 (1) ------------------------------------------MNAVR-EKLGSIPLLFIKGRSLSAGPPAVPSC-----------YFPVPRTVLQFFRTMASNE-----NKTPETRQPAP**

**OlaTRZ2 (1) ------------------------------------------MNAVHHVKVRFFTSLLRFGQSLSVGPSIFS-------------PPSLRRTVLWGFRTMASVG-----DRLPETSQAPP**

**SsaTRZ2 (1) ------------------------------------------MFSMTKVSLPCKSIAWLFVRHTSTGRLQSFACS----------EHRFPRTIFLFLRTMSTNT-----TDNQHRGSKKP**

**TruTRZ2 (1) ----------------------------------------------------MIRGHLKLLTLPFTGVLSAARC-----------HCPPHRTVFQLFRTMASTS--------PESWQPQR**

**TniTRZ2 (1) ----------------------------------------------------MSPGCLRLFLG--AGRLPAARC-----------RLPSHSSHSSHSSPTMSSS-----N--LEGWQPPR**

**CinTRZ2 (1) -------------------------------------------------------------------------------------------------------------MMMSCRNVVRS**

**CsaTRZ2 (1) ------------------------------------------------------------MFCCLKTSPFLKHFNILNHSCYTCWNNGGINLVNGVRNS-S--------INFRRNYSKLD**

**BflTRZ2 (1) ------------------------------------------------------------------------------------------------------------------------**

**SpuTRZ2 (1) MYLSSFSVSTARCCKNVIIRNTLSKNGIACPSFTFSCKHSSTSSTSLSLGLLSRSRLKIVTENTNFLSICNVRRISSGKNWLNLRCTKTFYSDPFQSESQIRLSRSKWSFSSQFGIQRRA**

**SkoTRZ2 (1) --------------------------------------------------------------------------------------------------------------MAVASISRIT**

**AaeTRZ1 (1) --------------------------------------------------------------------MYKLLTS----------LRPNVVGS-ASVIR----------FYSSNLKPQRK**

**AdaTRZ1 (1) --------------------------------------------------------------------MYSVFRL----------VNPYVVSHYSISRR----------WYSINEKLRKV**

**AecTRZ1 (1) -----------------------------------------------------------------MRSNVCSFAC----------HAIKHNYRTLLVFPVGRSLRHNLSVSYKQFSSIHN**

**AgaTRZ1 (1) --------------------------------------------------------------------MYTISRL----------VNSNVVRS-KVPVR----------WNSAHKKLNKL**

**ApiTRZ1 (1) -------------------------------------------------------------------------------------------------MS----------LKLYNKLLLKF**

**BmoTRZ1 (1) ---------------------------------------------------------------------------------------------------------------MPKAQTARI**

**CflTRZ1 (1) ------------------------------------------------------------------------------------------------------------------------**

**CpiTRZ1 (1) ------------------------------------------------------------------------------------------------------------------------**

**CquTRZ1 (1) ------------------------------------------------------------------------------------------------------------------------**

**DpuTRZ1 (1) ----------------------------------------------------------------------------------------------------------------MPKDTSHV**

**DanTRZ1 (1) --------------------------------------------------------------------MFVVKSR----------CWPLYGTIRTFKMS----------SSVAATIASAT**

**DerTRZ1 (1) --------------------------------------------------------------------MYLVKSA----------GSPIYRTLRTLSTR----------NLMAATIASAK**

**DgrTRZ1 (1) ------------------------------------------------------------------MWLPLLKLQ----------RIALHRTLRKLTKH----------SIRMSISSSST**

**DmeTRZ1 (1) --------------------------------------------------------------------MYLVKSA----------RSPIYRTLRTLTTS----------NLMAATIASAK**

**DmoTRZ1 (1) ----------------------------------------------------------------------MLKLQ----------RIVPHGPLRSLTNH----------CIRMSSTVSTA**

**DsiTRZ1 (1) --------------------------------------------------------------------MYLVKSA----------GSPIYRTLRTLTTS----------NLMSATIASAK**

**DseTRZ1 (1) --------------------------------------------------------------------MYLVKSA----------GSPIYRTLRTLTTS----------NLMSATIASAK**

**DviTRZ1 (1) ------------------------------------------------------------------MWLAMLKLQ----------RIAPHGKLRTLTEN----------CIRMSSQVVTD**

**DwiTRZ1 (1) --------------------------------------------------------------------MIFLKFP----------RPLLYGQHLRRRQQ----------IHRLINMSSSV**

**DyaTRZ1 (1) --------------------------------------------------------------------MYLVKSA----------GSPILRTLRTLTTC----------NLMSAPIASAK**

**HmeTRZ1 (1) -------------------------------------------------------MYGKIKIANRVVRIYNIRYY----------NKDSSKKLLEFLGT----------MPKVSGKDGRI**

**NviTRZ1 (1) -------------------------------------------------------------------MIFSKFYC----------LTTFHR-RTTIIIR-----------QYAKYQSFRP**

**TcaTRZ1 (1) --------------------------------------------------------------------------------------MSSRVSFIQARRY----------AKVLNSNLINI**

**AsuTRZ1 (1) -------------------------------------MSLCCSSFVLLQGSCFGKTIVALLRPTKLLRSLSSRSRYSPP----HSEEITEKIYEDLNQRIDKFRKELNKYARASFRSRSA**

**BmaTRZ1 (1) ----------------------------------------------------------------MYILLPAKKER------------DTEEIIQALNRRISEYRKYLSRHSNSTYLSKKA**

**CbrTRZ1 (1) -----------------------------------MKICLNLIFSCFEKTSVQIVISQLYSILLFRIPRNSVSTT---------ARTFNEDLLESIKERIARNRRILQKHSSSHLKAREV**

**CreTRZ1 (1) ----------------------------------------------------MRRAVSRIVENRTLLTRFFSSTS---------PLFNNEDLIESIRERIARNRRILQKHSSSHLKAREV**

**CelTRZ1 (1) ---------------------------------------------------MLGAIARKTVENRILVSRHLISSTSCL------FKDNNEELLESIKERIARNRRILQKHSSSHLKAREV**

**LloTRZ1 (1) -----------------------------------------------------------------MISLSVKKEQ------------DTEAIIQALNQRIGEYRKYLVRHSKSTYLGKKA**

**PpaTRZ1 (1) ------------------------------MLISLHEIFTCCIESLPPLFSSFARTHGYIVDRRYYSSRKVEKEVA--------QDSRLDREIADIQQRVHDYKKQLRRHVVSSFKSREL**

**TspTRZ1 (1) -------------------------------------------------------------------------------------MIYFQQLIFALTKKRCSIWP-----SSSSVWLNIR**

**WbaTRZ1 (1) ----------------------------------------------------------------MYVPLPAKKEQ------------DTEEIIQALNRRISEYRKYLSRHSNSTYLGKKA**

**CteTRZ1 (1) ------------------------------------------------------------------------------------------------------------------------**

**LgiTRZ2 (1) ---------------------------------------------------------------------------------------------------------------MENLNVQPK**

**LgiTRZ3 (1) ---------------------------------------------------------------------MGLKLG----------TSGESFVLCKFHKIQTRTYSLKSGKVIPSETWYGE**

**HroTRZ1 (1) ---------------------------------------------------------------------------------------------------------MINFNFLPSKLKIVT**

**ShaTRZ2 (1) -----------------------------------------------------------------------------------------------MGRRKG--------KAGKGAPHHKN**

**SmaTRZ2 (1) -----------------------------------------------------------------------------------------------------------------MLRPSLC**

**NveTRZ3 (1) --------------------------------------------------------------------------------------------------------------MLSCRVIQSY**

**NveTRZ2 (1) -----------------------------------------------MIVGFLQCRTPCFRGFTFKSCTGRIEELFTRRYNNRCLQFDRARHPLRGTEGISLASPRRSVFRVTCRHYWNG**

**HmaTRZ2 (1) ------------------------------------------------------------------------------------------------------------------------**

**AquTRZ2 (1) ------------------------------------------------------------------------------------------------------------------------**

**TadTRZ1 (1) ------------------------------------------------------------------------------------------------------------------------**

**MbrTRZ2 (1) ---------------------------------------------------------------------------------------------------------------MWGRRLVWH**

**ψ Motif I ψ Motif II**

**BtaTRZ2 ( 30) ---------------KDPLRHLRTREKRG--PSW---GPGGPNTVYLQVVAAGGRDAGAALYVFSEFN-RYLFNCGEGVQRLMQEHKLK--VSRLDNIFLTRMHWSNVGGLCGMILTLKE**

**CfaTRZ2 ( 30) ---------------KDPLRHLRTREKRG--AAW---EPGGPNTVYLQVVAAGGRDVGAALYVFSEYN-RYLFNCGEGVQRLMQEHKLK--VARLDNIFLTRMHWCNVGGLCGMILTLKE**

**CpoTRZ2 ( 28) ---------------KDPLRHLRTREKRG--ASW---GPGSPNTVYLQVVAAGGRDAGAALYVFSEYN-RYLFNCGEGVQRLMQEHKLK--VARLDNIFLTRMHWCNVGGLCGMILTLKE**

**CjaTRZ2 ( 34) ---------------KDPLRHLRTREKRG--PPG---CSESPNTVYLQVVASGRRDAGAALYVFSEYN-RYLFNCGEGVQRLMQEHKLK--VARLDNIFLTRMHWSNVGGLCGMILTLKE**

**EcaTRZ2 ( 29) ---------------KDPLRHLRTREKRG--PSW---EPGGPNTVYLQVVAAGGRDADPALYVFSEYN-RYLFNCGEGVQRLMQEHKLK--VARLDNIFLTRMHWCNVGGLCGMILTLKE**

**LafTRZ2 ( 28) ---------------KDALRHLRTREKRG--LSW---EPGGPNTVYLQVVAAGSRDAGAALYVFSEYN-RYLFNCGEGVQRLMQEHKLK--VARLDNIFLTRMHWCNVGGLCGMILTLKE**

**LgoTRZ2 ( 34) ---------------KDPLRHLRTREKRG--PSG---CSGGPNTVYLQVVAAGSRDSGAALYVFSEFN-RYLFNCGEGVQRLMQEHKLK--VVRLDNIFLTRMHWSNVGGLSGMILTLKE**

**HsaTRZ2 ( 34) ---------------KDPLRHLRTREKRG--PSG---CSGGPNTVYLQVVAAGSRDSGAALYVFSEFN-RYLFNCGEGVQRLMQEHKLK--VARLDNIFLTRMHWSNVGGLSGMILTLKE**

**MmuTRZ2 ( 28) ---------------KDPLRHLRTREKRG--P-----GPGGPNTVYLQVVAAGGRDAGAALYVFSEYN-RYLFNCGEGVQRLMQEHKLK--VARLDNIFLTRMHWSNVGGLCGMILTLKE**

**PtrTRZ2 ( 34) ---------------KDPLRHLRTREKRG--PSG---CSGGPNTVYLQVVAAGSRDSGAALYVFSEFN-RYLFNCGEGIQRLMQEHKLK--VARLDNIFLTRMHWSNVGGLSGMILTLKE**

**OcuTRZ2 ( 28) ---------------KDLLRHLRAREKRV--LSW---VPGGPNTVYLQVVAAGGRDAGAALYVFSEYN-RYLFNCGEGVQRLMQEHKLK--VARLDNIFLTRMHWANVGGLCGMILTLKE**

**RnoTRZ2 ( 28) ---------------KDPLRHLRTREKRG--PGW---GPGGPNTVYLQVVAAGGRDAAAALYVFSEYN-RYLFNCGEGVQRLMQEHKLK--VARLDNIFLTRMHWSNVGGLCGMILTLKE**

**SscTRZ2 ( 30) ---------------KDPLRHLRTREKRG--PSW---APGGPNTVYVQVVAAGGRDAGAALYVFSEYN-RYLFNCGEGVQRLMQEHKLK--VSRLDNIFLTRMHWANVGGLCGMILTLKE**

**GgaTRZ2 ( 31) ---------------KDVPRHVWARERRR--SAGTG--LSGPNTVYVQVVAAGSRDAGASVYVFSEFN-RYLFNCGEGTQRAMQEHKLK--ISHLDSIFLSRVAWANVGGLPGMILTLKA**

**AcaTRZ2 ( 30) ---------------KDALRHLRSREKRL--GAPGACSKGGASTVFVQVAAAGTRDVGAALYVFSEYN-RYLFNCGEGVQRLMQEHKLK--VARLDNIFLTRMNWANVGGLSGMILTLKE**

**XtrTRZ2 ( 37) --------------PKDTLRHIKRREKR---QGVGQ--SNGPATVYVQVAGAGSRDSGASVYVFSEFN-RYLFNCGEGTQRLMQEHKLK--IARLDNIFLTRMNWANVGGLSGLILTLRD**

**DreTRZ2 ( 94) T-------SGRSKTLKETRRPLKTRDNR----RQ--ADNHGPANVYVQVVGAGSRDNGASLYVFSEFN-RYLFNCGEGTQRLMQEHKLK--IARLDNIFLTRMSWDTVGGLSGMILTLKD**

**GacTRZ2 ( 62) ----------KKAKQKEPLRRVKTKEDR---VRRGE--GHGPSTVYVQVVGAGSRDNSASLYVFSEFN-RYLFNCGEGTQRLMQEHKLK--AARLDNIFLTRLSWENVGGLSGMILTLKD**

**OlaTRZ2 ( 61) R-------AAKKAKQKEPLRRVKTKESR---VRRGD--ANGPSTVYVQVVGAGSRDNAACLYVFSEFN-RYLFNCGEGTQRLMQEHKLK--AARLDNILLTRLSWENLGGLSGMILTLKD**

**SsaTRZ2 ( 64) ------------RVPKETLRHVKSREQR---KRGVD--VHGPATVYAQVVGAGSRDNGASLYVFSEFN-RYLFNCGEGTQRLMQEHKLK--AARLDNIFLTRMSWENVGGLSGMILTLKD**

**TruTRZ2 ( 50) -----------KVKKKEPLRLIKSKESR---SKRTD--LNGPSTVYLQVLGAGSRDNPASIYVFSEFN-RYLFNCGEGTQRLMQEHKLK--ATHLDNIFLTRMNWQNVGGLSGMILTLKD**

**TniTRZ2 ( 49) -----------KARHKEPLRRVKCKENR---SRRPD--ANGPSTVYLQVLGAGSRDNPASVYVFSEFN-RYLFNCGEGTQRLMQEHKLK--ATHLDNIFVTRMNWQNVGGLSGMILTLKD**

**CinTRZ2 ( 12) C-----------CHANTDLRHCFKKFCSTVKVKEKVN-----KEKKGVFQILRSEVFTTPCVAIPGRSDWSLFNLSGNVHRNCILSKKR--FQRTKRCFFTSLNWNN--VVPGLVQVVSG**

**CsaTRZ2 ( 52) D------TEVTTQTRIDPSHSNPPSTPQVPPTPLTNDPASRPVSFSVLKSEIGSNPGLSIRPRGYAERDAICINMPAYVQRNCVKHRIQIPHSPSP-CLLTNLEWG--ALVPGLCAAMNS**

**BflTRZ2 ( 1 ) -------------MAEETLRHVKKRKKG-------QTYVNNIANFYLQVIGAGSRDTCPSLFVFSDTK-RYLFNCGEGTQRIMMEHKVK--VSRMEDIFLTRLSWNNVGGLGGMTLTLQS**

**SpuTRZ2 (121) A-----SSHWEMAADKAKLRFLKNRERRAQLDKAPQ----QSGVVSLQIVGNGSIDCPPSVLIITDTS-RYIFNCGEGTQRLLMECGTRN-LSKLEHLFLTRMSWENVGGAIGMTITLKN**

**SkoTRZ2 ( 11) K-------IIIRNRHLNCVRSVFTRRNRR-HPNENMSGDGAAATCFLQVVGSGPAHAPPALFLFTDFTNRYLINCGEGTQRMMTEHKMK--LSTCDTIFLTSLKWSSIGGLVGMTLTLKD**

**AaeTRZ1 ( 32) S-PKMPLDPKHIAEAQKQRLKLKQKIIKA--SPGI---------VNLQIVGSGAPGAPASVYLFSDQT-RYLFNCGEGTQRLAYEHKTK--LSCLENIFMTRTCWERIGGLPGICLTMQD**

**AdaTRZ1 ( 33) L-NKMPLDPKHIAEAQKQRLKLKQKVAKI--SPGI---------VNLQVLGCGSPGTPASVYLFTDQT-RYLFNCGEGTQRLAYEHKTK--LSCLENIFMTRTSWDRIGGLPGICLTMQD**

**AecTRZ1 ( 46) N-QIMPRSIEHMLGMQRMRQKLKEKSAKY--IGNTK--------VSLQILGTGAYGTSRCVYLTAGHT-RYIFNCGEGTQRLAYEHKYK--LIKLEHVFITSATWNNLGGMPGMLLTIQD**

**AgaTRZ1 ( 32) L-SKMPLDPKHIAEAQKQRLKLKQKVSKV--SPGI---------VNLQVLGCGAPGTPASVYLFTDQT-RYLFNCGEGTQRLAYEHKTK--LSCLENIFMTRTNWERIGGLPGICLTMQD**

**ApiTRZ1 ( 14) I------------HTNCQNRIKIKGKSKF--PPGI---------VKFQVLGSGANGAPRCLYLFTDHS-RYLFNCGEGTQRLAHEHKMK--LSKLEHVFITHSNWLNIGGLPGLALTVQD**

**BmoTRZ1 ( 10) M------------EIQRGRQNFSKKSEKY--SPST---------VYLQVLGSGARGVPNTLYLFSDQK-RYLFNCGESTQRLAHEHKVK--LSRLEHIFITSKTWRNIGGLPGLSLTLQD**

**CflTRZ1 ( 1 ) ----MPRSSTHVLNMQLTREKLKKKSVKY--LGNVN--------VSLQILGSGARGAPRCVYLTAGHI-SYIFNCGEGTQRLAYEHKYK--LIKLEHIFVTSTSWNNLGGMPGMLLTIQE**

**CpiTRZ1 ( 1 ) ----MPLDPKHIAEAQKQRLKLKQKVAKV--SPGI---------VNLQVVGSGAAGSPASVYLFSDQT-RYLFNCGEGTQRLAYEHKTK--LSSLENIFMTRTCWERIGGLPGICLTMQD**

**CquTRZ1 ( 1 ) ----MPLDPKHIAEAQKQRLKLKQKVAKV--SPGI---------VNLQVVGSGAAGSPASVYLFSDQT-RYLFNCGEGTQRLAYEHKTK--LSSLENIFMTRTCWERIGGLPGICLTMQD**

**DpuTRZ1 ( 9 ) A------------RLQSERQAKKITKTKY--PPGT---------VTLQVLGSGVRGAPRSLYMFTDQS-CYLFNSGEGSQRLAHEHKFK--LSKVENIFFTRTSWQNVGGLPGISLTIQD**

**DanTRZ1 ( 33) D-------PLTGPRYEREPNVLRKKLASV--VPGS---------VNLQVLGAGANGAPAAVYMFTDQA-RYLFNCGEGTQRLAHEHKTR--LSRLEQIFVTRNTWATVGGLPGLALTIQD**

**DerTRZ1 ( 33) D-------PLTGPRYEREPNVLRKKLASV--VPGT---------VNLQVLGSGANGAPAAVYLFTDQA-RYLFNCGEGTQRLAHEHKTR--LSRLEQIFLTQNTWASCGGLPGLTLTIQD**

**DgrTRZ1 ( 35) V-LTAAEQNAMPAKYERQPNVLRKKLSSV--VPGS---------VNLQVLGAGANGSPSAVYLFTDQS-RYLFNCGEGTQRLAHEHKTR--LSRLEQIFVTRNTWKTVGGLPGLALTIQD**

**DmeTRZ1 ( 33) D-------PLTGPRYEREPNVLRKKLASV--VPGT---------VNLQVLGSGANGAPAAVYLFTDQA-RYLFNCGEGTQRLAHEHKTR--LSRLEQIFLTQNTWASCGGLPGLTLTIQD**

**DmoTRZ1 ( 31) A-----AEQTPPAKYERQPNVLRKKLSSV--VPGS---------VNLQVLGAGANGAPSAVYLFTDQS-RYLFNCGEGTQRLAHEHKTR--LSRLEQIFVTRNTWTAVGGLPGLALTIQD**

**DsiTRZ1 ( 33) D-------PLTGPRYEREPNVLRKKLASV--VPGT---------VNLQVLGSGANGAPAAVYLFTDQA-RYLFNCGEGTQRLAHEHKTR--LSRLEQIFLTQNTWASCGGLPGLTLTIQD**

**DseTRZ1 ( 33) G-------SLTGPRYEREPNVLRKKLASV--VPGT---------VNLQVLGSGANGAPAAVYLFTDQA-RYLFNCGEGTQRLAHEHKTR--LSRLEQIFLTQNTWAACGGLPGLTLTIQD**

**DviTRZ1 ( 35) A--KDTLGQKPPLKYERQPNVLRKKLSSV--VPGS---------VNLQVLGAGANGAPGAVYLFTDQS-RYLFNCGEGTQRLAHEHKTR--LSRLEHIFVTRNTWTTVGGLPGLALTIQD**

**DwiTRZ1 ( 33) V--AAAATDPLPAKYTREPNVLRKKLASV--VPGT---------VNLQVLGNGANGSPSSVYLFTDQS-RYLFNCGEGTQRLAHEHRTR--LSRLEHIFVTRNTWSTVGGLPGLALTIQD**

**DyaTRZ1 ( 33) D-------PLTGPRYEREPNVLRKKLASV--VPGT---------VNLQVLGSGANGAPAAVYLFTDQA-RYLFNCGEGTQRLAHEHKTR--LSRLEQIFLTQNTWATCGGLPGLTLTIQD**

**HmeTRZ1 ( 46) A------------VSQRQRQLIAKKSEKF--GAST---------VYLQVLGSGARGAPNTLYLFTDQK-RYLFNCGEGTQRLAHEHKVK--LSKLEQIFITNKTWNNIGGLPGLSLTLQD**

**NviTRZ1 ( 32) L-IMPKDNKEYLMELQKQRKRLKAQSAKY--TPTH---------IYMQVLSNGTRAESRSLLLVTDHT-NYLFNCGEGTQRLAAEHHAK--LTKIEHVFVTSSTWENMGGIPGVALTIQD**

**TcaTRZ1 ( 25) L-KTMPKEKTHIPEAQKQRRKIKEKFSKY--SPGR---------VTLQVLGTGAEGAPRSLYVFSDQS-RYLFNCGEGTQRLAHEHKMK--LAKLEHIFITRPTWANIGGLPGAALTIQD**

**AsuTRZ1 ( 80) A--------SSLSNLRAALSDMKEKQ---RKHEAINSIALIPSNVTLEVLSNGTTHLRPCVIIRTPQK-VYLFNCPEGTTRFLPSLRLK--SLNVCDIFATRGTWDHIGGISSVLLSKEQ**

**BmaTRZ1 ( 45) L--------ASVEILTQELADIQQ-------MNKAKVTPAIPSSVSIEIISNGTAHLRPCVVIHTNYK-DYLFNCPEGTSRFLAANRLK--AINLTDIFFTRDTWEHFAGVSGLLKTPVA**

**CbrTRZ1 ( 60) N--------ASISNLRQSMASVQKKQKA-AHEPPSNSIVNIPSQVSIEVLGNGTGLLHACFILRTPLK-TYMFNCPENACRFLWQLRVR--SSSVVDLFLTSASWDNIAGISSILLSKEA**

**CreTRZ1 ( 60) N--------ASISNLRQSMASVQKKQKA-AHEPPANSIVNIPSQVSIEVLGNGTGLLRACFILRTPLK-TYMFNCPENACRFLWQLRIR--SSSVVDLFITSANWDNIAGISSILLSKES**

**CelTRZ1 ( 64) N--------ASISNLRQSMAAVQKKQKA-AHEPPANSIVNIPSQVSIEVLGNGTGLLRACFILRTPLK-TYMFNCPENACRFLWQLRIR--SSSVVDLFITSANWDNIAGISSILLSKES**

**LloTRZ1 ( 44) L--------SSVEILTKELAEIKQ-------MNKTKTPPAIPSCVSIEIISNGTTHLRPCVVIHTNYK-DYLFNCPEGTSRFLAANRLK--AINLTDIFFTRDTWEHFAGVSGLLKTPVA**

**PpaTRZ1 ( 83) S--------SSISALGRTMADAQRKKASVASAANANTLASMPAHVSVEILSNGTTHMAPCVALRTPLK-TYLFNAPEGTSRFLPALRLK--PTNINDIFVTRGVWENIAGISSILLAKES**

**TspTRZ1 ( 31) L--------LSTMPPSPESTSKRARLYRTSYTGPATAYFEVIVIRFLWIIQSGCHGRGKCLILFTTSS-VYLFNSPEGTERLFCEHGVK--NSRLNHVFYTRACWDNVNGLNGLILSLRK**

**WbaTRZ1 ( 45) L--------ASVEILTQELADIQQ-------MNKAKVSPAIPSSVSIEIISNGTTHLRPCVVIHTNYK-DYLFNCPEGTSRFLAANRLK--AINLTDIFFTRDTWEHFAGVSGLLKTPVA**

**CteTRZ1 ( 1 ) ---------------------MSGQKSSR-----------------V-MRVIGCHDHATKALVLTANQITYFFNCPEGIQRALHCNGLA--KTKEVNILVTRNIWSNIGGLIGACLFFQE**

**LgiTRZ2 ( 10) ------RKEKIGKNKKAKLRILKTRGSTN--ASSAS------NLIDVHIVGMGGKGFPKAVMINTTFT-RYLFNCGEGVQRFLLVDKLK--IAKIEHLFLTSKDWSSIGGLIGLLLSLEE**

**LgiTRZ3 ( 42) ------QKKKTKSSKVKEDQKISQTDDII--SQKAS------DSIDVHIVGKGSKGFSRAVVINTTST-RYLFNCGEGIQRFFMVDKLK--IAKIEHLFLTSNSWSNIGGLIGLLFMLEN**

**HroTRZ1 ( 16) W--------AIMKKSKAPLTTTSATNGKKQGAIKKQKTQTIPSLIEAVAIGSGHHGTPMCLVVSMSHF-KYMFNCGEGTQRIATEHKKL--KGANR-VFLTNANWRNVGGLIGLSLTFET**

**ShaTRZ2 ( 18) S--------GNVEAAANARSRTQNPSGKS--QVKTQKTHKAPSSINLVVLGTGGPGASRSLLVTSEFT-RYMFNCGEGTQRLAAMTFAK--LSGLENIFITHKSWENTGGLLGLAMTLEG**

**SmaTRZ2 ( 8 ) Y----------VRLITTPKRFLKHKRPE----DRMIPPP---HSISLTVVGNGRAGSSKSMLIDTGVC-RYLVNCGESTQRVLAEYRMK--ASRIQHVFLTRMSWDCTSGLLGVALTAKA**

**NveTRZ3 ( 11) LTKLSSVVQKCLLGRKVPVYSDKMASKTK--LLS--------NKVYLQVLGTCSLDTTPSLLLFTDSQ-RYLFNCGEGTQRLFNEQKIK--YNKLNNIFFTRICWERCGGLPGMAMTLRD**

**NveTRZ2 ( 74) P--------NSCQSLSTTCMGLRVMTSCRQYWNGPGAGRSVSSRIYLQVIGTVSSQSAPSVLLFTDSQ-RYLFNCGESIHRLSCDLDIS---MDPGRIFLTGNQWQHIGGLSYIQYQPQA**

**HmaTRZ2 ( 1 ) ------------------------MSPQL--RPS--------KNVIMQVLWTGIDSSVPSLYIFTDSK-KYLFNCGEGMQRIFTSNKMLR-FGRLDTFFMTRHEWTHMGGLPGFGMTLRD**

**AquTRZ2 ( 1 ) ------------MIPLIRFSLWRRSACGTILGRCRLLCTPISNFCELLVLGTGSSEFSPFLCLSTGSG-RYLFNCPERALLSLACNSIKP--STVNHIFLTKSSWENMGGIFSTLVKMNP**

**TadTRZ1 ( 1 ) ------------MIFKQLSRKLSRISKSMSASKLN--------KVYLQILGNGSKDAPASFLLFTENQ-RYLFNCGAGIQRIINRNRIKIHPLKLRNIFMTRADFNAVGGLPGLALTINE**

**MbrTRZ2 ( 10) S----------CGLMDAAVQRRAYRMAGGTLIDKLEVTRVLNADMSNFAQELGCRVDLAPAVLVVGHRNRYLFNCPEGIQRYCLEYKLKP--LKCSHVWATRMAWDSLGGAFGFALTYGL**

**BtaTRZ2 (127) TG--------------------VPKCVLSGPP-------------QLEKYLEAIKIFSGPLKGIDLAVRPH-S------------------APEYKDETMTVFQIPIH--CEQTSGQQPP**

**CfaTRZ2 (127) TG--------------------LPKCVLSGPP-------------QLEKYLEAIKIFSGPLKGIDLAVRPH-S------------------APEYKDETMTVFQIPIYS--ELKRAERQP**

**CpoTRZ2 (125) TG--------------------LPRCVLSGPP-------------QLEKYLEAIKIFSGPLKGIDLAVRPH-S------------------APEYKDETMTVYQVPIYS--ERGLEEHQP**

**CjaTRZ2 (131) TG--------------------LPKCVLSGPP-------------QLEKYLEAIKIFSGPLKGIELAVRPH-S------------------APEYKDETMTVYQVPIYG--EPRSGEHQP**

**EcaTRZ2 (126) TG--------------------LPKCVLSGPP-------------QLEQYLEAIKIFSGPLKGIDLAVRPH-S------------------APEYKDETMTVFQVPIYS--EQRCGEQQP**

**LafTRZ2 (125) TG--------------------LPKCVLSGPP-------------QLEKYVEAIKIFSGPLKGIDMAVRPH-S------------------APEYKDETMTVFQIPIHS--EQSCRDHQS**

**LgoTRZ2 (131) TG--------------------LPKCVLSGPP-------------QLEKYLEAIKIFSGPLKGIELAVRPH-S------------------APEYEDETMTVYQIPIHS--EQRRGRHQP**

**HsaTRZ2 (131) TG--------------------LPKCVLSGPP-------------QLEKYLEAIKIFSGPLKGIELAVRPH-S------------------APEYEDETMTVYQIPIHS--EQRRGKHQP**

**MmuTRZ2 (123) TG--------------------LPKCVLSGPP-------------QLEKYLEAIKIFSGPLKGIELAVRPH-S------------------APEYKDETMTVYQVPIHS--ERRCGKQQP**

**PtrTRZ2 (131) TG--------------------LPKCVLSGPP-------------QLEKYLEAIKIFSGPLKGIELAVRPH-S------------------APEYEDETMTVYQIPIHS--EQRRGKHQP**

**OcuTRZ2 (125) TG--------------------LPKCVLSGPP-------------QLEKFLEAMKIFSGPLKGINLAVRPH-S------------------APEYTDETMTVHQVPIYG--EWKCGERQS**

**RnoTRZ2 (125) TG--------------------LPKCVLSGPP-------------QLEKYLEAIKIFSGPLKGIDLAVRPH-S------------------APEYKDETMTVYQVPIHS--ERRCGEQEP**

**SscTRZ2 (127) TG--------------------LPKCVLSGPP-------------QLEKYLDAIKTFSGPLKGIDLAVRPH-S------------------APEYKDETMTVSQVPICREGEQAAGTPQP**

**GgaTRZ2 (129) MG--------------------LQRCVFLGPP-------------KLQNYLKAIRLFPGPLKRMDLAVQLH-T------------------EPEYKDETMTVHQIPLVG--KPLAAESTF**

**AcaTRZ2 (130) TG--------------------VPKCVLSGPP-------------QLQNYLEAIKVFSGPLKGIDLAVRPH-S------------------DSEYQDETMTVYQVLLVG--KKLQDEQRP**

**XtrTRZ2 (135) TG--------------------LPKCVLSGPP-------------QLQKYLEAIRVFSGPLQGIELAVRPY-T------------------DAMYSDDTMTVYQVPLFSSAAPKTTTVGC**

**DreTRZ2 (198) TG--------------------VHQCVLSGPP-------------QLDRFLKAIKVFSGQLEDIKLAVRPY-T------------------EPQYKDETMTVSQIPLFSDRKAESGRCSP**

**GacTRZ2 (164) TG--------------------VPECVLSGPP-------------QLKNYLNAIKSFSGPLEDIKLSVRPY-T------------------EEAYSDETMTVHQVPIFGRLRVDGRKLSP**

**OlaTRZ2 (166) TG--------------------VPECVLSGPP-------------QLEKYLNAIKSFSGPLEEIKLSVRPY-T------------------EEKYQDETMTVFQVPIFGEFRRFPSPSEA**

**SsaTRZ2 (164) TG--------------------VPEVVLSGPP-------------QLEKYVNAIRVFSGPLEEIKLAVRPY-T------------------EKQYTDDTMTVSQIPIFAQLKEDGPKCSS**

**TruTRZ2 (151) IG--------------------VPECVLSGPP-------------QLENFLDAIKSFSGPLEAIKLSVRPY-T------------------ADAYTDDTMTVHQVPIFGQSTDPGSPSGP**

**TniTRZ2 (150) IG--------------------VPECVLSGPP-------------QLENFLNAIKSFSGPLEDIKLSVRPY-A------------------AEPYTDSTMTVQQVPIFAVS---------**

**CinTRZ2 (112) QT--------------------EDEIKIYGPKG-----TFTFIRNIIPSLMSSYKADQTLQKLKVTECDDQTIE----------------------EEGMRIKMMSLKPS----------**

**CsaTRZ2 (163) FG--------------------PPVQHFYGPKG-----TFQLLQSVVPNLFSGRKSSKILQQLRVTESHDDVVTS--------------------EENGFQIKMVKVKPE----------**

**BflTRZ2 ( 98) IG--------------------VPEVRVYGPP-------------NAEDFYQALKDFTYFPKIKVDVKPYQLS--------------------PMTDDVMTVWQVPIRAPGTSSEGGLNS**

**SpuTRZ2 (230) IG--------------------IPRVTMYGPP-------------NMEEFKKAIQIFAKHEAIDINLKPYS--------------------EGPFMNDTMVVTAVPLFSTKNEDTIVSTE**

**SkoTRZ2 (121) IK--------------------MRKATYYGPS-------------GLSELLSATKAFARLAEMEIDFKTYLDLP--------------------MNDKTFNIDAVPLFGKVQ--------**

**AaeTRZ1 (137) VG--------------------VPNVTLHGPP-------------GLDELFKAMRRFVILKDMKVEAAECK-A------------------EDLFDDHVMSLKYVAINREPES-------**

**AdaTRZ1 (138) VG--------------------VPEVSLHGPP-------------GLGELFKAMRRFVILKDMKVEAAEYG-T------------------GDVYEDHVMTLRYVVISRETENS------**

**AecTRZ1 (152) VG--------------------VPKINIHCPK-------------GTMEIFNTIKKTVLLKALKINEAKCN-E------------------SEPYIDSVMSVSYVSIINSNVQESE----**

**AgaTRZ1 (137) VG--------------------VPAVSLHGPP-------------GLDELFKAMRRFVILKDMKVEASEYA-T------------------GDVYEDHVMTLRYVVINRQPASG------**

**ApiTRZ1 (108) VG--------------------VPNIELHGPK-------------GIEELFIATKRFVVLRDLKITASKSD-P------------------FKPYEDNAIVVHYVPLISNK-QCNEFRSK**

**BmoTRZ1 (104) VG--------------------VPNITLHGPE-------------GLDELYNATKRFVIMKEMNVAMAKCS-P------------------SEDFEDSVMSVKYVLLGPHESLLAKDPKP**

**CflTRZ1 (104) AG--------------------VPKIDVHGPK-------------GTVELFDAVKKFVMLQALKIQEAKCD-E------------------SEPYTDSVMSVSYVPITKSSVQEKESI--**

**CpiTRZ1 (103) VG--------------------VPSVTLHGPP-------------GLDELFKAMSRFVILKDMKVRAAECR-A------------------EDLYEDHVMTLKYVAIGRKVEEG------**

**CquTRZ1 (103) VG--------------------VPSVTLHGPP-------------GLDELFKAMSRFVILKDMKVRAAECR-A------------------EDLYEDHVMTLKYVAIGRKVEEG------**

**DpuTRZ1 (103) VG--------------------VPNITLHGPP-------------GLGELFTAASRFIILKNLQVNHVDATSP------------------ASSYEDAAMKLNYVTIMP-----------**

**DanTRZ1 (132) AG--------------------VRNVGLHGPP-------------HLNTMLQSMRRFVVLKNLQMQTIDCS-G------------------GGCFEDSILKVDSLPLASSVD--------**

**DerTRZ1 (132) AG--------------------VRDIGLHGPP-------------HLGSMLQSMRRFVVLKNLQVRPNDCS-E------------------GACFEDSILKVDSLPLICLED--------**

**DgrTRZ1 (140) AG--------------------VRNVGLHGPP-------------HLDTMLQSMRRFVVLKHLQMQTIDAT-E------------------GNHFEDSIMSVESLVLRSEQQ--------**

**DmeTRZ1 (132) AG--------------------VRDIGLHGPP-------------HLGSMLQSMRRFVVLKNLQVRPNDCS-E------------------GACFEDSILKVDSLPLINSED--------**

**DmoTRZ1 (132) AG--------------------VRNVGLHGPP-------------HLDTMLQSMRRFVVLKNLQMQTIDST-L------------------GVPFEDSILTVQPVVLRSEQQ--------**

**DsiTRZ1 (132) AG--------------------VRDIGLHGPP-------------HLGSMLQSMRRFVVLKNLQVRPNDCS-E------------------GACFEDSILKVDSLPLINSED--------**

**DseTRZ1 (132) AG--------------------VRDIGLHGPP-------------HLGSMLQSMRRFVVLKNLQVRPNDCS-E------------------GACFEDSILKVDSLPLINSED--------**

**DviTRZ1 (139) AG--------------------VRNVGLHGPP-------------HLDTMLQSMRRFVVLKNLQMQTIDST-L------------------GAQFEDSILTVQPVVLRSEQQ--------**

**DwiTRZ1 (137) AG--------------------VRQVGLHGPA-------------HLSTMLQSMRRFVVLKNLQMQTEDCT-E------------------GKTFEDSILKVEAVPVRKEKDG-------**

**DyaTRZ1 (132) AG--------------------VRDIGLHGPP-------------HLGSMLQSMRRFVVLKNLQVRPNDCS-E------------------GACFEDSILKVDSLPLNSLED--------**

**HmeTRZ1 (140) VG--------------------VPNITLHGPE-------------GLDELYNATKRFVIMKEMNVTMAQCS-P------------------SVDFEDNVMTVKYVLMAPQDNILQTDLDP**

**NviTRZ1 (137) TG--------------------VPKLNLHGPD-------------GSIDIIEATKNFITLNQLTVTAQDTT-S--------------------PFSDQTMSVTYLPIKSPKPPAPVE---**

**TcaTRZ1 (130) VG--------------------VPEITLHGPQ-------------GLEEIFIATRRFIVIKDLDIKMAQCD-E------------------NTTFEDNVLKVKYVPLK-----------R**

**AsuTRZ1 (186) GA---------------------QLTRLHGPV-------------DIKHFLECIRPFTDSDFVLAKYPAPVEE--------------RPLDIGSYKDAALTVHYLPLSGLRGA-------**

**BmaTRZ1 (147) SSSS----------------KLERIIRLHGSH-------------NKMKYFESQ-----NDYTDADFPSSVTT-------------KRPLSCGFYSDDALTVHFIPVSTFSTP-------**

**CbrTRZ1 (168) NA---------------------MPTRLHGAM-------------NIKHFLECIRPFQDSDYGSCKYPSQVEE--------------RPYTMGNYEDAGLKVTYIPLSPPLDM-------**

**CreTRZ1 (168) NA---------------------MSTRLHGAM-------------NIKHFLECIRPFQDSDYGNCKYPSQVEE--------------RPYTMESYEDAGMKVTYIPLSPPLGL-------**

**CelTRZ1 (172) NA---------------------LSTRLHGAM-------------NIKHFLECIRPFQDSDYGSCKYPSQVEE--------------RPYTMENYEDAGLKVTYIPLSPPLNI-------**

**LloTRZ1 (146) SSSS----------------KLERVIRLHGSH-------------NKTKYFESQ-----NDHTDADFPSTITT-------------KRPLSCGLYSDDALIVHFIPVSTFSAP-------**

**PpaTRZ1 (192) NS---------------------LPTRLHGAV-------------NVKHFLECIRPFQDSDFGSVKYPSSKPDPDDPIEYPSTVEECTLATHDSYEDPGLKIQYIPLLSDCIK-------**

**TspTRZ1 (140) AG--------------------CAELHLHGAQAIT----------KIVRGAEQVSDWSGPPLNVIEHCDCN--------------------DSHFEDDSIQVQYLPLVGGEE--------**

**WbaTRZ1 (147) SSSS----------------KLERIIRLHGSH-------------NKTKYFESQ-----NDYTDADFPSSVTT-------------KRPLSCGFYSDDALTVHFIPVSTFSTP-------**

**CteTRZ1 ( 80) GQ--------------------KSVVRCHGSN-------------SLKQILSNARGFSQFEHQPIVTPRTE---------------------KVFTDDFLKITYVPLMADATMKADE---**

**LgiTRZ2 (113) TG--------------------VPKITLHGPP-------------NVEGISSMCKHLGQSDGTQLEKRALT--------------------EECFEDNNFKFQYVPFFCEDE--------**

**LgiTRZ3 (145) IG--------------------VPKITLHGPP-------------NVEGISSMCKHLGQSDGTKLEKRLLT--------------------EGNYEDNNFKFQYVPFFRKKE--------**

**HroTRZ1 (124) AET--------------------KQMFIHGPPNVVCLAIFYITTACHEHHISSSRNFSKFENIEIVKEEIT--------------------SEKFTDTAMTIEYIPLSNGADDDDDVG--**

**ShaTRZ2 (125) QKNPETKVYTNRDTTQIPQDRGSSVMRLYGPE-------------GVEKITLMAKKFSVHSNMRIMRCEGE-----------------------FQDAGLTITPVIFYSDSSKDS-----**

**SmaTRZ2 (108) AG--------------------VKKLTIHGPP-------------ELERLMQLTRPFTNCKTTDIVMSEIH--------------------KKCYTDDAFRVQSFQIFKPTNVN------**

**NveTRZ3 (118) SK--------------------KTTINIYGPE-------------NLKDLMNGTRFFIFHEKMKYVCTQYN-------G----------IKDTAFTDENLTLTPVVIQG-INN-------**

**NveTRZ2 (182) LEPN-------------------AGIEVHGPAG-------------TEDFVHSLYTFSKRHLVTKPSITCFDHHP--------------SSLPLYKDDNVSIQTVILEDSKPAQG-----**

**HmaTRZ2 ( 85) MK--------------------SPEIMVHAPP-------------SMIDVITGAQTFMRFDNSGVNFKVESSFN------------------KCFTDGEMTVFPILIESEKEE-------**

**AquTRZ2 (106) ES--------------------SHSSVATGPCG-------------FTFLRDSLKSFVDFDDATFTPRSDEPT------------------TLKFNDINVTAVELQPSTLCKT-------**

**TadTRZ1 (100) EDD------------------IKTPINLWGPP-------------SLSVNVPVTWTFNLALLGHLTFTNDT---------------------PYFQDENIKLWPVVIDDVGSSLYETSED**

**MbrTRZ2 (118) AG------------------PPAKPLNFYTPG-------------DLGPYLESGKAFCGVNYMRHKDVSTVET---------------------FADDELQMRIFARPSPRS--------**

**Flexible arm**

**GP motif**

**BtaTRZ2 (193) VQSPER----LSPQPPP-LAESADGEQRPDGNG-------------------------------VGWKARGRDTSLVVAFVCKLHVKKGNFLVLKAKELGLPVGTAAIAPIIAAVK----**

**CfaTRZ2 (193) SQSPER----LSPGQSS-DSGSAENEQHLPDG--------------------------------IGQKKSGRDPTLVVAFVCKLHVKKGNFLVLKAKELGLPVGTAAIAPIIAAVK----**

**CpoTRZ2 (191) SQSPGTSPHRLSPELSS-DSGSAAEEQLLPDAN------------------------------GVNKLHCGRDPSLVVAFICKLHLKKGNFLVLKAKELGLPVGTAAIAPIIAAVK----**

**CjaTRZ2 (197) WQSPERPLGRLSPERSS-DSESSENEQLLPRG--------------------------------VSQRRGGRDPSLVVAFICKLHLKKGNFLVLKAKELGLPVGTAAIAPIIADVK----**

**EcaTRZ2 (192) SQSPGR----LSPERSS-DSRSTENEPPLPDG--------------------------------ISRTLDGRDPSLVVAFVCKLHLKKGNFLVLKAKELGLPVGTAAIAPIIAAVK----**

**LafTRZ2 (191) AQRPERPLGRLSPKQPS-NSGSAENEQHLPDG--------------------------------VSQKMDGRDPSLVVAFVCKLHMKKGKFLVLKAKELGLPVGTAAIAPIITAVK----**

**LgoTRZ2 (197) WQSPERPLSRLSPERSS-DSESNENEPHLPHG--------------------------------VSQRRGVRDSSLVVAFICKLHLKRGNFLVLKAKEMGLPVGTAAIAPIIAAVK----**

**HsaTRZ2 (197) WQSPERPLSRLSPERSS-DSESNENEPHLPHG--------------------------------VSQRRGVRDSSLVVAFICKLHLKRGNFLVLKAKEMGLPVGTAAIAPIIAAVK----**

**MmuTRZ2 (189) SQSPRTSPNRLSPKQSS-DSGSAENGQCPPEDSS----------------------------AGANRKAWGRDPSLVVAFVCKLHLRKGNFLVLKAKELGLPVGTAAIAPIIAAVK----**

**PtrTRZ2 (197) WQSPERPLSRLSPERSS-DSESNENEPHLPHG--------------------------------VSQRRGVRDSSLVVAFICKLHLKRGNFLVLKAKEMGLPVGTAAIAPIIAAVK----**

**OcuTRZ2 (191) PPSPERPLSRLSPQRSS-DSGPAGNKQRLPDGD------------------------------GVKRKASARDPSLVVAFICQLHVKKGSFLVLKAKEMGLPVGTAAIAPIIAAVK----**

**RnoTRZ2 (191) SRSPKRSPNRLSPKQSSSDPGSAENGQCLPEGSS----------------------------AGVNGKAWGRDPSLVVAFVCKLHLRKGNFLVLKAKELGLPVGTAAIAPIIAAVK----**

**SscTRZ2 (195) ASSPER----PSAQPAA-DPGSAEGEQR-PAEG-------------------------------VGQNTHGRDPSLVVAFICKLHLKKGNFLVLKAKELGLPVGTAAIAPIIAAVK----**

**GgaTRZ2 (195) PQSPGLSAQDGSSSESDTEPGSPRAAQQSLDESKEKRSPKK---------------------TDGEQKHANRHPDLVMAFLCKIHPKKGKFLVLKAQEMGLPVGTPAILPIITALK----**

**AcaTRZ2 (196) HLNSQLPGSDVDQKRIMMPGSLPEEHQYLKGDR------------------------------NGRWQIDQRDPSLVVAFICKLHPKKGNFLVLKAKELGLPVGTAAIAPIIAAVK----**

**XtrTRZ2 (203) YSPPRSPERVSTTQSQKDVEGHFRQIEGISSLKR-----------------------------EDIGRLSKRDSSLVVSFICKLHDKKGNFIVLKAKELGLPVGTAAIGPIISELK----**

**DreTRZ2 (266) RSFSPHSSQQGVLRILEDQEENQSIHTRRRSTSP-------------------------------EGKEVTRDPSLVVAYVCKLHSRTGNFLVMKAKEMGLPVGTAAIGPIIKALK----**

**GacTRZ2 (232) --PSGRSSPPSPG--RDYARGDERGEFGDYTWFT------K---------------------SWGDRRRASRDNSLLVSFICKLHPKKGNFLVAQARELGLPVGTSAIGPLIAALK----**

**OlaTRZ2 (234) --PLTVADPLPVE--GKTPPPAWKQQHPLLTELE------T---------------------YCVSLAAASADPSLTVAFVCKLHPKKGNFLVAEAKALGLPVGTAAIGPLITALK----**

**SsaTRZ2 (232) --NSGRSSPSSSPQRRELWRPEDFEDTSTDSRKER----AT---------------------SPGGKARAIRDPSLVVAFVCKLHPKKGNFLVAQAKNLGLPVGTAAIGPLIAALK----**

**TruTRZ2 (219) RSPDSHRQRAGGP--GESSRPE-RAEWSRTCFKQ------L---------------------CDVSANVGSADASLVVAFVCKLHPKKGNFLVPQARELGLPVGTAAIGPLIAALK----**

**TniTRZ2 (209) --PDNHPEPAGGP--GESSTAA-GARSGR------------------------------------TQEGSARDASLLVAFICKLHPKKGNFLVPQARELGLPVGTAAIGPLIAALK----**

**CinTRZ2 (175) -------------------------------------------------------------K---------ATAGEVVSYIIQRSYKP-TFCVEKLSKLSILHQT----PTCLEEK---M**

**CsaTRZ2 (228) -----------------------------------------------------------S-----------FLQPDLVAYIILTPLYKPLLCFHKCKDHGILFQ-----RRKLELKEDFV**

**BflTRZ2 (165) RAGSRSPDHGLSPKATSSPSQSPVVRRNSSGVSDIDSESDDS--------SR--STSPRNSPKAKRRKTSQRDESLVISYICKLKPRKGRFLSEKALRDGIKPG-----VWVSELK----**

**SpuTRZ2 (297) TDDNQGEEGCSETSDREDNVSPVPSEESGVKRKAASPLDVIADMN----------VKLQSTGASAKKRKTERLPDMTIAYVCKLHDKPGKLLVKEAVQMGLKPN-----LHFELVK----**

**SkoTRZ2 (180) -----------------------------S------------------------------------SESVPEYPLGSIIQDMEMHNKPGNILVNKAISLGLKPG-----PLYGELK----**

**AaeTRZ1 (198) ----VSNSDE--ENQDEDTTVDDTDYYAYE--KRNKAAP-------------------KPKLAMTQQDWTKRHEETVMAYICKLKLRFGQLSLEKCVDRGVTPG-----PLLGQLK----**

**AdaTRZ1 (200) ----SKEG----DEQEQDAPVDDTDYYSYERRKDSVSSKE------------------ESNQKPQGTKWIKREEESVMAYICKLKPRQGQLSLEKCVEQGVPAG-----PLLGQLK----**

**AecTRZ1 (216) -------------SIHVEKDIIDINYYDYNINSNSKRVPD--------------RIEKKSKVQKIDQKPD-NRISSVMSYICKLHPRAGTLSLQKCLEKGVKPG-----PLLGQLK----**

**AgaTRZ1 (199) ----AREAGSDEDQNQDEATVDDTDYYAYERKRETLQKPQS-----------------EAVKSVQSTNWTKREESSVMAYICKLKPRHGQLSLEKCVELGVPPG-----PLLGQLK----**

**ApiTRZ1 (175) SENSIEIDNSLAQQHTDDSLEDDTNYYDYENLLQSDKKERN-------KRKRSNSEPRTDDKKFIADSSLSRISNVAMSYVCRLKPKPGFLDLDKCVKHNVPPG-----PLLGLLK----**

**BmoTRZ1 (172) TTKKPKLDTN-TDKQFEEFIHDDTDYYRNEIRNAEAKNKVASKDKNKTRTSPEKDQIKKKTDKVLHDLQ--KQPHCSVAYICTLKRRLGTLDLAKCVERGVKPG-----PLLGQLK----**

**CflTRZ1 (170) -------------DNIGEEKIDNINYYNYTINSNGKRVFDN-------------RVEKEKKTQKVEEKSEKTRISSAMSYICRLHPRAGTLSLEKCVEKGVQPG-----PLFGQLK----**

**CpiTRZ1 (165) ----GANSDE--EDKGEETTVDDTDYYAYE--KRKKSEPKA-----------------KLQTASAKQDWTKRHEESVMAYICKLKHRFGQLSLEKCVEKGVPAG-----PLLGQLK----**

**CquTRZ1 (165) ----GANSDE--EDKGEETTVDDTDYYAYE--KRKKSEPKA-----------------KLQTASAKQDWTKRHEESVMAYICKLKHRFGQLSLEKCVEKGVPAG-----PLLGQLK----**

**DpuTRZ1 (161) --N------------------DDAKLPRSAS-------------------------------------------TV-LAYVCRLQAKQGTLIAKKCFDLGVPPG-----PLYGQLK----**

**DanTRZ1 (192) ------------------------------------------------------------------------PEKSVVNYICQLKPRPGALNLVKCVEQGVPPG-----PLLGKLK----**

**DerTRZ1 (192) ------------------------------------------------------------------------PTKSVINYICQLKPRAGALNLVKCVEQGVPPG-----PLLGQLK----**

**DgrTRZ1 (200) ------------------------------------------------------------------------PEQPVISYICALKPRPGALNLVKCVEQGVTPG-----PLLGQLK----**

**DmeTRZ1 (192) ------------------------------------------------------------------------PTKSVINYICQLKPRAGALNLVKCVEQGVPPG-----PLLGQLK----**

**DmoTRZ1 (192) ------------------------------------------------------------------------PLQPILSYICKLKPRPGALNLVKCVEAGVPPG-----PLLGQLK----**

**DsiTRZ1 (192) ------------------------------------------------------------------------PTKSVINYICQLKPRAGALNLVKCVEQGVPPG-----PLLGQLK----**

**DseTRZ1 (192) ------------------------------------------------------------------------PTKSVINYICQLKPRAGALNLVKCVEQGVPPG-----PLLGQLK----**

**DviTRZ1 (199) ------------------------------------------------------------------------LAQPVLSYICKLKPRPGALNLVKCVEAGVPPG-----PLLGQLK----**

**DwiTRZ1 (198) ------------------------------------------------------------------------AEQVVMSYICQMKPRPGALNLVKCVEQGLKPG-----PLMGQLK----**

**DyaTRZ1 (192) ------------------------------------------------------------------------PTKSVINYICQLKPRAGALNLVKCVEQGVPPG-----PLLGQLK----**

**HmeTRZ1 (208) VAKKAKLSDDSSDREFEEFIHDNTDYYKRDGKPGFNK--KN-NTNVKPKSTVGNDGINSKDQKVLHDLK--KHNHCTVAYICILKKRLGTLDLEKCVEFGVKPG-----PMLGMLK----**

**NviTRZ1 (200) -------------EQSSRIFVDNTNYYDYQVNSNGKRSHS--------------PPSSPRQRSRSVSLEDKSRIDHTMVYICKVHPKTGTLDMAKCVDAGVTPG-----PHLGKLK----**

**TcaTRZ1 (187) SGNSSPIS-EPKNSNLGQMQEDNIDYYAHEHSGR--RK----------------SPPKISESKLLQQK--NKPTNFSMAYICQLQPRPGALLLDKCVSFGVPPG-----PLLGQLK----**

**AsuTRZ1 (251) -----------------AVNEASRM---------------------------------------DLNRPVKPIQGVDIAYLIELNSPPRRVDPTKLIELRIPNG-----PLIGRLK----**

**BmaTRZ1 (213) -----------------QRSMQEK---------------------------------------------------LDIAFLVELKPPQRSLDIQKIKKLKIPSG-----PHMKQLK----**

**CbrTRZ1 (233) -----------------TSRTSNK-----------------------------------------N-----KVNNVDVAFLIEMKEAARRIDVTKLLELKVPKG-----PLIGKLK----**

**CreTRZ1 (233) -----------------KNKEKN------------------------------------------S-----KVNNVDVAFLIEMKEAARRIDAVKLMELKVPKG-----PLIGKLK----**

**CelTRZ1 (237) -----------------GSNNEKSK---------------------------------------NV-----KVNNVDIAFLIEMKEAARRIDTMKLMELKVPKG-----PLIGKLK----**

**LloTRZ1 (212) -----------------QRSMQEKV---------------------------------------YI----LRKQYLDIAFLVELKPPQRSLDIQKIKKLKIPSG-----PHMKQLK----**

**PpaTRZ1 (271) -----------------DTSRKN---------------------------------------------------TTDVAYYIEMKDPPRRIDPTLLIKMAVPKG-----PLIGMLK----**

**TspTRZ1 (202) -------------------------------------------------CS----------------EEKTDERRPTFAYFCRIKQKSPKLLLEKCAQLNVPVG-----PLLGRLQ----**

**WbaTRZ1 (213) -----------------QRSMQEK---------------------------------------------------LDIAFLVELKPPQRSLDIQKIRNLKIPSG-----PHMKQLK----**

**CteTRZ1 (143) ------------------------------------------------------------------GKKRVLYDDEVTSYIIEALPLARRIDMEKCVLLGIKQG-----PMVGQLK----**

**LgiTRZ2 (172) --------------R--------TDEAESASK---------------------------------KPRLDSLPVEVSVAYICTPKPSERKIDILKCKELDIRPG-----PSFNLLK----**

**LgiTRZ3 (204) --------------K--------TDEAAFASK---------------------------------KPKLDSLPVEVSVAYICTPKPSIRRIDIQKCIELDIAPG-----PCLNLVK----**

**HroTRZ1 (202) -----------------------------------------------------------PVTVKKMKLSKEAVAINSVAYLCKPHAKQRSVDLAKCVELNVPAG-----PHLALLK----**

**ShaTRZ2 (204) -----------------EETPEPVAKRARRNS---------------------------------SSTSEVISQDTAFAYICKAKPPLAKINIEKCYDAGVTIG-----PMVGQLQ----**

**SmaTRZ2 (169) --------------------EEPVYKRR-------------------------------------KENLDHVSESSVFMYYFQPFRTRRKLIMSKFNEACIPASVLK-SGEIQTVI----**

**NveTRZ3 (180) -----------------DKSHDRKDENLNSEQQ---------------------------HPDKKIKLDPGLLKESISCYICQLVSVPGKFLLNKAKELGVPKG-----PLYGKLK----**

**NveTRZ2 (251) ------------------------------------------------------------------------LPEDSLCFICKLADPPGKFLAERAQELGIPVG-----HMRKELL----**

**HmaTRZ2 (147) -------------------------------------------------------------------N---KSLSQAMSYLCQLLPLPGKFLIKDALRLGVPKG-----PLFGALS----**

**AquTRZ2 (168) ------------------------------------------------------------------------VSESIAAYVIQLPTLRGKADKEKAIELGVVSVQD-----FKALL----**

**TadTRZ1 (168) SEASMNKN---KKMKVSEERFPTSHVYNYQN----------------------------------------PQQPTIISYICQLSQLPGKFLNDKAEALGVPNN-----KLRKSLI----**

**MbrTRZ2 (178) -----------------------------------------------------------------ADPSFEGSADRVLSFVGHFCDKPGTLDVEKAKALGVPPG-----RLFGQLK----**

******* * * ****

**BtaTRZ2 (273) ----------DGKSVTYE--GREILPEEICTPPDPGIT----FIVVECP---------DEGFIQPLCE-NTTFRSYQG-KAD-------APVALVVHMAPEHVLLDSRYQQWMER-----**

**CfaTRZ2 (272) ----------DGKSITYE--GREILPEEICTPPDPGLA----FIVVECP---------DEEFIQPVCE-NATLRRYQG-DAD-------APVALVVHMAPERVLADPRYQQWMER-----**

**CpoTRZ2 (276) ----------DGKNVTYE--GREILAEELCTPPDPGPA----FIVVECP---------DEGFIQPICD-NATFKRYQE-ETN-------DPVAVVVHMAPESVLSDSRYQQWMER-----**

**CjaTRZ2 (280) ----------DGKSITHE--GREILPEELCTPPDPGAA----FVVVECP---------DEGFIQPICE-NATFQRYQG-NAD-------APVALVVHLAPESVLVDSRYQQWMER-----**

**EcaTRZ2 (271) ----------DGKSVTYE--GREILPEEICTPPDPGLA----FFVVECP---------DEGFIQPICE-NATFKRYQG-KAD-------APVALVVHMAPECVLMDSRYQQWMER-----**

**LafTRZ2 (274) ----------DGKSVTFE--GREILPEEICTPPDPGLA----FVVVECP---------DEGFIQPICE-NAAFQRYQG-KAD-------APVALVVHLAPESVLLDSRYQQWMER-----**

**LgoTRZ2 (280) ----------DGKSITHE--GREILAEELCTPPDPGAA----FVVVECP---------DESFIQPICE-NATFQRYQG-KAD-------APVALVVHMAPESVLVDSRYQQWMER-----**

**HsaTRZ2 (280) ----------DGKSITHE--GREILAEELCTPPDPGAA----FVVVECP---------DESFIQPICE-NATFQRYQG-KAD-------APVALVVHMAPASVLVDSRYQQWMER-----**

**MmuTRZ2 (276) ----------DGKSITYE--GREIAAEELCTPPDPGLV----FIVVECP---------DEGFILPICE-NDTFKRYQA-EAD-------APVALVVHIAPESVLIDSRYQQWMER-----**

**PtrTRZ2 (280) ----------DGKSITHE--GREILAEELCTPPDPGAA----FVVVECP---------DESFIQPICE-NATFQRYQG-KAD-------APVALVVHMAPESVLVDSRYQQWMER-----**

**OcuTRZ2 (276) ----------NGESITFE--GREILPEELCTPPDPGLV----FVVVECP---------DEGFIQPICE-NATLQRYQG-KAD-------APVALVVHIAPESVLMDSRYQQWMER-----**

**RnoTRZ2 (279) ----------DGKSITYE--GREIAAEELCTPPDPGLV----FIVVECP---------DEGFIQPICE-NDTFQRYQG-EAD-------APVAVVVHIAPESVLIDSRYQQWMER-----**

**SscTRZ2 (274) ----------DGKSVTYE--GREILPEEICTPPDPGVA----FVVVECP---------DEGFIQPVCE-NATFRSYQG-NAE-------APVALVVHMAPESVLLDSRYQQWMER-----**

**GgaTRZ2 (290) ----------NGESITFE--GKELSPEELCAPGDPGPV----FIVLECP---------HEGFVDAVCE-NETFRRYQGGLNE-------DKVALVIHMTPESVLRDSRYQQWLER-----**

**AcaTRZ2 (282) ----------DGKNITFE--GKEILAEEICTPTDPGAV----FIIVECP---------HEGFVDAVCE-NDTLKGYQEGKQE-------NPVALVIHITPEPILRDSRYKQWLER-----**

**XtrTRZ2 (290) ----------SGKSVTYQ--GREIFPEDVCTPADPGPI----FIVVECP---------TEEFVMPVVE-NETFKRYQEKKSG-------SPVALVIHMTPESILHSSRYWHWMKQ-----**

**DreTRZ2 (351) ----------SGKTVTHE--GKEIRPEELCTPADPGPV----FIVVDCP---------SEDFIKPLCS-NPILKRFQSGGSE-------DSAALVVHMTPEAVLNTQEYKSWMER-----**

**GacTRZ2 (317) ----------SGKSVEYE--GKEIRPEQVCTPTDPGPV----FLIVECP---------SEEFVEAVCT-NAQLRRHQAAAGE-------ESAALVVHMAPESVLSTDQYKRWMQR-----**

**OlaTRZ2 (319) ----------SGQSVIHD--GKEIRPEQVCTPTDPGPV----FMVVECP---------SEEFLEAVCS-NQQLRRYQTGGTE-------DSAVLVVHMTPERLLETDQYKKWMER-----**

**SsaTRZ2 (321) ----------DGKSVTYE--GREIHPEEVCTPTDPGPA----FIVVECP---------SEEFVQPICI-HQQLSRYQRGGTE-------DPAALVVHMSPESVLKTDEYKQWMER-----**

**TruTRZ2 (305) ----------NGRSITYE--GREIRPEQVCTPTDPGPV----FIIVECP---------SEAFVEAVCG-EQRLSRYQTGGSE-------DAPALVVHVAPERVLNTDQYKTWMER-----**

**TniTRZ2 (284) ----------DGRSVTYE--GREIRPEQVCTPPDPGPA----FLVVECP---------SEAFVEAVCS-AQPLRRYQTGGSE-------DAAALVVHMAPERVLSTDGYRAWMER-----**

**CinTRZ2 (217) LIRKFQSLFFKKKISFHPFTSEVIDPTDYYYDGDVIYSR---VAVVECP---------TLKYFQQVVR-NKELAKYYSETS---------MLCEMAHITPQWLFISPQYQAWMKR-----**

**CsaTRZ2 (273) AFKAFKSELIKGNVAINPETGETINPEDLKYEGPGFQEV---YGVVECP---------SLDYLPALVE-NSEFSKYFAANES--------KMNKIAHITPQDVFDSGSYQSWIQK-----**

**BflTRZ2 (266) ----------KGNQATLPD-GRIINPEDYIEPDTPGPV----FIVVECP---------MENFIDPVTT-NSQIAKYQEDGDE-------ERPAIVVHFTPAGVVKNTQYQEWIRR-----**

**SpuTRZ2 (398) ----------SGKSVTLDD-GTVVKAEDCLGPVIPGQV----FIVLECP---------SVDYIEPIVSNEVFTRHFSDCGED--------AASLVLHMCPEEVLHDERYQQWITR-----**

**SkoTRZ2 (226) ---------RGKTVLTPD--GKEVHPADVLTPVVIGPV----FIVLECP---------TEDYLDSLES-STKLNEYLKDDVEQ------P-VSLVVHLTPPEILSTSRYQAWMKR-----**

**AaeTRZ1 (282) ----------NGNDVTLPD-GTVVKSSDVRGPDDPGPV----FIFIDIP---------SEEYLRDLDDKNEAFAPYQSTATE-----ESNQAMYVLHFSPLDVMQKPEYRKFMDR-----**

**AdaTRZ1 (285) ----------NGHDVTLPD-GRVVRSCDVRAPDDPGPV----FIFLDIP---------SEEYFKDFQQKANVFAPYQQGATE-----NSDHAMFVIHFSPLNVMRCPEYKQFMDR-----**

**AecTRZ1 (299) ----------TGADITLPD-GTIVLSKDVCSPAKPGPT----FIIVECP---------SEDYLENFIN-HPAFVRHQKGTAN-----KDDIPYCIIHFTPQEVMDNSRYVNWMSK-----**

**AgaTRZ1 (289) ----------NGNDVTLPD-GRVVRSCEVRAPDDPGPV----FMFIDIP---------SREYMDDFVAKAELFEKYQQTAAE-----ESDQAIFVVHFSPLDVMRCDEYRQFMDR-----**

**ApiTRZ1 (279) ----------SGSDITLSD-GTLVKSSDVTSPDDPGPV----FIVVECP---------SEDYIDSLLN-ESIFSKHQLGVVN-----EEDIAFMVVHFTPLDVRNDPRYQSWMKL-----**

**BmoTRZ1 (280) ----------NGEDVVLPD-GTLVLSKDVKTPDDPGPV----FIVMEVP------------EISYLKE--SEFSAHFDDGKNP----VENVPAIIVHYSPPNVFNHPTYRAFLSK-----**

**CflTRZ1 (255) ----------AGKDITLPD-GTVVLSKDVCSPTTPGPV----FLIVECP---------LEDYLESFVN-HPAFVQHQATATMSN---ENDRPYCIIHFTPQRVIDDPRYIDWMNK-----**

**CpiTRZ1 (251) ----------NGNDVTLPD-GKVVKSSDVRGPNDPGPV----FIFIDIP---------SEAYLSDLEAKNEAFVPYQSTATD-----ESDQAMYVVHFTPLDVMRTERYRTFLNK-----**

**CquTRZ1 (251) ----------NGNDVTLPD-GKVVKSSDVRGPNDPGPV----FIFIDIP---------SEAYLSDLEAKNEAFVPYQSTATD-----ESDQAMYVVHFTPLDVMRTERYRTFLNK-----**

**DpuTRZ1 (208) ----------AGQDITLPN-GKTVLASDVCSPDDPGPV----FVVVECP---------DETYLDNFVS-EPQLCQLQR-RNGAS---ELDSPQIIVHFTPLELTKHPKYQEWMDG-----**

**DanTRZ1 (231) ----------NGQDITLPD-GTVVRSADVTEASETHLS----FVFLDVP---------TEDYLPGLLA-QASRIKELGSQKL-------TEVALVIHFTPERITSLQDYKDYVAQN----**

**DerTRZ1 (231) ----------NGKDITLPD-GKVVRSADVTEASETALS----FVFLDVP---------SEKYLPGLLT-HGKRLKKLGEEKL-------TEVAVVVHFTPYHMSSRQEYKDFVVDN----**

**DgrTRZ1 (239) ----------NGHDVVLPC-GKVVHSVDVTEPGETALS----FVFIDVP---------SEDYLQSLQA-QAQEYKKLAATQL-------TEVAVVVHFTPATLTAHPDYRRFMEQN----**

**DmeTRZ1 (231) ----------NGNDITLPD-GKVVRSVDVTEASETALS----FVFLDVP---------SENYLPALLT-HGKRLKKLGEEKL-------TEVALVVHFTPYHISSRQVYKDFVVES----**

**DmoTRZ1 (231) ----------NGQDVTLPC-GTVVHSVDVTEPAETALS----FVFIDVP---------SLDYLESLKA-QSEQYKQLANSEL-------TEVALVVHFSPPELTAHAEYLDFMQTN----**

**DsiTRZ1 (231) ----------NGNDITLPD-GKVVRSLDVTEASETALS----FVFLDVP---------SENYLPALLT-HGTRLKKLGEEKL-------TEVALVVHFTPYHMSSRQEYKDFVVEN----**

**DseTRZ1 (231) ----------NGNDITLPD-GKVVRSVDVTEASETALS----FVFLDVP---------CENYLPALLT-HGKRLKKLGEEKL-------TEVALVVHFTPYHISSRQEYKDFVVEN----**

**DviTRZ1 (238) ----------NGQDVTLPC-GKVVYSVDVTEPGETALS----FVFIDVP---------SEQYLSSLQA-QAEQYKQLAASEL-------TEVALVVHFSPPELVVNAHYSSFMAAN----**

**DwiTRZ1 (237) ----------NGQDVTLPD-GRVVRSADVTEPAETALS----FVFVDLP---------DRDFLPSLKE-QANIFKDLGKTKL-------GVVALVIHFTPHEVSSLPEYQTFLTDN----**

**DyaTRZ1 (231) ----------NGKDITLPD-GKVVRSTDVTEASETALS----FVLLDVP---------SMNYLPGLLT-HGKRLKKLGEEKL-------TEVAVVVHFTPYHMSSRQEYKDFVGDN----**

**HmeTRZ1 (314) ----------NGQDVVLPD-GTLVQSKNVKTPDDPGPV----FIVMEVP------------DLSYLKE--LEFSAHFDNGANP----PENIPTVIVHYTPPHVFNHPTYRAFLAM-----**

**NviTRZ1 (284) ----------AGEDITLPD-GRVIKSKDVVSPSQIGPL----FIVLECP---------DEDWIDVIVE-NPAFLKYQEGTAK-I---EEEIARYIVHFSTKNVLENPKYKDWMRK-----**

**TcaTRZ1 (277) ----------SGHDVTLVN-GKVVKASDVCEPDDPGPV----FIVLECP---------SEEYLDSLID-CEEFKKHQETATK-----DEDLAHTVVHFTPQKIINHPRYKEWVDK-----**

**AsuTRZ1 (306) ----------NGESVTLED-GRLIKPDDILFSEIAEEQPN--ALIVECV---------DFEAVESIKN-NSLLQKFIDGTEK---------MNFVVHFTANEILNSNEYTAWMTSF----**

**BmaTRZ1 (256) ----------DGENITLSD-GRFVKAEEVLSDLKAKPLYA--CLIVECS---------DLKKLQSLQN-NTLLRGYNNRDRS---------LRYVVHFTKNSVLNNDEYKSWMASF----**

**CbrTRZ1 (281) ----------SGEAVTLPD-GRTIQPDQVFSSHKVEGEKPF-LLVAECT---------TEQHVQSLLD-SSSMQQFFNKGKQ---------LDYMVHLSRESVINTESYKKLMEKL----**

**CreTRZ1 (280) ----------SGEAVTLPD-GRTIQPDQVFSSHKVEGEKPI-LLVAECT---------TEEHVESLLN-SSSMQPFFNNEKR---------LDYMVHLSKEELINTPSYKTLMNKL----**

**CelTRZ1 (287) ----------SGEAVTLPD-GRTIQPDQVFSSDKVEGDKPL-LLVTECT---------TEDHVKALID-SSSLQPFLNGEKQ---------LDYMVHISDDAVINTPTYRHLMEKL----**

**LloTRZ1 (263) ----------DGENITLSD-GRFIKAEEVLSDLKAKPQYA--CLIVECS---------DLRKLPSLQN-NTLLRDYNIRNKT---------LRYVVHFTENSVLINDEYKSWMASF----**

**PpaTRZ1 (314) ----------NGETITLPN-GNLVKPDDVMMDDDRAGSEKRRLLIVDAA---------DEGYARSLYESSIIRSLASAAASN--------KLDFVVHLTRENVLRTKEYEQWAESL----**

**TspTRZ1 (248) ----------AGCDVQLDD-GTTVRSADVLEPATPCPT----FVVVECP----------RGKEPPKRS---TIERLLRQGE---------TLDAVVHLTDAQVYETDFYQRWMVE-----**

**WbaTRZ1 (256) ----------DGEDITLSD-GRFVKAEEVLSDLKAKPLYA--CLIVECS---------DLRKLQSLQN-NTLLRGYNNRDRC---------LRYVVHFTENSVLNNDEYRSWMASF----**

**CteTRZ1 (188) ----------KGEAVTLPD-GTVIEPEQVLRDAEPVNPP---LLVVDCP---------SDCYITSLNNCEDLQPFYADKKLS-----------LVVHLTPGDVMLDAAYIHWMKR-----**

**LgiTRZ2 (228) ----------TGESVTLED-GRVINSEDILKPLVPS--PS----FINCP---------HSGFLTPLIN-NEGLKKYQNTDENTP---VEMTVDLIIHLTPEHILKLPEYQQWMNS-----**

**LgiTRZ3 (260) ----------TGESVTLED-GRVINSEDILKPLVQS--PS----FIKCS---------HSGFLTPLIN-NEGLKKYQNTDENTP---VEMTVDLIIHLTPEHIVKLPEYQQWINS-----**

**HroTRZ1 (254) ----------EGHEVILPGTDRLIKPDDVLSKCEPNHC----FLVVECSSLGHVRCVTSSPILSPYMNHDEKTSLSTLSPPPPPQSSSLSPLNLIIHFTHSSIMSHPLYQTWMKSFR---**

**ShaTRZ2 (265) ----------RGFSVTLED-GRIVYPSQVIDDIAVDRRP---FLVVECP---------HESFLTSLMT-SPQLQEFMTSSDP------QSSFAVIVHMTPSEVYNSDEYQKWMKR-----**

**SmaTRZ2 (227) ----------NGENLILSD-GRIISPDEVTAPSPPSKN----MLFIDCP---------NSDYIPAFISNEEFFNSIQAEESNEN---LTSGVSLVVHFTPPGMFYSNQYQKFVQKLEECA**

**NveTRZ3 (247) ----------NGENIILED-GTEVCPEQVTEPTSPGPV----VLFIDCP---------CVDYIPNIVNNHSLCAFHEGPSN--------MAPVIIVHMVPMAVYNHPDYVSWSKK-----**

**NveTRZ2 (290) ----------SGNPVVLPD-GRTVKPDEVVVPPKPGQI----FAVIDCS---------TAEKLQLLTN-HKYFQDHIDCKQ----------LDLMIHMTPMSLFQTNTYQSWVRE-----**

**HmaTRZ2 (188) ----------RGESVTLPC-GKVISPEQVMEPQVNGVT----FFVLDCP---------TELHIKSLQG----IKQYMKLKSS---------PHLVIHAASKSVCNSDFYKLFLQWYI---**

**AquTRZ2 (207) ----------NGVSVTSIN-GNAVYPHQVLEPEQVGPQ----ALVLECP---------DPGFLKDITTHPFLNAPQLK-----------P--VVVVHITPKAVLETTLYNQWMKQ-----**

**TadTRZ1 (236) ----------MGQTITLRN-GTVVRPEDCVGPQEPGMV----FMIIHCP---------TLDHIPQLVGCNRFESYFTNGENQ-------Q-PELMLHICPMNVIENTAYLDWTKK-----**

**MbrTRZ2 (224) ----------RGEAVQVPD-GRIVQPDEVLSPPLCPPA----FAILDVS---------PAETLDSLRPVAEHFASLDRDG-------------TVYHMAAWATVHTPEYADLLNA-----**

*** ***

**BtaTRZ2 (354) --------FGPDTEHLVLN------------------------------------------------------ETCE-SVHNLRSHKIQTQLSLIHPGIFPPLAGP--------------**

**CfaTRZ2 (353) --------FGPATQHLVLN------------------------------------------------------ESCS-SVHNLRSHKIQTQLGLIHSDIFPPLATP--------------**

**CpoTRZ2 (357) --------FGPDTQHLILN------------------------------------------------------ENCA-SVHNLRSHKIQTQLNLIQPDIFPLLTSF--------------**

**CjaTRZ2 (361) --------FGPDTQHLVLN------------------------------------------------------ENCS-SVHCVRSHKIQTQLNLIHPDIFPLLTSF--------------**

**EcaTRZ2 (352) --------FGPDTQHLILN------------------------------------------------------EKSA-SVHNLRSHKIQTQLNLIHPDIFPPLASV--------------**

**LafTRZ2 (355) --------FGPDTQHLILN------------------------------------------------------ENCT-SVHNLRSHKIQTQLNLIHPSIFPPLADL--------------**

**LgoTRZ2 (361) --------FGPDTQHLVLN------------------------------------------------------ENCA-SVHNLRSHKIQTQLNLIHPDIFPLLTSF--------------**

**HsaTRZ2 (361) --------FGPDTQHLVLN------------------------------------------------------ENCA-SVHNLRSHKIQTQLNLIHPDIFPLLTSF--------------**

**MmuTRZ2 (357) --------FGPDTQHLILN------------------------------------------------------ENCP-SVHNLRSHKIQTQLSLIHPDIFPQLTSF--------------**

**PtrTRZ2 (361) --------FGPDTQHLVLN------------------------------------------------------ENCA-SVHNLRSHKIQTQLNLIHPDIFPLLTSF--------------**

**OcuTRZ2 (357) --------FGPDTQHLILN------------------------------------------------------ENCT-SLHNLRSHKIQTQLNLIHPGIFPPLASL--------------**

**RnoTRZ2 (360) --------FGPDTQHLILN------------------------------------------------------ENCP-SVHNLRSHKIQTQLSLIHPDIFPQLTSF--------------**

**SscTRZ2 (355) --------FGPDTQHLVLN------------------------------------------------------EHCE-SVHNLRSHKIQTQLNLIHSGIFPPLASL--------------**

**GgaTRZ2 (372) --------FGPGTQHLVLN------------------------------------------------------ENCS-AVHNARSYKIQSQLNLIHPEIFPLLTTY--------------**

**AcaTRZ2 (364) --------FGSTTQHLILN------------------------------------------------------ENCK-SIHHLRSHKIQTQLNLIHSEIFPLLTNY--------------**

**XtrTRZ2 (372) --------FGPQTEHLILN------------------------------------------------------ENTS-TLHNLRSYKIQTQLNLVHPEIFPQLANV--------------**

**DreTRZ2 (433) --------FPSSTEHLLMN------------------------------------------------------EQTF-TPHNARSHKLQTQLNLIHPHIFPQLKLY--------------**

**GacTRZ2 (399) --------FPPTTQHLILN------------------------------------------------------EQFG-AVHNVRSHKIQAQLNMIHGHIFPELKSY--------------**

**OlaTRZ2 (401) --------FPSTTEHLIMN------------------------------------------------------EHAC-TVHNIRSHKIQTQLNMIHPQIFPELKAY--------------**

**SsaTRZ2 (403) --------FPSTTEHLILN------------------------------------------------------EQVY-TVHNVRSHKIQTQLNLIHPEIFPELQHY--------------**

**TruTRZ2 (387) --------FPSRTEHLILN------------------------------------------------------EHVC-TAHNVRSHKIQAQLNMIHSEIFPQLHTC--------------**

**TniTRZ2 (366) --------FPASTEHLILN------------------------------------------------------EHVT-AVHNVRSHKIQTQLNLIHPEIFPPLQTW--------------**

**CinTRZ2 (310) --------FGESCVHIPVN------------------------------------------------------SDLNETPQISLPGVARDALNIVSETFFPVYTN---------------**

**CsaTRZ2 (367) --------FGDNCRHVVAN------------------------------------------------------ESYNLKSAITLPDTVREAQNCVSDQFFPFYQGHP-------------**

**BflTRZ2 (349) --------FGPDTEHLFLN------------------------------------------------------EDTQ-TTSHKGTASLQACLHTIQPTVFPLLADN--------------**

**SpuTRZ2 (481) --------FGATAEHIIFN------------------------------------------------------ESCE-SLRLDASRGQQTLLNQLHEGIFPILPTHT-------------**

**SkoTRZ2 (309) --------FGEKTDHLVMN------------------------------------------------------SESE-SHGLNKSWLLQNKLNMICPQIFPDLKG---------------**

**AaeTRZ1 (368) --------FSASTRHIALN------------------------------------------------------EINS-FSGYIASHRIQYHLNQLDGGIFPLLKE---------------**

**AdaTRZ1 (371) --------FSASTRHIALN------------------------------------------------------EVNS-FSGYVAAHRIQYHLNQLDEQIFPILKE---------------**

**AecTRZ1 (384) --------FGLNTRHIVVN------------------------------------------------------EENQ-CMGTEAMHRHQHKLHMLHPEIFPFLNE---------------**

**AgaTRZ1 (375) --------FSASTRHIALN------------------------------------------------------EVNS-FSGYIAAHRIQYHLNQLDEQIFPLLRE---------------**

**ApiTRZ1 (364) --------FPSETYHLLLN------------------------------------------------------NENK-CLGSTAIHRIQYKLNMLDENIYPLLKDN-GIPIIDQQEK---**

**BmoTRZ1 (362) --------FGPTTRHLILN------------------------------------------------------TQNS-CLGSEAVHRTQHKLHLLDPDIFPLLKDV-SVPAVWNAQPPVP**

**CflTRZ1 (342) --------FDSNTRHIVVN------------------------------------------------------EDNE-CMGTEAIHRHQHKLHMLHSEIFPFLNE---------------**

**CpiTRZ1 (337) --------FSASTRHIALN------------------------------------------------------EINK-FSGYIASHRIQYHLNQLDKDIFPVLKE---------------**

**CquTRZ1 (337) --------FSASTRHIALN------------------------------------------------------EINK-FSGYIASHRIQYHLNQLDKDIFPVLKE---------------**

**DpuTRZ1 (294) --------FSVTTCHLMLG------------------------------------------------------TRG---FGSVAVHRIQHQLHLLDSEIFPHLP-F-DMR----------**

**DanTRZ1 (315) --------FSKDTQHIYLGS-----------------------------------------------------PNNQ-FSGYAAAHRIQHQLHQLAPQVFPLLSE-----QSPCQSQNLS**

**DerTRZ1 (315) --------FSPETQHIYLSS-----------------------------------------------------PLNQ-FSGYAAAHRIQHQLHQLAPQVFPLLGE-----QLSCQSQTLS**

**DgrTRZ1 (323) --------FSQCTQHIYLNS-----------------------------------------------------PKNP-FSGYAAAHRIQYQLHQLEPNIFPLLAES-DELQRSCPSSMLS**

**DmeTRZ1 (315) --------FSSEAQHIYLSS-----------------------------------------------------PLNQ-FSGYAAAHRIQHQLHQLAPQVFPLLGE-----QLSCQSQTLS**

**DmoTRZ1 (315) --------FSAGTQHIYLNS-----------------------------------------------------HLNR-FSGYAAAHRIQYQLHQLAPRFFPLLAEA-AELQLSCPSNTLS**

**DsiTRZ1 (315) --------FSPEAQHIYLSS-----------------------------------------------------PLNQ-FSGYAAAHRIQHQLHQLAPQVFPLLCE-----QLSCQSQTLS**

**DseTRZ1 (315) --------FSPETQHIYLSS-----------------------------------------------------PLNQ-FSGYAAAHRIQHQLHQLAPQVFPLLCE-----QLSCQSQTLS**

**DviTRZ1 (322) --------FSPGTQHIYLNS-----------------------------------------------------PQNP-FSGYAAAHRIQYQLHQLAPRIFPLLAES-TDLQHSWQSTKLS**

**DwiTRZ1 (321) --------FSQDTQNIYLNS-----------------------------------------------------PQNG-FSGYAAAHRIQHQLNQLNPQIFPLLQE-----QVPCP---LS**

**DyaTRZ1 (315) --------FSAETQHIYLSS-----------------------------------------------------PLNQ-FSGYAAAHRIQHQLHQLAPQVFPLLGE-----QLSCQSQTLS**

**HmeTRZ1 (396) --------FGPTTRHLVLN------------------------------------------------------RQNS-CLGSESVHRTQHKLHLLDGEMFPLLRDT-SMPALWDTPTVED**

**NviTRZ1 (370) --------FGSKTQHLIIN------------------------------------------------------EENK-GYCSEAMHKMQHQLHLIHPDIFPFLGENKNFAEHNPDADPLK**

**TcaTRZ1 (362) --------FSPSTHHLILN------------------------------------------------------DSNT-CMGSSAVHRIQYKLNLLSDDIFPLLGDK-GTQTIDDQVTEPP**

**AsuTRZ1 (390) --------G-PQCMHIVLNG-----------------------------------------------------SGPI-LPHIESIYRNHALLNHLSPNLFPPLLGS--------------**

**BmaTRZ1 (340) --------G-HSVEHIIVNG-----------------------------------------------------SGPC-LPHMEAVYRINVILNHICPKLFPLLYPK--------------**

**CbrTRZ1 (366) --------SDAGTSHLLINE-----------------------------------------------------VNPV-IPAVESVYKHTRLLRTISPSLFPPLHPI--------------**

**CreTRZ1 (365) --------SDGDTTHLLINE-----------------------------------------------------ANPV-IPAVESVYKHTRLLRSISPSLFPALHPI--------------**

**CelTRZ1 (372) --------NNPSITHLLING-----------------------------------------------------GNPV-IPAVESVYKHTRLLRSIAPSLFPALHPI--------------**

**LloTRZ1 (347) --------G-QSVEHIIVNG-----------------------------------------------------TGPC-LPHMEAVYRINVILNHICPKLFPLLHPK--------------**

**PpaTRZ1 (402) --------G-TQCTHIVVNG-----------------------------------------------------AGPI-VPHIDSMYKHARLLHELQPALFDELRPR--------------**

**TspTRZ1 (326) --------LDGKTCHVVLN-----------------------------------------------------EAVAPVRPHSEAIYRFSVQLNRVHSTIFPLLCTH--------------**

**WbaTRZ1 (340) --------G-DRVEHIIVNG-----------------------------------------------------SGPC-LPHMEAVYRINVILNHICPKLFPPLYPK--------------**

**CteTRZ1 (269) --------FGADVQHLVVN------------------------------------------------------ELGE-SSPVSSVHWFQSIMNLIDSKLFPLLHDP--------------**

**LgiTRZ2 (313) --------FGANVKHMIFED-----------------------------------------------------TFSN-AMHDD-GSKIQSHLNLLHPELFPLLPALT-------------**

**LgiTRZ3 (345) --------FGANVKHMIFED-----------------------------------------------------TFSN-VMDDE-GSKIQSHLNLLHPELFPLLPALT-------------**

**HroTRZ1 (357) --------NSENIHHMVLNEKSIDNFLSTTAASNVTTNTTTEDATTILLAAVAPVATANATAEEKFQYGMFPTSTTLTTLDTEAMFKLQTYFHLIHPFIFPSLPYQSLR-----------**

**ShaTRZ2 (350) --------FPSSIDHLVMN------------------------------------------------------RDAA-EVDLLRVREHQARLSLVGSDIFPLMPELTKMDDIDGVQALTE**

**SmaTRZ2 (320) LRKSDTEDPTTTLKHLVLDG------------------------------------------------------TGYVTDRVGMYSQTFILNRFFDSKVYPLLFDMADSDTVS-------**

**NveTRZ3 (330) --------FGERTEHLLINS-----------------------------------------------------EVCSDHVAFRRQATIQCKLNTLDPSIFPLLYQS--------------**

**NveTRZ2 (370) --------FGPRTQHILLN-----------------------------------------------------TDTPACQRQLQLNNRLQRRLHSIQPTIFSAPHMEHP------------**

**HmaTRZ2 (268) --------FPPSTQHLYLS-----------------------------------------------------ECQQRLSSVFGPQHEMQMKLHQIHPIFFPLIELQ--------------**

**AquTRZ2 (285) ---------FHQCQHILLHP-----------------------------------------------------DFCPSEIAIKPGLYSSMLLHLVEPNIFNLPTTVS-------------**

**TadTRZ1 (319) --------FGPNTKHLVLNS-----------------------------------------------------DVCG-GVTPIISEESYHKLHALSSEIFPLLKYN--------------**

**MbrTRZ2 (302) --------FGPRARHVVMDP------------------------------------------------------EQDRRITHLANATNQCILNTILPPVFPRPYGVEQLDVPTLPTEFSP**

**BtaTRZ2 (397) ------------RPQEGS--------------------------------ATQGVPTVRGQCLLKYQLRPRRE---WQRDAVLTCDPEEFIAEALEL-------PNFQESVQEYRKTAQD**

**CfaTRZ2 (396) ------------HRQEEH--------------------------------TTFRVPTVRGECLLKYQLRPRRE---WQRDAVIVCNPDEFIAEALEL-------PNFQESVQEYRKAMQD**

**CpoTRZ2 (400) ------------HRKEEG--------------------------------LALGVPTVRGECLLKYQLRPRRE---WQRDAILTCNPDEFIAEALEL-------PNFQESVQEYKKSVQD**

**CjaTRZ2 (404) ------------PSKVES--------------------------------PILSVPVVRGECLLKYQLRPRRE---WQRDAIITCNPEEFIAEALQL-------PNFKESVQEYRRGAQD**

**EcaTRZ2 (395) ------------PCKEEG--------------------------------ATFSVPTVRGECLLKYQLRPRRE---WQRDAIVTCNPDEFIAEALEL-------PNFQESVQEYRKTVQE**

**LafTRZ2 (398) ------------RHQGEG--------------------------------TAFRVPAMRAECLLKYQLRPRRE---WQRDAVLTCNPDEFIAEALEL-------PNFQESVQESRKSVQD**

**LgoTRZ2 (404) ------------PCKKEG--------------------------------PTLSVPMVQGECLLKYQLRPRRE---WQRDAIITCNPEEFIVEALQL-------PNFQQSVQEYRRSVQD**

**HsaTRZ2 (404) ------------RCKKEG--------------------------------PTLSVPMVQGECLLKYQLRPRRE---WQRDAIITCNPEEFIVEALQL-------PNFQQSVQEYRRSAQD**

**MmuTRZ2 (400) ------------YSKEEG--------------------------------STLSVPTVRGECLLKYQLRPKRE---WQRDTTLDCNTDEFIAEALEL-------PSFQESVEEYRKNVQE**

**PtrTRZ2 (404) ------------PCKKEG--------------------------------PTLSVPMVQGECLLKYQLRPRRE---WQRDAIITCNPEEFIIEALQL-------PNFQQSVQEYRRSAQD**

**OcuTRZ2 (400) ------------PSQEEG--------------------------------PALRVPAVRGECLLKYQLRPRRE---WQRDAIVACNPDEFIAEALEL-------PNFQERVQEYRKSVHD**

**RnoTRZ2 (403) ------------HSKEEG--------------------------------STFSLPTVRGECLLKYHVRPKRE---WQRDTTLDCNTDEFIAEALEL-------PNFQESVEEYRKNMQA**

**SscTRZ2 (398) ------------RPQEGG--------------------------------ATFQVPTVRGQCLLKYQLRPRRE---WQRDAVVTCNPEEFIAEAMEL-------PGFRERLQEYRATAQD**

**GgaTRZ2 (415) ------------QTKEEE--------------------------------AVCSVPIVRGECLLKYHFRPHQE---WQRDAVTVCDHDAFVAEALDL-------PNFQARVKECKESLPA**

**AcaTRZ2 (407) ------------QSKEEE--------------------------------ATFSVPVTRGECLLKYQLRPKAE---WQREAVVACNSAEFVAEALEL-------PDFQDSVQKCKESLPV**

**XtrTRZ2 (415) ------------QKKE----------------------------------ERPEFFGVRGECLLKYQLRPKLE---WQRDAVTSNNTEEFVKEAMEL-------PGFVEALENCKLTLKL**

**DreTRZ2 (476) ------------RTKDPP--------------------------------ASLHVPNVRAECLLKFQLRPKLE---WQRDAIPSCDSEEFVKEAAKV-------PNFLAEVEECRKFQAA**

**GacTRZ2 (442) ------------RSKEAA--------------------------------AELLVPGVRAECLLKFQLRPVME---WQRDAVPSCCAGDFVKEASEV-------PDFLEEVEQCRKVCSA**

**OlaTRZ2 (444) ------------RAKEPQ--------------------------------AALHVPNIRAECLLKFQLRPVME---WQRDAIPACSSEEFVKEASEV-------PNFLAEVEKCRTASST**

**SsaTRZ2 (446) ------------KTKETQ--------------------------------AALHVPNVRAECLLKFQLRPKIE---WQRDAIPSCDTEEFVKEAAEA-------PNFLQEVEECRRFRAT**

**TruTRZ2 (430) ------------KATEPQ--------------------------------ASLPVPNVRAECLLKFQFRPVLE---WQRDAIPACSTDDFLKEAAEV-------PNFLQEVEACRKLRLS**

**TniTRZ2 (409) ------------TAPEPP--------------------------------ARLPVPSVRAECLLKFQFRPALE---WQRESVPVCNPDDFVKEASEV-------PNFQQEVEACRKLRLS**

**CinTRZ2 (353) ----------------------------------------------------------HSSQVEMFQCLDADDKNLFVPEVAKIEQMVERIATIPTN-----------ESQRTRVKLVDF**

**CsaTRZ2 (412) -----------------------------------------------YSCLEQAKQLFKQDDFNEHSFKNPLR----NSELMKVKKVLDVIPHCPSN-----------TSHRDCLKYAEL**

**BflTRZ2 (392) -------------AIKPNS------------------------------LPPLPHKHVRGECLLTYWLFHRDHSLQWDRSSIPLLDNEEAVKGAFAL-------PGFEDSLREMKETISA**

**SpuTRZ2 (525) -----LK----ATPIPPQIKING---------------------------SESIAKVTYAEIYNKLHLKPAKG---WERDFIISEDRQRFLDELGPLK-------GFQESLKTLKATLEE**

**SkoTRZ2 (351) ------Q----YTDIQPSE------------------------------MKVDNGLITKAECLMKYHLRPHSR---WDRSGIPTVTREQIEKDVHMIP-------GFTEKLKDLRKTLSH**

**AaeTRZ1 (410) -----A-------NNDYNAIDSE----------------------------MHDFDMVKTSSLSYLHVRPKKG---IDRTLEAKLNPSEYLQELEVL-------PEFKLALDQLHQKMAQ**

**AdaTRZ1 (413) -----E-------NNDYSQAP------------------------------AEDGHFVRSSSLSYLHIRPSKG---IERTLEASLNPSEYLGELDVL-------PDFKSTLEDWKRQQEK**

**AecTRZ1 (426) ------E----SFQKKTR--------------------------------EGDLPIIHRPKTHHTVHLQPQLR---FDTKNEVSLHPKEYVNEVFEI-------DGILDALAELQTGINA**

**AgaTRZ1 (417) -----E-------NNQYADPP------------------------------ANDTDLVRSSSLSYMHIRPPKG---IDRTLEASLNPQEYLNELELL-------PDFKEALAELKQQLAQ**

**ApiTRZ1 (417) ---------------------------------------------VIDAHPNINSLTINGQTNLSFNLRPNKY---IDRTGTLSLDVDEFIAETFKV-------DGFKEKLA--QVKQEI**

**BmoTRZ1 (418) KTGSP-------VRLKELEELKNKIAFAQFQNIINMEGSCKG-DGMLNIDDGSKLELVAGRTLTMHYLRPHKQ---VDRSAEPKLHIQDYIQETMDV-------DGFVSSLEQFRHVVEG**

**CflTRZ1 (384) ------K----SFQKKTR--------------------------------ANDLPLIHRPRTNHTIHLQPEIK---YDTMNEVSLHPEEYIKEVYEI-------EGFSEMLKDLQTNINI**

**CpiTRZ1 (379) -----D-------NNKYDELENT----------------------------ATGCDLVRSSSLSYFHIRPRKG---IDRTQEAALNPAEYLQELEAL-------PDFKLALDDLRRKIAQ**

**CquTRZ1 (379) -----D-------NNKYDELENT----------------------------ATGCDLVRSSSLSYFHIRPRKG---IDRTQEAALNPAEYLQELEAL-------PDFKLALDDLRRKIAQ**

**DpuTRZ1 (337) ---------------------------------------------VEGETEQAEAAELECQTLTKFNLRPPQK---LNTSLVPVLNPKEYVNESLAQ-------EGFPASLE---ALKSS**

**DanTRZ1 (368) QNLKKTK----LDEEDPEKEEPEKP-------------------------SNDDITEQGVVSMTSFHLRPKKG---LDRTLEAKLTPEEYVKETHAV-------PGFTELLAKFKEEYSF**

**DerTRZ1 (368) LNLKKTK----LDETDSEDKRNSK---------------------------ANETEEQGVVAMTSFHLRPRKG---LDRTLESKLTPEEYVKETHDV-------PGFLELLAKFKEEYSF**

**DgrTRZ1 (380) HSLKKTK----LNEQENLETNHETEP----------------KQMDGMVMKTETEAEQGVTSMASFHLRPKKG---LDRTLEAKLTPLEYVKETHAV-------PGFTELLAQLHNEAQL**

**DmeTRZ1 (368) LNLKKTK----LDEADSEDKANAK---------------------------ANETEEQGVVAMTNYHLRPRKG---LDRTLESKLTPEEYVKETHAV-------PGFLELLAKFKEEYSF**

**DmoTRZ1 (372) HSLKKTK----LDNDVEEQREN----------------------------KLNESESQGFVSMSSFHLRPKKG---LDRTLESKLTPEEYIKETHAV-------PGFSELLTQLQSETAE**

**DsiTRZ1 (368) INLKKTK----LDEADSEDQANAK---------------------------ANEAEEQGVVAMTNFHLRPRKG---LDRTLESKLTPEEYVKETHAV-------PGFLELLAKFKEDYSF**

**DseTRZ1 (368) INLKKTK----LDESDSEDQANAK---------------------------ANEAEEQGVVAMTNFHLRPRKG---LDRTLESKLTPEEYVKETHAV-------PGFLELLAKFKEEYSF**

**DviTRZ1 (379) HSLKKTK----LENGHGAEEPKEDS--------------------EVPIKEMKQQAEQGVVSMTSFHLRPKKG---LDRTLEAKLTPEEYINETHAV-------PCFTELLAQLQADIRD**

**DwiTRZ1 (371) QNLKKTK----LEENDQETKLEEG------------------------VTENGAGDEAGVVSMTSFHLRPRKC---LDRSLEVKLTPEEYIKETHAV-------PGFTELLDQLKSESKN**

**DyaTRZ1 (368) LNLKKTK----LDEDDSKEKTNEN---------------------------ANEAEEQGVVAMTSFHLRPRKG---LDRTLESKLTPEEYVKETHNV-------PGFMELLTKFKDEYSF**

**HmeTRZ1 (452) NQEVPSN----PVKMKVLEELK-----QNFQNIINMEGSCAGNEGLKDSDSLARLELIAGRTLTIFHLRPKKQ---LDRSVEPKLHIQEYIQEPLEV-------EGFVGSLERFRKVVED**

**NviTRZ1 (427) KIRDKLK----SIEENSEQSENEPSK------------TCETTETDVSVVQNDGLFIHQCQTLNTIHLRPNTG---LDKTSIVRINPKEYIQDVFAV-------DGFLDALADLQTQLNA**

**TcaTRZ1 (418) QPKKIKP----DSPSLPIDQLS-----------------------VKSRPASPTDPNIYTNTLFNYHLRPKKA---CDRSNELKLVPSQFIDETMAI-------ENFPSVLH--EVTQQM**

**AsuTRZ1 (433) -Q--FDG----VIGQDDECEK-------------------------------REGNIIYAHPLQRFALRGSLN----AEDPITIMVKSSDIESKVN---------QS-ETTKKAVDDFKN**

**BmaTRZ1 (383) -G--FNG----IIQQDDDCE--------------------------------KSGNCLYVRPFQRYFLRRPVN---LDLAPPNVSLVLTDLLLQLK---------EDSATSESINLFRR-**

**CbrTRZ1 (410) -N--WNG----IITQNSELAE-------------------------------KQDQFIRVAPLQRYWMRRGNS---QNEEPIINNLLASD--------------PEISVKAKELIAEYRK**

**CreTRZ1 (409) -D--WSG----IISQNEELSQ-------------------------------KENEFIRVAPLQRYWMRKGNS---LNEEPIINNLLASE--------------PELTEKAKQLVTDFQK**

**CelTRZ1 (416) -D--WSG----IITQNEELSQ-------------------------------RQDQFIRVAPMQRYWMRRGAS---FNEEPIVNNLLAAE--------------PELSDKAKELIKEYQK**

**LloTRZ1 (390) -G--FSG----TIQQDDDCE--------------------------------RSGNYLYVRPFQRYFLRRPVN---LDSSPPNVSLILTDLLLQLK---------ENSATSEAINLFMRN**

**PpaTRZ1 (445) -G--WRG----IVTQEQDLAV-------------------------------KASLWLRAAPLQRWQMRKLK-----GEEPIILDLKEAERKEAGQYNL----GEEWRDLAKEEIMIAKN**

**TspTRZ1 (371) -----DQ----ATVADTRSPLLLTTG-------------------------RQSVVAVAGEPWLRFNLRPEPG------SVYKCAPSFDRATILEPIDG----QPLVVDELRRFQERLSG**

**WbaTRZ1 (383) -G--FNG----IIQQDDDCE--------------------------------KSGNCLYVRPFQRYFLRRPVN---LDSAPPNVSLVLTDLLLQLK---------EDSATSESINLFRRG**

**CteTRZ1 (312) -----------FMKTEVDME---------------------------------GNRELLSKHGLNVTLGRSNLKYLWSRTQMFDANEVVTRLDVAASQEEALQDEDFKKMLQEFLPKVAE**

**LgiTRZ2 (357) ------------TPTSTLP--------------------------------DMTTNIIRGENNLKYHYKPYTQ---LSRELITQPNLEDFQSEITRN-------PEITEAIDNFKQEVAK**

**LgiTRZ3 (389) ------------TPTSTLP--------------------------------DMTSNIITGENNLKYHYKPYTQ---LSRELITQPNLEDFQSEITRN-------PEITEAIDNFKQEVAK**

**HroTRZ1 (458) -----KQ----QLSSENDFSIDSKIMSDN-----------------------KNNMIKYAATNAKFIMRPVSES-PFNFDQCISINPKSYVDELIER------EDGLKEFLDEWRNKNNF**

**ShaTRZ2 (407) SGVENGA----STATSASSSESE-------------------------LGSLGQGRIIKACSGLVYVYRGKRRGYYIEADEFNCQAIQKPFLEIPEVTL----ELDKLGNSSSHESQSED**

**SmaTRZ2 (379) -----------KRNETISKN------------------------------VDPFSSVVTAEPYLQFSLRPWTG---FNKPTYPQLNGDEFVSQIFDP-----IYMSLQEAEEQFVKMRES**

**NveTRZ3 (375) -----------PKK-DLP---------------------------------QFPIRTVPGAFCLQYHLRPLKQ-----QGYSTSDVPEPLNVQELKN--------EAITAIKDIKKREDN**

**NveTRZ2 (417) ---------------------------------------------LHTQWLDLPDNCVSGQSRMLYNFRPVNN----EGLFWDGIPSKPDIFWKSNS--------DLEESGSNYNEQQMV**

**HmaTRZ2 (313) ------------------------------------------------VILLILIDIKLRDVMSSIEYDNSQTISEIEINTIPVANNFKTENDFFKSAQLNKSNNIFERVSNLFRKRKSS**

**AquTRZ2 (330) ------------HKKSLS--------------------------------PAFDSPVIIGQCSMAFHIRPLTRFG--VDKNFSSKMCSSSIDSVLNDLMLDDQLIDFIEAHPFLKSNFND**

**TadTRZ1 (363) ---------------------------------------------------KLRPVIMRLLVLFNFSTYYHTILQETNVNSIVEKINNRMDIKNAIQFS----NQDILDRENRTLSVKAT**

**MbrTRZ2 (360) ASTQQMQQEGRLVASDPLRITYLRADVSDTRRLLSDKRRTRQDRGRESKRAKTEATEANSEVSTATDLADNDGADVRYSEQARQLMNREAILRGSWMPVKAAEAAALVRALPEYQKRLPT**

**PxKxRN loop Motif I**

**BtaTRZ2 (463) SP------------------------------------------EATS------SQYPEVVFLGTGSAIPMKIRNVSSTLVNISPDTS-----LLLDCGEGTFGQLCRHYG-DGVDRVLG**

**CfaTRZ2 (462) GP------------------------------------------APAEKR----SQYPEIIFLGTGSAIPMKIRNVSATLVNISPDRS-----LLLDCGEGTFGQLCRHYG-DEVDRVLG**

**CpoTRZ2 (466) AP------------------------------------------ASTEKK----NQYPEIVFLGTGSAIPMKIRNVSATLVNISPEQS-----LLLDCGEGTFGQLCRHYG-DEVDRVLG**

**CjaTRZ2 (470) GP------------------------------------------VPAEK-----SPYPEIVFLGTGSAIPMKIRNVSATLVNISPDTS-----LLLDCGEGTFGQLCRHYG-DQVDRVLG**

**EcaTRZ2 (461) GP------------------------------------------APADTR----SQYPEIIFLGTGSAIPMKIRNVSSTLVNISSDKS-----LLLDCGEGTFGQLCRHYG-DDVDRVLG**

**LafTRZ2 (464) GP------------------------------------------TPAEKS----SQYPEIVFLGTGSAIPMKIRNVSSTLVNISSDRS-----LLLDCGEGTLGQLCRHYG-DEVDRVLG**

**LgoTRZ2 (470) VP------------------------------------------APAEKR----SQYPEIIFLGTGSAIPMKIRNVSATLVNISPDTS-----LLLDCGEGTFGQLCRHYG-DQVDRVLG**

**HsaTRZ2 (470) GP------------------------------------------APAEKR----SQYPEIIFLGTGSAIPMKIRNVSATLVNISPDTS-----LLLDCGEGTFGQLCRHYG-DQVDRVLG**

**MmuTRZ2 (466) NP------------------------------------------APAEKR----SQYPEIVFLGTGSAIPMKIRNVSSTLVNLSPDKS-----VLLDCGEGTFGQLCRHYG-QQIDRVLC**

**PtrTRZ2 (470) GP------------------------------------------APAEKR----SQYPEIIFLGTGSAIPMKIRNVSATLVNISPDTS-----LLLDCGEGTFGQLCRHYG-DQVDRVLG**

**OcuTRZ2 (466) SP------------------------------------------APAEQS----SQYPEVVFLGTGSAIPMKIRNVSSTLVNISSDTS-----LLLDCGEGTFGQLCRHYG-DDVDRVLG**

**RnoTRZ2 (469) SP------------------------------------------APAEKR----SQYPEIVFLGTGSAIPMKIRNVSSTLVNLSPDKS-----VLLDCGEGTFGQLCRHYG-QQIDRVLC**

**SscTRZ2 (464) DP------------------------------------------PAEER-----GRYPEVVFLGTGSAIPMKIRNVSSTLVNVSSDTT-----LLLDCGEGTFGQLCRHYG-DDVDRVLG**

**GgaTRZ2 (481) VP------------------------------------------GNGN-------PYPEIVFLGTGSAIPMKIRNVSSTLVNTSSTRS-----LLLDCGEGTFGQLCRHYG-EQVDQVLC**

**AcaTRZ2 (473) RL------------------------------------------TMSENT----DCYPEVIFLGTGSAIPMKIRNVSSTLINISATQS-----LLLDCGEGTFGQLCRHYG-DEIDKVLC**

**XtrTRZ2 (479) DG------------------------------------------AFTGES--K-GQYPEVIFLGTGSAVPMKTRNVSSTLVNVSPSHS-----LLLDCGEGTFGQLHRHYG-ENVDEVLS**

**DreTRZ2 (542) AS------------------------------------------VSTSGE-----KFPEIVFLGTGSSLPMKIRNVSGTLVNISSSQS-----LLLDCGEGTFSQMCRHYG-DDVDEMLS**

**GacTRZ2 (508) DA------------------------------------------APPAEG--A-E-HPEVVFLGTGSALPMKIRNVSGTLVNISPSRS-----LLLDCGEGTFGQLCRHYG-DAVDEALS**

**OlaTRZ2 (510) DA------------------------------------------AQLSDG----K-YPEVVFLGTGSALPMKIRNVSGTLVNISPTQS-----LLLDCGEGTFGQLCRHYG-DHVDEALS**

**SsaTRZ2 (512) DA------------------------------------------AVLSGR--A-EKYPEVVFLGTGSALPMKIRNVSGNLVNISATQS-----VLLDCGEGTFGQLCRHYG-DTVDETLA**

**TruTRZ2 (496) DA------------------------------------------AEDGEK------YPEVVFLGTGSALPMKTRNVSGTLVNISSSRS-----IVLDCGEGTFGQLCRHYG-DAVDDVLS**

**TniTRZ2 (475) DA------------------------------------------AEEGEN------FPEVVFLGTGSALPMKIRNVSGTLVHLSSSAS-----LLLDCGEGTFGQLCRHYG-NAVDQVLS**

**CinTRZ2 (404) NS---------------------------------------------------MSEYPRLVVLGSGGNMPHKYRAFPAYLLRVDPNTS-----ILMDCGDGTYTQLYRHFGATNIKKILK**

**CsaTRZ2 (470) NS---------------------------------------------------ISDFPRLVVLGSGSNMCHKHRAHPAYLLRLNQNTS-----LLIDCGDGTFTQLYNHFGPKYYREVLE**

**BflTRZ2 (462) R-------------------------------------------VADDTTVS--QSYPEVVFFGTGSSIPSKRRNVTGILVHLSETES-----LLLDGGEGTFGQMYRHYG-DKVDRVLA**

**SpuTRZ2 (599) TK------------------------------------------SDHTES--TEQKYPEVVFLGTGSAMPNKARNVSGILLNFSENKS-----MIMDCGEGTFGQLCRYYG-DKVDGVMA**

**SkoTRZ2 (421) HVNSSNTSELTGTDESDTVKTEMQRTNCVESASNNVTEPNDSETTSEVDNIVTSVKYPEVVFFGTGSAMPSNARNVSSILLNMNNDTS-----VLMDCGEGTFGQLFRHYG-NQTLDIMQ**

**AaeTRZ1 (480) RK------------------------------------------PQQELV-ARSEKYPRVVFLGTGSSIPNKTRNVSAILVHTGPGSS-----ILLDCGEGAAGQIIRLYGHEQAKNVFR**

**AdaTRZ1 (481) HD------------------------------------------EQRVRT-NRENQFPRLVFLGTGSSIPNKTRNVSATLILTNPQSS-----ILLDCGEGTVGQIVRFFGKTEAETVLR**

**AecTRZ1 (494) RT------------------------------------------KTLHIN----NEYPKIVMLGTGSSIPSKVRNTSGILLQVDKDHS-----MLLDCGEGTFGQIVKIYGKSGAHNILK**

**AgaTRZ1 (485) HT------------------------------------------ARRSAA-VRAEQFPRLIFLGTGSSIPNKTRNVSAILILTSKQSS-----ILLDCGEGTVGQIWRVFGKEQAEEILR**

**ApiTRZ1 (480) LN---------------------------------------------ASP-TDTNIYPKITFLGTGSCIPSKTRNTSGILMYTGENEC-----VLLDSGEGTYGQLVRHFGLTGAETVLS**

**BmoTRZ1 (520) IR------------------------------------------Y--NN--GAQKEYPKLVFLGTGSCIPSKTRNTSCFVLHVDEHSS-----IVLDCGEGSFGQMVRFYGPKKVNAFLR**

**CflTRZ1 (452) EK------------------------------------------ERLSIG----KEYPKIIMLGTGSSIPSKVRNTSGILLRMDQNHS-----MLLDCGEGTFGQIAKIYGKYKMDKIIK**

**CpiTRZ1 (449) Q-------------------------------------------PSQSQT-PRSEIFPRIVFLGTGSSIPNKTRNVSAILVRTSPDSS-----ILLDCGEGTAGQIERLYGVEAAVQVFR**

**CquTRZ1 (449) Q-------------------------------------------PSQSQT-PRSEIFPRIVFLGTGSSIPNKTRNVSAILVRTSPDSS-----ILLDCGEGTAGQIERLYGVEAAVQVFR**

**DpuTRZ1 (399) LA------------------------------------------D--AVP-ISSRVYPRVTFLGTGSCIPNKTRNTSGILVELEKDRF-----ILMDCGEGTYGQIVRFFGHEKAAHVLS**

**DanTRZ1 (449) P----------------------------------------------ASS-TNS--YPKIIFLGTGSCIPNKTRNVSSILIQTAADAY-----VLLDCGEGTYGQIVRLYGRSRAETVLA**

**DerTRZ1 (447) P----------------------------------------------DNS-ADS--YPKIIFLGTGSCIPNKTRNVSSILIRTAIDAY-----VLLDCGEGTYGQIVRLYGREKGQIILR**

**DgrTRZ1 (470) Q----------------------------------------------RPD-TAVGSYPRIIFLGTGSCIPNKTRNVSSILIQTAAEAF-----MLLDCGEGTHGQIVRLYGRERAEQIMR**

**DmeTRZ1 (447) P----------------------------------------------DNS-ADS--YPKIIFLGTGSCIPNKTRNVSSILIRTAIDAY-----VLLDCGEGTYGQIVRLYGHEKGQLILR**

**DmoTRZ1 (450) T----------------------------------------------LKI-ESK-SYPRIIFLGTGSCIPNKTRNVSSILIQTAAEAF-----MLFDCGEGTHGQIVRLFGRERAREVML**

**DsiTRZ1 (447) P----------------------------------------------NNS-ADV--FPKIIFLGTGSCIPNKTRNVSSILIRTAIDAY-----VLLDCGEGTYGQIVRLYGHEKGQLILR**

**DseTRZ1 (447) P----------------------------------------------DNS-ADS--YPKIIFLGTGSCIPNKTRNVSSILIRTAIDAY-----VLLDCGEGTYGQIVRLYGHEKGHLILR**

**DviTRZ1 (465) T----------------------------------------------LKT-PSN-SYPRIIFLGTGSCIPNKTRNVSSILIQTAAEAF-----MLLDCGEGTHGQIVRLYGRERARQVMQ**

**DwiTRZ1 (453) V----------------------------------------------STP-TNT--YPRLIFLGTGSCIPNKTRNVSSILIQTSVKGF-----ILLDCGEGTYGQIIRFFGQPKAQEVLE**

**DyaTRZ1 (447) P----------------------------------------------DNT-ADS--YPKIIFLGTGSCIPNKTRNVSSILIRTAIDAY-----VLLDCGEGTYGQIARLYGREKGQLILR**

**HmeTRZ1 (553) TR------------------------------------------Y--AKR-DSQKEYPKVVFMGTGSCIPSKTRNTSGIVVHISEDRS-----MLLDCGEGTFGQLVRFYGPKKVNSFLR**

**NviTRZ1 (521) KT------------------------------------------KPLGNEKVNVKEYPRLVMLGTGSSIPNKVRNTSGILFQVNEDTS-----IILDCGEATMGQIIRFFGVSEADRVLK**

**TcaTRZ1 (499) AQ------------------------------------------K--RRH-LTVRDFPRLLFLGTGSSIPNKTRNTSGMLLEIDENTS-----IVIDCGEGTVGQIIRFYGQDKANAELA**

**AsuTRZ1 (501) EC------------------------------------------NCT----ECSNDMPRISFLGTSSAVPSKYRNVSGYLLQLSESSS-----VMVDCGEGSYGQLRVLFGVERCLEILL**

**BmaTRZ1 (451) ----------------------------------------------TSNLLANEDNWPRICFLGTSSAVPTNFRNVSAYFLQFNENFC-----IFVDCGEGSYGQLRTLFGDVACEDLLL**

**CbrTRZ1 (475) LE------------------------------------------DSV----ENNCEFPKLTFFGTSSAVPSKYRNVTGYLVEGSDESA-----ILLDVGEGTYGQMKAIFGEVGCKKLLT**

**CreTRZ1 (474) LE------------------------------------------ASI----EKNCEFPKITFFGTSSAVPSKYRNVTGYLVEASNESA-----LLLDVGEGTYGQMKAVFGEEGCKKLLV**

**CelTRZ1 (481) LE------------------------------------------KEN----KMDCEFPKLTFFGTSSAVPSKYRNVTGYLVEASENSA-----ILIDVGEGTYGQMRAVFGEDGCKQLLV**

**LloTRZ1 (459) ----------------------------------------------SSNLLENEDSWPRICFLGTSSAVPTNFRNVSAYFLQFNENSC-----IFVDCGEGSYGQLRTLFGDVVCEDLLL**

**PpaTRZ1 (518) AA------------------------------------------EALGAYCTSDSSFPRVTFLGTSSATPSKYRNVSGYLFETSSSSA-----FLVDVGEATYGQLRVLLDDATCDELML**

**TspTRZ1 (447) QIG------------------------------------------------DQIDQYPAVTFLGTSSASPVKTRNVSALLIHLDDGSA-----VLCDCGESTYSQAYLRYGADGIGPLLR**

**WbaTRZ1 (452) IH------------------------------------------FLASNLLANEDNWPRICFLGTSSAVPTNFRNVSAYFLQFNENFC-----VFVDCGEGSYGQLRTLFGDIVCEDLLL**

**CteTRZ1 (388) LRDS------------------------------------------------EGSEFPRFTFLGTASASPSKYRNVSGILLQAREDQY-----FILDCGESSLIQMILHYGVEKVKDILI**

**LgiTRZ2 (423) LK------------------------------------------SEIPKD--DR-VYPEIVFLGTGASTSSKYRNFSGILLRLDDSKS-----IMLDCGEATISQLYDHYG-EKTDSILR**

**LgiTRZ3 (455) LK------------------------------------------SEIPKD--DR-VYPEIVFLGTGASTSSKFRNFSGILLRLDDSKS-----IMLDCGEATISQLYDHYG-EKTDSILR**

**HroTRZ1 (539) NPP----------------------------------------SPVSTN-------NPEILFLGTGSAVPNKYRNVSSVWLNLGNGKC-----FVMDAGENSYGQLLRHYGVKKGQGKLA**

**ShaTRZ2 (494) EN---------------------------------------------GQS--SVSSYPKVVFLGTGSSEPNRIRAQSCILVQLSKHTN-----VILDCGEDSYGQLYRFFGPKKASRVLR**

**SmaTRZ2 (450) IES-----------------------------------------NKPPAHRLASEAYPEITFLGTASSSPNKYRNISCILMQLDPDNY-----IMLDCGEGSLSQLYALHGVEKGNDILR**

**NveTRZ3 (437) SLG------------------------------------------QGS----VDDFDFEVVFIGTGASLPSKYRNVSSTLLSISNEHS-----VLLDCGEGTLGQLYRHYG-NKADDVIR**

**NveTRZ2 (480) APDS-------------------------------------------EQSARHKEDGFSVTFLGTGASKMSKLRNTSSMLVQIGVDSS-----ILFDCGDGTFCQLKHQYG-KHTGNVLA**

**HmaTRZ2 (385) SSSS------------------------------------DEFLPTKKNDNQSFKEYPKVVFFGTGSSIPSKLRNVSSTLLFFSEDEA-----VLLDCGEECYGQLFRHYG-NKITHILR**

**AquTRZ2 (404) LLSN-LKN------------------------------KLLMEYHEKLTKKLFNSSRTKITFLGTGASNLSITRNVSSILLHLPSGAF-----MLLDAGEGTLSQLYMCFGSTLADEVLR**

**TadTRZ1 (428) SP-------------------------------------------VQRN---LIGRHGEIVFLGTGSCYPSKYRNVSSILLRLNKNSC-----MLMDCGEGTFGQLYRHYG-SELSDILR**

**MbrTRZ2 (480) TK------------------------------------------FEDSASATPSWNNPVVHFLGTGSAVPSKYRNVTSMLVAQHDDEAALQWTLLMDAGEGSTAQLLRLLGPTAQAEHLA**

*** * * ** * *** * ***

**Motif II**

**BtaTRZ2 (529) SLAAVFVSHLHADHHTGLLNILLQRE--RALASLGR--------PCHPLLVVAPTQLRTWLQQYHNQCQPLLHHVSVIPAKCLQKGAEVSS-------------------PEVERLINLL**

**CfaTRZ2 (530) TLAAVFVSHLHADHHTGLLNILLQRE--RALASLGK--------PCHPLLVVAPTQLKAWLQQYHNHCQQLLHHVSLIPAKCLQKGAEVSS-------------------PAVERLIASL**

**CpoTRZ2 (534) SLAAVFVSHLHADHHTGLLNILLQRE--RALASLGK--------PFRPLLVVAPMQLRPWLQQYHRQCQEVLHHISLIPAKWLQKGVEVSD-------------------PTMECLIGSL**

**CjaTRZ2 (537) TLAAVFVSHLHADHHTGLLNILLQRE--RALASLGK--------PFRPLLVVAPTQLKPWLQQYHNQCQKILHHISMIPAKCLQEGAEISS-------------------PAMERLISSL**

**EcaTRZ2 (529) ALAAVFVSHLHADHHTGLLNILLQRE--RALASLGE--------PFHPLLVVAPTQLRAWLQKYHNQCQQILHHVSMIPAKCLQKGAEVCS-------------------PEVERLISTL**

**LafTRZ2 (532) TLAAVFVSHLHADHHTGLLNILLQRE--RALASLGK--------PLHPLLVVAPTQLMAWLQQYHYQCQEILHHVNLIPAKYLQKGAEVSS-------------------PIVETLINSL**

**LgoTRZ2 (538) TLAAVFVSHLHADHHTGLLNILLQRE--QALASLGK--------PLHPLLVVAPSQLKAWLQQYHNQCQEVLHHISMIPAKCLQEGAEISS-------------------PAVERLISSL**

**HsaTRZ2 (538) TLAAVFVSHLHADHHTGLPSILLQRE--RALASLGK--------PLHPLLVVAPNQLKAWLQQYHNQCQEVLHHISMIPAKCLQEGAEISS-------------------PAVERLISSL**

**MmuTRZ2 (534) SLTAVFVSHLHADHHTGLLNILLQRE--HALASLGK--------PFQPLLVVAPTQLRAWLQQYHNHCQEILHHVSMIPAKCLQKGAEVSN-------------------TTLERLISLL**

**PtrTRZ2 (538) TLAAVFVSHLHADHHTGLLNILLQRE--RALASLGK--------PFHPLLVVAPNQLKAWLQQYHNQCQEVLHHISMIPAKCLQEGAEISS-------------------PAVERLISSL**

**OcuTRZ2 (534) TIAAVFVSHLHADHHTGLLNILLQRE--RALASLGK--------PLQPLLVVAPTQLKAWLQQYHNQCQEVLHHVSLIPAKCLQKGAEVLN-------------------PMVEGQISSL**

**RnoTRZ2 (537) NLTAVFVSHLHADHHTGLLNILLQRE--HALASLGK--------PFQPLLVVAPTQLRAWLQQYHNQCQEILHHISMIPAKCLQKGAEVPS-------------------PPVERLISLL**

**SscTRZ2 (531) ALAAVFVSHLHADHHTGLLNILLQRE--RALASLGR--------PLRPLLVVAPTQLRTWLQQYHNHCQQVLQHVSVIPAKCLQQGAEVSN-------------------PDIERLISLL**

**GgaTRZ2 (546) NIVAVFVSHMHTDHHSGLVNILMERR--RAFASLGQ--------AFSPLFLVAPEQIMPWLHEYHNNCESILRDIKMISCQSLVKGCENIK-------------------SKTKWFITSL**

**AcaTRZ2 (541) NIAAVFVSHIHADHHTGLLNILLQRH--RAFMSLGQ--------SPSPLLLVAPTQLMTWLHQYHDHCQEILGHINMIPARFLIEGCDVFK-------------------PKAKAFIASL**

**XtrTRZ2 (548) KLSAIFVSHIHADHHTGLLNILFERE--RGLVTCGK--------PHTPVSVIGPPLLMTWLNQYHNHCQDILHHMNLIPAKYLTDGTEALS-------------------LKNKNLLASF**

**DreTRZ2 (609) KLSTIFVSHLHADHHTGLIQLLLERE--RALNSLGK--------ALSPVYLIAPIQIMSWLNQYHDHCQQILSQINIIPSRLLCDGTESPK-------------------VKTKSFIQAV**

**GacTRZ2 (576) KISSVFISHQHADHHTGLLMLLYQRE--RALRTLGR--------TFSPVYLVAPAHIMNWLGQYHDYCEEILHHINLIPNKALCDGSEPPG-------------------QRTSASIQAL**

**OlaTRZ2 (577) KISTVFVSHLHADHHTGLIKLLFQRE--RALTGLGK--------AFSPVCLVAPVQIMNWLGQYHEHCEEILGHFNLVPNRCLSDGAEAPR-------------------PKTKSLIQAL**

**SsaTRZ2 (581) KISTVFISHMHADHHTGLLSLLFQRE--RALATLGK--------AFSPIYLIGPVQMMTWLNQYHDHCEEILSHVNMVPSKVLCEGAEVSK-------------------FKTKAFIQAL**

**TruTRZ2 (562) KISAVFVSHLHADHHTGLLMLLYQRE--RALAVLGK--------PWRPVHLVAPGHIMNWLQQYHDQCQEILQHFSLIPNRSLQAGGEPAD-------------------PRTAALIRAL**

**TniTRZ2 (541) QISTVFVSHLHADHHTGLLMLLYQRD--RALRQLGR--------PRSPVHLVAPVQIMSWLNQYHQQCQEVLQHVDFIPNRSLQDGGEPPA-------------------PRTEASIQAL**

**CinTRZ2 (468) TIKVIVLTHRHIDHFIGVVHLLKQIAS-----SVPR--------KRDKVSIFAPGMLRNFLFGFIHQNDHSLLEYLYFSAIEKYLTDDYLQS--------------------------YV**

**CsaTRZ2 (534) SIKVITISHRHTDHCIGLVHFLKQMVKFK----RGQ--------MDGKVSVFLPGMLRRFMLEYAGKYERYLLNDVYFHAIERYNEGQ------------------------------DI**

**BflTRZ2 (531) NIKCVFISHIHADHHLGLMRIFQERR--RALQTLGE--------PQQPVFLIAPLPFMSWINHYRLNCENIGIDNKDFIVLLCKDLSVFSS-------------------------EEQS**

**SpuTRZ2 (669) SIQCIFISHIHADHHAGLINLLKHWKR---VTKSDD--------SGNNLILIGPKRMFIWLNLFDQHCESFIHRTRFVELADLNINQQGER---------------------------SR**

**SkoTRZ2 (535) RIQCIYISHLHADHHMGLISLLIERK--KYMVALGG--------DVTPLNLVLPAQLIHFLNLYERLFHKILDNVRIIPCRNLLPFSKDKG---------------------------EN**

**AaeTRZ1 (552) TIKAIYISHLHADHHLGLFGLLQTKR-----KLLGS--------DNEKILLIAPEQISYWLRLYDCRFEPVHKDYVLIKNA--ELVDEP-----------------------------LR**

**AdaTRZ1 (553) SLKAVYISHLHADHHLGLIGLLQTRR-----KLFGD--------SCEPLLLLAPEQISYWLRLYDCRFESIHKDYVLIKNA--DMVS--------------------------------I**

**AecTRZ1 (563) TIKGVYISHMHADHHIGLIGLLKERR-------K-V--------TEDPLYLLAPGHINVWLHMYHTHFEPILHRMTLIKNNEFCMDSHNP--------------------------ELYK**

**AgaTRZ1 (557) SIKTVYISHLHADHHLGLIGLLQARK-----KLLGD--------NCECLTLVAPEQISYWLRLYDCRFETIHKDYVLVKNA--DLLENP-----------------------------LQ**

**ApiTRZ1 (549) DLKAIYVSHLHADHHIGLIGILSVRQKMKANKLFKL---------NQPVYLLAPVQIMTWLNFYDRRFAELSEEFQIVSN--LDLDFESS--------------------------TKLR**

**BmoTRZ1 (589) TVKAIYISHLHADHHIGLIGLLQARREAFEESS--A--------TPEAAYLLAPGQIVSWLSVYNQQFERIQNDYTLIPN--QSLLYDSS--------------------------TEEM**

**CflTRZ1 (521) TIKAVYISHPHADHHIGLIGFLKERK-------K-I--------TQDPLYLFAPTYIAMWLRLYHMRFEPILHQMTLIPNNEFFMDVHEP--------------------------TEHK**

**CpiTRZ1 (520) SIKAVYISHLHADHHLGLFGLLQTRR-----KLLGP--------DSEKVLLLAPEQIFYWMRLYDCRFEPLHKEYELVKNA--QLIEEP-----------------------------LR**

**CquTRZ1 (520) SIKAVYISHLHADHHLGLFGLLQTRR-----KLLGP--------DSEKVLLLAPEQIFYWMRLYDCRFEPLHKEYELVKNA--QLIEEP-----------------------------LR**

**DpuTRZ1 (469) NLVGVYISHLHADHHIGLIGLLQGRQ--HAIERTRS--------DAGPVWLIAPHQINFWLKTYHHSFERIRQYYKLFSCVNLFEKEKP------------------------------D**

**DanTRZ1 (515) QLQAIYVSHLHADHHIGLIGLLRERR-----KLKQK---------VEPLILLAPRQIEPWLEFYNRQIEPIEDSYTLVGNG--ELLESP-----------------------------LT**

**DerTRZ1 (513) QLQAIYVSHLHADHHIGLIGLLRERR-----QLEPS---------ADPLILMAPRQIEPWLEFYNRQIETVEDAYTLVANG--ELLTSP-----------------------------LS**

**DgrTRZ1 (538) QLHAVYISHLHADHHIGLIGLLHERQ-----RLMP----------AAPLLLLAPRQIEPWLQFYNRQIEPIADAYTLVGNG--EMLDQP-----------------------------LA**

**DmeTRZ1 (513) QLQAIYVSHLHADHHIGLIGLLRERR-----QLKPR---------ADPLILLAPRQIEPWLEFYNRQIETVEDAYTLVGNG--ELLASP-----------------------------LS**

**DmoTRZ1 (517) QLQAVYVSHLHADHHIGLIALLRERQ-----RLEP----------TSPLLLLAPRQIEPWLNFYNRQIEPIADAYTLMGNG--ELLEQP-----------------------------LA**

**DsiTRZ1 (513) QLQAIYVSHLHADHHIGLIGLLRERR-----QLEPR---------ADPLILLAPRQIEPWLEFYNRQIETVEDAYTLVGNG--ELLASP-----------------------------LS**

**DseTRZ1 (513) QLQTIYVSHLHADHHIGLIGLLQERR-----QLEPR---------ADPLILLAPRQIEPWLEFYNRQIETVEDAYTLVGNG--ELLASP-----------------------------LS**

**DviTRZ1 (532) QLQAVYVSHLHADHHIGLIGLLRERQ-----RLAP----------ASPLLLLAPRQIEPWLQFYNRQIESIADAYTLVGNG--ELLEQP-----------------------------LA**

**DwiTRZ1 (519) QLQAIYVSHLHADHHMGLIGLLRERK-----QLFPE---------SPPLILLAPRQIEPWLQFYHSQIEDIADSYTLVANG--DLLQQP-----------------------------LS**

**DyaTRZ1 (513) QLQAIYVSHLHADHHIGLIGLLRERR-----QLEPR---------ADPLILLAPRQIEPWLEFYNREIETVEDAYTLVANG--ELLASP-----------------------------LS**

**HmeTRZ1 (623) TLKAIYVSHLHADHHIGLIGVLQARHDAFAEINPEG--------PTPPLYLLAPGQIVTWLSIYHQLFEKIREEFVLVPN--QNLLKNK---------------------------QSDL**

**NviTRZ1 (594) SIKAVYVSHLHADHHLGLVGILKQRK-------R-L--------TDEPVFLLAPQQISWYLNYYHSRFEPIQDLYRLISNRDLLLNQTIL--------------------------SSAT**

**TcaTRZ1 (569) KIRAVYISHLHADHHLGLIGVLQGRQ-RAMQKLEKG---------AQPLFLLAPKQIMWWLNFYDRCFEKISEEFVLVPNGELFFNNHEL--------------------------DQLL**

**AsuTRZ1 (570) SLKAIFITHAHQDHMNGLYTMILRRH--EAFLAKGV--------EYRPLVLVCNFNVLNPLRTYSRCFCDLEPLINVVNISNR------SP-VFRTRGDGRNHLRRGDDSRLSG--IMDI**

**BmaTRZ1 (520) KLSAVFITHGHQDHYHGIFTIVQCRK--ELFMKRGM--------TYKPLIVAGGNHVLKVFRDVDRSFGNYTRDMHIVNISKILWTLSQQK------GGP------------IE--AVDL**

**CbrTRZ1 (544) NLHCVLITHAHQDHMNGLYTIVDRRK--EAFESLGI--------PYRPLVLVCNRNVLKPLKTYSICFENIQSLLEIVDISRHPLTPPSSP----NGPPG--KRPKLPSPHLPP--CRDI**

**CreTRZ1 (543) NLHCVLVTHAHQDHMNGLYTIVARRK--EAFESLRI--------PYRPLVLVCNRNVLKPLKTYSICFENIENLLEIVDISRHPLTPPGSP----NGPPG--KRPRLPSPHLPP--SRDI**

**CelTRZ1 (550) NLNCVLITHAHQDHMNGLYTIIARRK--EAFESLGA--------PYRPLVLVCNRNVLKPMKTYSICFENIEHLLEIVDISRYPLTPPGSP----GGPPG--KRPRLPSPHLPP--SRDV**

**LloTRZ1 (528) KLNAVFITHGHQDHYHGIFTIVQCRK--KLFVKRGL--------TYKPLVVIGGNHVLKVFRDIDRSFGNYTRDMHIVDISKILWTLSQEK------GGP------------VE--AADL**

**PpaTRZ1 (591) NLHAIFITHAHQDHMNGLYTVIEKRR--DAFKMRGL--------PYRRLVLVCNKNVMKPLRTYTHCFEDLERYVEVVDLATSSGGPSHQPPERKNSPPSSPKKVKFDHNHAPSPTTVEL**

**TspTRZ1 (514) SVKLIFISHMHGDHFFGLPTFLRRRFRAYQDCQLEY----------EPVFLVAPQNLLHILTMFESFSGSVKQLCKTILTHRLHPLTEQSQ---------------------------KH**

**WbaTRZ1 (525) KLSAVFITHGHQDHYHGIFTIVQYRK--ELFAKRGM--------TYKPLIVAGGNHVLKVFRDVDRSFGNYTRDMHIVDISKILWTLSQQK------GGP------------VE--AVDL**

**CteTRZ1 (455) NLDLIYISHLHPDHHLGFFTILLERQ--KAFASKGQ--------PYKPLEVVTCQSFENWYRLVETYFGIPVSQLIRHTFVENFQVNFFDR----------------------------I**

**LgiTRZ2 (492) TLNGVFISHMHADHHLGLSGIIQARLDAFG--KEKK--------DITSLVVAAPLSMKRWIDYIDSRIYPVSAAIKLFPLQRLKPEDKLN------------------------------**

**LgiTRZ3 (524) TLNGVFISHMHADHHLGLCGIIQARLDAFG--KEKK--------DIPRLIVAAPSSMKRWINYIDGRIYPVSAAIKFFPLQRLKLEDKLN------------------------------**

**HroTRZ1 (607) DLAGAFVSHLHADHHLGLIGFALERHNYMKSKQNNKNDVIV-NGNDDRLIVIAPRALYRWYSSIHCDDIPILDSIEFIASESLHVGRRER-------------------------AADDV**

**ShaTRZ2 (562) KVKAIFVSHMHADHHLGLFSLLKERK--RAFDEKKL--------PHSSAIVMAPIQMRRWLRFYDQELEPVNHLFRFMHHQQGLAATDEMEVR------------------------TAT**

**SmaTRZ2 (524) KLRLILVTHMHADHHGGVFTVALVRSNLLKSDGIDQS--------NCLLPVLTPSEFCHWLTNFNKLFHYDQIVDPFIIPIIYDKHDKNSN----------------------PSWVLNM**

**NveTRZ3 (505) RISCVFISHMHADHHLRILKRCFSVK-----QVS-D--------TAAHTLVIGPLRMRSWLTEYSSLCEHLDFRFDRDRFDRDRFDRDRFDRDR--------------FDRDRFCRDRFC**

**NveTRZ2 (551) SLKSIFISHKHIDHHMGLVNLLFHRQ--KAALKRKN---------YEPLVVVGPERLLKWLEYYNSRRNELHYRYSELTIIEAQEQHWVKS-----------------------GQCKNQ**

**HmaTRZ2 (463) CIKIILISHMHADHHLHICIISLQNSIPNNNKIPDFGTLNSGMNKDDSVILVAPEIMMNWLNCYKRIDLKSLNFISNEKLMRDGEVLFRN------------------------------**

**AquTRZ2 (488) NLKCIFVSHMHNDHHLGVTGILQKIQ-----KLSGS------SINDDSVLVVGPRHLSDWLLNYSSKFSSLKFRFIAVDKKEISLPSLHLK-----------------------------**

**TadTRZ1 (496) QLKFIFISHMHADHHQGIVEVLLEHKR--ATQSTDG--------YYSPLYVIGPWAINKFLNEYNSYFESVQWRFMSATEFFKEVCVFS-------------------------------**

**MbrTRZ2 (558) KLAVVHISHVHADHLLGMPGLMRARKRARAARGEPN----------SPLCIVGPMAIMKVLRTVGFVPKRDFYFLRNDFCFPAELQQDESS----------------------EVEDSGL**

*** * ****

**Motif III Motif IV HEAT HST**

**BtaTRZ2 (620) LETCGLEE----------FQTCLVRHCKHAFGCALVHMSGWK------------------------------VVYSGDTMPCE-ALVQMGKDATLLIHEATLEDGL-EEEAVEKTHSTTS**

**CfaTRZ2 (621) LGACNLEE----------FQTCLVRHCKHAFGCALVHTSGWK------------------------------VVYSGDTMPCE-ALVQIGKNATLLIHEATLEDGL-EEEAVEKTHSTTS**

**CpoTRZ2 (625) LETCDLKE----------FQTCLVRHCKHAFGCALVHTSGWK------------------------------VVYSGDTMPCE-ALVQMGRDATLLIHEATLEDGL-EEEAVEKTHSTTS**

**CjaTRZ2 (628) LRTCDLEE----------FQTCLVRHCKHAFGCSLVHTSGWK------------------------------VVYSGDTMPCE-ALVRMGKDATLLIHEATLEDGL-EEEAVEKTHSTTS**

**EcaTRZ2 (620) LGACDLEE----------FQTCLVRHCKHAFGCALVHTSGWK------------------------------VVYSGDTMPCE-ALVQMGKDATLLIHEATLEDGL-EEEAVEKTHSTTS**

**LafTRZ2 (623) LETCDLEK----------FQTCLVRHCKHAFGCALVHTSGWK------------------------------VVYSGDTMPCD-ALVQMGKDATLLIHEATLEDGL-EEEAVEKTHSTTS**

**LgoTRZ2 (629) LRTCDLEE----------FQTCLVRHCKHAFGCALVHTSGWK------------------------------VVYSGDTMPCE-ALVRMGKDATLLIHEATLEDGL-EEEAVEKTHSTTS**

**HsaTRZ2 (629) LRTCDLEE----------FQTCLVRHCKHAFGCALVHTSGWK------------------------------VVYSGDTMPCE-ALVRMGKDATLLIHEATLEDGL-EEEAVEKTHSTTS**

**MmuTRZ2 (625) LETCDLEE----------FQTCLVRHCKHAFGCALVHSSGWK------------------------------VVYSGDTMPCE-ALVQMGKDATLLIHEATLEDGL-EEEAVEKTHSTTS**

**PtrTRZ2 (629) LRTCDLEE----------FQTCLVRHCKHAFGCALVHTSGWK------------------------------VVYSGDTMPCE-ALVRMGKDATLLIHEATLEDGL-EEEAVEKTHSTTS**

**OcuTRZ2 (625) LEMCDLKE----------FQTCLVRHCKHAYGCALVHTSGWK------------------------------VVYSGDTMPCE-ALVQMGQDATLLIHEATLEDGM-EEEAVEKTHSTTS**

**RnoTRZ2 (628) LETCDLQE----------FQTCLVRHCKHAFGCALVHSSGWK------------------------------VVYSGDTMPCE-ALVQMGKDATLLIHEATLEDGL-EEEAVEKTHSTTS**

**SscTRZ2 (622) LETCGLKE----------FQTCLVRHCKHAFGCALVHPSGWK------------------------------LVYSGDTMPCE-ALVQMGKDATLLIHEATLEDGL-EEEAVEKTHSTTS**

**GgaTRZ2 (637) LESYDLAE----------FQTCEVQHCKNAFACSVIHKSGWK------------------------------VVYSGDTMPCM-ALVRMGKNATLLIHEATLEDGM-EKEAIEKTHSTTS**

**AcaTRZ2 (632) LEKYDFAK----------FQTCLVRHCKNAFACSMVHKSGWK------------------------------IVYSGDTMPCE-ALVEMGKNASLLIHEATLEDGL-EDEAIEKTHSTTS**

**XtrTRZ2 (639) LEAYQLEQ----------FQTCLVRHCRNAYACSVVHRSGWK------------------------------LVYSGDTMPCD-ALVQMGKDASLLIHEATLEDGL-EEEAIEKTHSTTS**

**DreTRZ2 (700) LKKNELSK----------FQTCFARHCKNAFACSLTHESGWK------------------------------LVFSGDTMPCD-ALVDIGKNATLLIHEATLEDGM-EDEAFEKRHSTTS**

**GacTRZ2 (667) LKENDLVE----------FQTCAVRHCKNAFACSFTHRAGWK------------------------------LAFSGDTMPCD-AFVHTGKNATLLIHEATLEDGL-EEEAVEKRHSTTS**

**OlaTRZ2 (668) LKKNGLQK----------FQTCAVRHCKNAFGCSFTHQTGWK------------------------------LAFSGDTMPCD-ALVHIGKDASLLIHEATLEDEL-EEEAVEKRHSTTS**

**SsaTRZ2 (672) LKKSDLAK----------FQTCPVRHCKNAFACSITHQSGWQ------------------------------LVFSGDTMPCD-ALAHMGKNATLLIHEATLEDGL-EDEAVEKRHSTTS**

**TruTRZ2 (653) LEENGLQK----------FQTCFVRHCKFAFACSFTHQSGWK------------------------------VAFSGDTRPCD-AFVHLGKDATLLIHEATLEDGL-EQEAEEKRHSTTS**

**TniTRZ2 (632) LERNGLEKVP------LQFQTCLVHHCKHAFACSLTHRQGWK------------------------------LAFSGDTRPCD-ALVRLGKDATLLIHEATLEDGL-EQEAAEKRHSTTS**

**CinTRZ2 (549) RRVLNVES----------LTFVPVVHGVRTFGLSLVVGGRK-------------------------------VVYSSDTAPMVPGLLEEGRGADLLIHDCTYTQPKDFRLAEHNMHSTLE**

**CsaTRZ2 (612) QRIFKGKLKT------DEFKFINVVHGVKTYAMSLVYNGFK-------------------------------LVYATDTAPMVPELIQGGKNADLLLHDSSYVDDT--HLADLNLHSTMQ**

**BflTRZ2 (616) QELNSLKKRLG------FTQVVPVLHVSRSYGLVVTHKDDWK------------------------------LVYSGDSMPCD-ALILAGKDATLLIHEATFDDEL-HQEAKRKRHSTIS**

**SpuTRZ2 (751) HEASLLSRFN-----LKEFNTVYVRHCANAYGVTLTHQDGWK------------------------------MVYSGDTMPCD-NLIKAGKGADLLIHEATLEDGM-EEEAKKKRHSMIS**

**SkoTRZ2 (618) EELLHTQLN------LEKMETVTVNHFAQPHGLAITHRDGWK------------------------------IVYSGDTMPCN-GLVDIGMNADILIHESTFEDGL-EDEALDKRHSTMS**

**AaeTRZ1 (628) DERIND-LGL------TEVVTCRVRHCPHSFGVSLSMNSCQKG----------------------EPGEPVKITYSGDTMPCQ-NLIDLGRDSTILIHEATMEDEL-EAEARVKMHSTLS**

**AdaTRZ1 (626) NERLLE-MGI------TEVATCRVRHCPHSFGVALKTVALG----------------------------------IGDTMPCE-SLIELGRGSTVLIHEATMEDEL-VAEARLKMHSTLS**

**AecTRZ1 (641) YRNMYNTLNV------QAVRTVYVEHCPYSYGVSVTLHNGKK------------------------------IVYSGDTMPCA-RLVELGQNCDLLIHEATMEDDL-IEDAKLKFHSTVS**

**AgaTRZ1 (633) DEKLLA-VGI------KEIATCRVRHCPHSFGVALKVASLGTHPET-------------------NIEGDVKITYSGDTMPCE-SLIELGRDSTVLIHEATMEDEL-AAEARIKMHSTLS**

**ApiTRZ1 (632) IKDLLKQTNM------SDIETTLVRHCPNAFGVSFTHINGWK------------------------------VTYSGDTMPCD-SLVKLGKNSNLLIHEATMEDQL-VSEARRKMHSTMS**

**BmoTRZ1 (671) TSAILARIGV------QAIKTCLVSHCPNAFGVAIDLDGTHK------------------------------ITYSGDTLPCE-ELIKIGENSTLLIHEATMEDEL-ALEARMKMHSTTS**

**CflTRZ1 (599) YKSMYKTLNV------QAVKTTYVKHCAHSFGISVTLNNGKK------------------------------IVYSGDTMPCE-NLVKLGQDCDLLIHEATMEDDL-AKEAKMKLHSTTS**

**CpiTRZ1 (596) DERIHD-LGL------TEIATCRVRHCPHSFGVALRMHSSKK-----------------------DDTEPIKITYSGDTMPCQ-DLVELGRDSTVLIHEATMEDEL-EAEARVKMHSTLS**

**CquTRZ1 (596) DERIHD-LGL------TEIATCRVRHCPHSFGVALRMHSSKK-----------------------DDTEPIKITYSGDTMPCQ-DLVELGRDSTVLIHEATMEDEL-EAEARVKMHSTLS**

**DpuTRZ1 (549) P-ALLTGLNL------KSIVTTYVRHCPSAFGVSLTTADGFK------------------------------LTYSGDTMPCL-GLIDIGRDSDLLIHEATMEDGM-EEEAKMKTHSTIS**

**DanTRZ1 (590) GEKVEA-LGI------TAIATCLVRHCPNSFGISLTLAAQHE-------------------------GEPIKITYSGDTMPCL-DLIELGRNSTVLIHEATMEDDL-EEEARVKTHSTVS**

**DerTRZ1 (588) GEQVEP-LGI------TSISTCLVRHCPNSFGISLTLAAKHN-------------------------SEPVKITYSGDTMPCQ-DLIDLGRDSTVLIHEATMEDDL-EEEARLKTHSTVS**

**DgrTRZ1 (612) DERVQQQLGI------ASIATCLVRHCPHAYGISLTLHAQHE-------------------------GEPVKLTYSGDTMPCA-DLVQLGRNSTVLIHEATMEDDL-EEEARIKTHSTVS**

**DmeTRZ1 (588) GEQVER-LGI------TSISTCLVRHCPNSFGISLTLAAKHN-------------------------SEPVKITYSGDTMPCQ-DLIDLGRDSTVLIHEATMEDDL-EEEARLKTHSTVS**

**DmoTRZ1 (591) GERVEQ-LGI------ASIATCLVRHCPHAFGISLTLQAQHE-------------------------GEPIKLTYSGDTMPCA-DLVELGRNSTVLIHEATMEDDL-EEEARIKTHSTIS**

**DsiTRZ1 (588) GEQVEP-LGI------TSISTCLVRHCPNSFGISLTLAAKHN-------------------------SEPIKITYSGDTMPCQ-DLIDLGRDSTVLIHEATMEDDL-EEEARLKTHSTVS**

**DseTRZ1 (588) GEHVEP-LGI------TSISTCLVRHCPNSFGISLTLAAKHN-------------------------SEPIKITYSGDTMPCQ-DLIDLGRDSTVLIHEATMEDDL-EKEARLKTHSTVS**

**DviTRZ1 (606) GEQVDQ-LGI------ASIATCLVRHCPHAFGVSLTLQAQHE-------------------------GAPIKVTYSGDTMPCA-DLVELGRNSTVLIHEATMEDDL-EEEARIKTHSTIS**

**DwiTRZ1 (594) DERLEP-LGI------TSISTCLVRHCPNAFGISLTLEAKHE-------------------------NEPIKVTYSGDTMPCL-DLIELGRDSTILIHEATMEDDL-EEEARIKTHSTIS**

**DyaTRZ1 (588) GEQVKP-LGI------TSISTCLVRHCPNSFGISLILAAKPN-------------------------SEPVKITYSGDTMPCQ-DLIDLGRDSTVLIHEATMEDDL-EEEARLKTHSTVS**

**HmeTRZ1 (706) SSAILSEIGV------QSITTCQVAHCPNAFGVTVSLDHDHS------------------------------VTYSGDTIPCD-ELVVIGKDSTLLIHEATMEDEL-ADEARTKMHSTTS**

**NviTRZ1 (672) SKALYDSLGI------ANISTINVTHCPFAYGIAITLNDGRK------------------------------TCYSGDTVPCE-NMVKLAKDSFLLIHEATMEDGL-EQEAVLKRHSTIS**

**TcaTRZ1 (653) EKQLLEGLQM------RDISTCLVKHCPNAFGVSFVHQNGYK------------------------------ITYSGDTMPSE-NLVQLGKTSDLLIHEATMEDEL-AAEAVIKMHSTTS**

**AsuTRZ1 (671) VKLMPRSLFDKVEWGLTDILAVQVHHTRMANGFVFINSDGRK------------------------------IVFSGDTKPCD-LLVEHGMNADLLIHEATFEDDH-ERDAAFKKHSTMK**

**BmaTRZ1 (610) TGEIPPEIIDIEKLGFKSVIAVKVNHARTAVGYIFTDLKNQK------------------------------FVFSGDTMPCE-QLVNHGKDALVLVHESTFGDDE-EAHALYKKHSTMK**

**CbrTRZ1 (646) LGDMPSIFDK-KKWNLDELKAVQVHHTRMANGFVMR-VAGKR------------------------------IVFSGDTKPCD-LLVEEGQGADILVHESTFEDGH-ENDAMRKRHSTMG**

**CreTRZ1 (645) LDDMPKSFDK-NVWKLEELKAVQVHHTRMANGFVMR-VAGKR------------------------------IVFSGDTKPCD-LLVAEGQDADVLVHESTFEDGH-EEDALRKRHSTMG**

**CelTRZ1 (652) LQDMSSSFDK-KAWKLDELKAVQVHHTRMANGFVMR-VAGKR------------------------------IVFSGDTKPCD-LLVEEGKDADVLVHESTFEDGH-EADAMRKRHSTMG**

**LloTRZ1 (618) TGQMPPEIVNVKSLGFKSIIAVKVNHARTAVGYIFTDLNNRK------------------------------FVFSGDTMPCE-QLVNHGKDALVLVHESTFADDE-EAHALYKKHSTMK**

**PpaTRZ1 (701) LPKIPSEYSDGQTWGLEGVKAVQVHHTRMANGFVFRTLCGKK------------------------------VVFSGDTKPCD-LLVEHGMDADLLIHEATFEDGH-EQDAFRKKHSTMG**

**TspTRZ1 (597) LQEAMLSLN------VKRLEFVPVAHPMGAHGLVLTTVGNKT------------------------------VVYSGDTRPCP-ALVSAGQGADLLIHEATMEDDL-AQEAVDKKHSTIS**

**WbaTRZ1 (615) TGEIPPEIIDIESLGFKSVIAVKVNHARTAVGYIFTDLKNQK------------------------------FVFSGDTMPCE-QLVNHGKDALVLVHESTFGDDE-EAHALYKKHSTMK**

**CteTRZ1 (537) DHSLLKNLN------LSQIRSVHMHHTQFPFGVRLNHADGWS------------------------------VVYSGDTMPCD-ALVKEGHGCDVLIHEATFMEGM-ESEATFKNHSTMP**

**LgiTRZ2 (572) ---IEKLLQL------DDVS----EHCLGAKGLCLKYNGFK-------------------------------LVYSGDTMPCN-NLVEQGKDCDILIHEATFDEGM-EKEARYKTHSTTT**

**LgiTRZ3 (604) ---IAKLLNL------DDFNPVDVNHCLGAKGLCLKYNGFK-------------------------------LVYSGDTMPCN-NLVRHGKDCDILIHEATFDEGM-EKEAKFKMHSTTT**

**HroTRZ1 (701) VVVSPSYEKLLKLTGLKKIETVRVEHTQHPHGLVLHGGNDNDDDDDEKGSDNDNDNRGLSNDNDDDVKCGWKLVYSGDTMPSK-NLIEAGRNCDVLIHEATFDANK-EEEARDKKHCTST**

**ShaTRZ2 (648) ADDVLRELS------LTHYEPVEVEHCFNSYGVTFTHSNGFK------------------------------LAYSGDTRPCD-NLVTAGENCNLLIHEATHEDAL-MENAAVSKHSTFS**

**SmaTRZ2 (614) KRNETENWLKLLEQLDINIRPVKVPHTRSSWAFIIDNPYPFKNLVNSKN----------IAENCSEIQRKWSIVYSGDTPSCP-ELVRAGKNCDLLIHEATVNDEY-VDLAVKAKHSTTS**

**NveTRZ3 (597) RDRFGRDKFVRVG---LSISVVPVDHCAFAYGLVLSHVTGWK------------------------------VVYSGDTRPCK-ELIDAGAGATLLIHEATLEDEM-TSEAIEKKHSTTT**

**NveTRZ2 (637) LKDLVSSLG------LCKLSVVPVVHCDDSHGIVIAHASGWK------------------------------LVYSGDCSPSG-YLVREGADATLLIHEATFLPDYGEKEAKLTGHSTTD**

**HmaTRZ2 (553) ----------------IHVQSVKVDHPGHAYGFVIS-KDRCK------------------------------VTFSGDTLPSN-ELIKAGEDSTILIHEATMEDDK-AEEAFLKKHSTIG**

**AquTRZ2 (568) ------------------MEFVPVCHPTHSHGIILYYKNKWK------------------------------LVYSGDTRPCR-DLVRAGKDASLLIHEATFDDSL-QANAVARLHSTKS**

**TadTRZ1 (575) -DVVKTELG------IIKLKTVYADHCKGAFCIALTHREGWK------------------------------LAYSGDTQPSS-KFVEIGKDADVLIHEATFEDDL-VDEARSRKHSTTS**

**MbrTRZ2 (646) VKATRDALASTLAKMQLSLQTLPVNHCRHATGLSLRSTRGWS------------------------------LTWSGDTRPCD-VFARFCPDVDLMIHEATFEAEL-QADAVGKRHSTTE**

*** * * ***

**Motif V AxDx**

**BtaTRZ2 (698) QAIGVGMRMSAAFIMLTHFSQRYAKIPLFSPDFNE-----K-----VGIAFDHMKVSL-GDLPTVPRLTAPLKALFAGDLEEMEGRRERRELRQVRAALLAGEDVE-------PPQKRAP**

**CfaTRZ2 (699) QAIGVGVRMNAGFTMLNHFSQRYAKVPLFSPDFNE-----K-----VGIAFDHMKVCF-ADFPTVPRLSAPLKALFAGDIEEMEERREKREVRQARAALLSGEQARGP-DDRTPPQKRAL**

**CpoTRZ2 (703) QAIGVGMRMNSGFIMLNHFSQRYAKIPLFSPDFND-----K-----VGIAFDHMKVCF-GDLPTVPKLIPPLKALFAEDIEEMVERKEKRELRLVRAALSQHLAASPD-DDGERLPKRAL**

**CjaTRZ2 (706) QAISVGMRMNAAFIMLNHFSQRYAKVPLFSPDFNE-----K-----VGIAFDHMKVCF-GDFPTVPKLIPPLKALFAGDIEEMEERREKRELRLVRAALLSRKLAGGL-EDGEPQQKQAH**

**EcaTRZ2 (698) QAISVGMRMNAEFIMLNHFSQRYAKIPLFSPDFND-----K-----VGIAFDHMKVCF-RDFPTVPKLTAPLKALFAGDIEEMEERREKRELRLVRAALLSRELAGGP-ADREPQQKRAH**

**LafTRZ2 (701) QAIGVGMQMNARFIMLNHFSQRYAKIPLFSPDFND-----R-----VGIAFDHMKVCF-GDFSTVPKLIPPLKALFADDLEEMEERREKRELRQLRAALLSKDEPLSP-QS----KKRVP**

**LgoTRZ2 (707) QAISVGMRMNAEFIMLNHFSQRYAKVPLFSPNFNE-----K-----VGVAFDHMKVCF-GDFPTMPKLIPPLKALFAGDIEEMEERREKRELRQVRAALLSGELAGGL-EDGEPQQKRAH**

**HsaTRZ2 (707) QAISVGMRMNAEFIMLNHFSQRYAKVPLFSPNFSE-----K-----VGVAFDHMKVCF-GDFPTMPKLIPPLKALFAGDIEEMEERREKRELRQVRAALLSRELAGGL-EDGEPQQKRAH**

**MmuTRZ2 (703) QAINVGMRMNAEFIMLNHFSQRYAKIPLFSPDFNE-----K-----VGIAFDHMKVCF-GDFPTVPKLIPPLKALFAGDIEEMVERREKRELRLVRAALLTQQADSPE-DR-EPQQKRAH**

**PtrTRZ2 (707) QAISVGMRMNAEFIMLNHFSQRYAKVPLFSPNFNE-----K-----VGVAFDHMKVCF-GDFATMPKLIPPLKALFAGDIEEMEERREKRELRQVRAALLSRELAGGL-EDGEPQQKRAH**

**OcuTRZ2 (703) QAIGVGMRMKAQFIMLNHFSQRYAKIPLFSPDFNE-----K-----VGIAFDHMKVCF-GDFATVPKLIPLLKALFAGEIEEMQERREKRELRLVREALLSQERAGSP-RDGEPQQKRAH**

**RnoTRZ2 (706) QAIGVGMRMNAEFIMLNHFSQRYAKIPLFSPDFNE-----K-----VGIAFDHMKVCF-GDFPTVPKLIPPLKALFADDIEEMVERREKRELRLVRAALLTQQADSSE-DR-EPHQKRAH**

**SscTRZ2 (700) QAIGVGMRMHAEFILLNHFSQRYAKIPLFSPDFTE-----K-----VGIAFDHMKVSL-GDLPTVPKLMAPLKALFAGDLEEMEERREKRELRQVRAALLAEVGDGE------PPQKRAH**

**GgaTRZ2 (715) QAIEIGMKMNAEFIMLNHFSQRYAKIPLFNEDFSE-----K-----VGIAFDHMRVRL-GDFSTIPKLIPPLKALFADDIVEMEERKEKREQRLLKEAAVVLDQLTGGENKKTPSQKRKQ**

**AcaTRZ2 (710) QAIGVGMKMNAEFIMLNHFSQRYAKIPLFSEDFSE-----K-----VGIAFDHMQVRF-CDSAVIPKLILPLKALFADEIEEMEERREKRELRLLQEAFKMSEAQTVDGKKTPAKMKLEL**

**XtrTRZ2 (717) QAIGVGMKMNANFIMLNHFSQRYAKLPLFSSDFSE-----K-----VGISFDHMRINL-GNVRTVPKLVNPLKVLFAEDLEELEERREKRELKQMREMEDQPNGSQPVANHKRDLEGASH**

**DreTRZ2 (778) QAIGIGMKMNAGFIVLNHFSQRYAKIPLFSDDFDN-----K-----VGISFDHMRIQF-GDFPMLPRLIRPLKALFAEEIEEMEERREKRELK--------KSFEPPANGESGKSKAAKR**

**GacTRZ2 (745) QAIGIGMRMNADFIMLNHFSQRYAKIPLFSEDFSD-----R-----VGISFDHMRIRF-SDFKILPRLVPALKTLFAEEIGEMEVRRERRELRNPRGNGSSEATPPVMGRDARRGREEAE**

**OlaTRZ2 (746) QAIGIGMKMNAGFIMLNHFSQRYAKIPLFSDDFTD-----R-----VGISFDHMRIRF-GDFKTLPKLIPALKTLFAEEIGEMEERRERRELRCPRGGSGEPDAEQRTSRSSEEGR--GA**

**SsaTRZ2 (750) QAIGIGMKMNAEFIMLNHFSQRYAKIPLFSEDFND-----R-----VGISFDHMRIRF-GDFRILPRLIPPLKALFAEEIGEMEERRGRRELKQMKGVIAESNEEQRIPNEGETVKGAKR**

**TruTRZ2 (731) EAIGVGVKMNAAFIVLNHFSQRYAKIPLMSADATE-----R-----VGISFDHMKIRF-GHFKTLPRLVPALKTLFAADIGEMEEKREKRELR---GNG--EQPPGAAARPAKRQQEAPS**

**TniTRZ2 (714) QAIGVGVRMNAAFIVLNHFSQRYAKIPLFSQDFTQ-----R-----VGVSFDHMRIRF-GHFRTLPRLVPALKALFAADIGEMEERREKRELR---L-----------------------**

**CinTRZ2 (628) GAIQTAETMEAKSLLLNHFGVKEPMVPPIEESTVPYNG-------TIVTAYDHMEIPLNETIHDFKQLYPSFYHVFQDTMARNSATCALDNDEQNFNDVDLSELFNQ-------------**

**CsaTRZ2 (693) GAVDTADAMNAKALMLIHFAGKTPVVPFTHRPPFTYKG-------PIIAAYDHMELPL-NSEEGIVKFAALHQDMLHIHQGSLERTHWRKTMMLGHNSDSELKSDAACV-----------**

**BflTRZ2 (698) QAVDVGLQMNASFNLLTHFSQRYPKIPLMDHGGEK-----------VGIAFDHMKVRL-GDLKLLPHLSSPLQALFQEDLEEMKEKQKRHKRNRLGGLIE--------------------**

**SpuTRZ2 (834) QAIEVGQSMEAKFLLLTHFSQRYPKVPLIETSSTS-----K-----IGIAFDNMRVSF-SELDLLPQFLPTLKYLYAAEIQELEVLREKKAERKVINKQMTKKKMPSTS-----------**

**SkoTRZ2 (700) QAIDISNQMKAKFVLLTHFSQRYPKIPIIDAKDDR-----TQK---TGIAFDHMKVTI-EDLPILPQLVPVLQCLFLEAIMEMEERTFKRTQRERALAE---------------------**

**AaeTRZ1 (717) QAIEQGKKMNAKYTLLTHFSQRYAKIPRIESELET-----N-----LGIAFDNMEVTL-DDLPQLSLFYPALKAMFISHFEEMEQKAIKRGNKRMRLEGGS-GNNSKESSPTR-------**

**AdaTRZ1 (703) QAIEQGRKMGARYTLLTHFSQRYAKIPRLRPDQQR-----EGLGADLGIAFDNMEVTL-DDLPTLCNMYPALKAMFISHFEEMEQKAVKRGNKKMRLEVAR--NGSKESSPTRVAAEKK-**

**AecTRZ1 (723) QAIQAGEKMKSKFTLLTHFSQRYSVIPHLPDNK-N-----GPKLDNVGIAYDNMHISL-SQLPLLPLMYPTLKIMFNKYYLEIEDRAARRQRIAA-------------------------**

**AgaTRZ1 (725) QAIEQGRKMNARYTLLTHFSQRYAKIPRLRPDQQQ-----TGLGTDLGIAFDNMEVTL-DDLPTLCKFYPALKAMFISHFEEMEQKAIKRGNKKLRLETVKTATGSKECSPTR-------**

**ApiTRZ1 (714) QAINIGKKMNAKFTILTHFSQRYAKIPYMPNND---------LPSNVGIAFDNMEIAP-NVLHKLPLMYPALKMIFAEHFEEMEAKCFKLKLKEEKAKAQM-------------------**

**BmoTRZ1 (753) QAIAAGRQMRAAYTLLTHFSQRYARLPRLSARMLT-------DNRSVGVAFDNMQVTM-SDLALLPHMYAPLQLMFAEHCVEMEMKAASRAKQKERRISTHSFQPE----EKAESVQSDR**

**CflTRZ1 (681) QAIRAGEQMRSKFTLLTHFSQRYSVIPPLPNDD-N-----GSKLNNVGIAYDNMHISL-SQLPLLPLMYPTLKLMFIKHYMEVEERAARR-RIAMNL-----------------------**

**CpiTRZ1 (684) QAISQGRKMNARFTLLTHFSQRYAKIPRIESELDS-----N-----LGIAFDNMDVTI-EDLPQLCLFYPALKAMFISHFEEMEQKAIKRGNKKMRLETGS-GGSSKEPSPAR-------**

**CquTRZ1 (684) QAISQGRKMNARFTLLTHFSQRYAKIPRIESELDS-----N-----LGIAFDNMDVTI-EDLPQLCLFYPALKAMFISHFEEMEQKAIKRGNKKMRLETGS-GGSSKEPSPAR-------**

**DpuTRZ1 (630) QAIEVGRSMGAKFTLLTHFSQRYSKIPIFNEK-AF-------SSNTIGVAFDNMTISP-NRFHHLPYFIPALKLMFADHCEEMENKTNKRKLRQEREENEIQRQLKQSIKS---------**

**DanTRZ1 (676) QAIQQGRNMNARHTILTHFSQRYAKCPRLPDAEDM-----KQ----VAIAFDNMQVTV-DDLQHYHKLYPALLAMYAEYTEELEQRAVKRELKQERKRKMV------QT-----------**

**DerTRZ1 (674) QAIQQGRNMNARHTILTHFSQRYAKCPRLPSDEDM-----QR----VAIAFDNMEVTI-EDLQHYHKLYPALFAMYAEYTEELEQRAVKRELKQERKRKLA------ET-----------**

**DgrTRZ1 (699) QAIQQGRDMQAKHTILTHFSQRYAKCPRLPSSEDM-----QH----VAIAFDNMQVTV-DDLKHYHRLYPALLAMYAEYTEELEQRAVKRELKQERKRKLA------QT-----------**

**DmeTRZ1 (674) QAIQQGRNMNARHTILSHFSQRYAKCPRLPSDEDM-----QR----VAIAFDNMEVTV-EDLQHYHKLYPALFAMYAEYTEELEQRAVKRELKQERKRKLA------ET-----------**

**DmoTRZ1 (677) QAIQQGRDMQAKHTILTHFSQRYAKCPRLPSTEDM-----QH----VAIAFDNMQVAV-EDLQHYHKLYPALLAMYAEYTEELEQRAVKRELKLDRQMTER------QR-----------**

**DsiTRZ1 (674) QAIQQGRNMNARHTILTHFSQRYAKCPRLPSDEDM-----QR----VAIAFDNMEVTI-EDLQHYHKLYPALFAMYAEYTEELEQRAVKRELKQERKRKLA------ET-----------**

**DseTRZ1 (674) QAIQQGRNMNARHTILTHFSQRYAKCPRLPSDEDM-----QR----VAIAFDNMEVTI-EDLQHYHKLYPALFAMYAEYTEELEQRAVKRELKQERKRKLA------ET-----------**

**DviTRZ1 (692) QAIQQGRDMEAKHIILTHFSQRYAKCPRLPSSEDM-----QH----VAIAFDNMQVTV-DDLQHYHKLYPSLLAMYAEYTEELEQRAVKRELKQERKRKLA------QT-----------**

**DwiTRZ1 (680) QAIQQGRDMQAKHTILTHFSQRYAKCPRLPSVDDM-----KH----VAIAFDNMQVTL-EDLQDYNKLYPALMAMYAEYTEELEQRAVKRELKQERKRKLA------ET-----------**

**DyaTRZ1 (674) QAIQQGRNMNARHTILTHFSQRYAKCPRLPSDEDM-----QR----VAIAFDNMEVTI-EDLQHYHKLYPALFAMYAEYTEELEQRAVKRELKQERKRKLA------ET-----------**

**HmeTRZ1 (788) QAINIGKKMNAKYTVLTHFSQRYARLPRLSAHILN-------DNNSVGIAFDNMQITM-SDLELLPHMYAPLQLMFADHCVELELKAEHRARQKEKKPSPAKIPSSGTIRNYSTQITTDK**

**NviTRZ1 (754) QAVSIGVKANVNFTLLTHFSQRYSKIPRLPSVEQS-----GIDFARVGIAFDFMKISF-AQLNLLPLFYPSLNLMFSEFKALLDERASKREWTKEKIVKAAS------------------**

**TcaTRZ1 (735) QAIEIGRQMEAKYTLLTHFSQRYAKLPRFNEN----------FSDNVGIAFDNMRVKL-DELPLIPLLYPALKLMFVEYYEEMEQKAAKRQMKIEREKAQQFVT----------------**

**AsuTRZ1 (759) QAVDVGVRMHAKHIILSHFSARYPKVPALPDYLDS-----AGN---VSVAVDNLSVRF-GDLALLPKTLNVFRELYQEELFEIKLRMEQRTLRKQAEEEQKQSGNSGTMGVDGTRSQTRK**

**BmaTRZ1 (698) QAFDVAARMGAKNLVLTHFSAKYPKVPPLPDYIEK-----AGN---VTIAMDNMIVTP-SDLKLSAKLIPVLRAVFSKEIIEIMQRSLKRHVQEDTLARKFVDTLGCTAQKKKPSENLSF**

**CbrTRZ1 (732) QAVDVGKRMNAKHIILTHFSARYPKVPVLPQYLDT-----DN----IGIAMDMLRVRF-DHLSLVPKLLPIYREVFVSELFELAIKKEQRNLKDKESLEKNEQRKAVKQSS---------**

**CreTRZ1 (731) QAVDVGKRMNAKNIILTHFSARYPKVPVLPQYLDT-----EN----IGVAMDMLRVRF-DHLPLVSKLLPIYREVFVAELFELTIKKEQRILKDKESSEKKQQKRSVKQSS---------**

**CelTRZ1 (738) QAVDVGKRMNAKHIILTHFSARYPKVPVLPEYLDK-----EN----IGVAMDMLRVRF-DHLPLVSKLLPIFREVFVAELFELTIKKEQRVLKDKELSEKRGQLKA--------------**

**LloTRZ1 (706) QAFDVATRMGAKNLVLTHFSAKYPKVPPLPDYIEK-----AGN---VTIAMDNMIVTP-SDLKLSAKLIPVLRTVFAKEIIEIMQRSLKRHIQEDILARRFVDTLGGTVQKKKASGNLAF**

**PpaTRZ1 (789) QAVEIAEKMRAKHCILTHFSARYPKVPPLPSYLDD-----KK----IGLAMDGLRVGF-DRLSLVPLMTPIYRHVYRDELFECELKKCQKDLKKN---RQEI------------------**

**TspTRZ1 (679) EAIAVGRAMGAGFTLLTHFSGRYNKLPLVDERHCS-----ES----IGLAFDFMRVSL-AELAVLTEILPVLKLIFRDTYQNMLMRTEKKNFRRELKLKQLKQPQTQ-------------**

**WbaTRZ1 (703) QAFDVAARMGAKNLVLTHFSAKYPKVPPLPDYIEK-----AGN---VTIAMDNMIVTP-SDLKLSAKLIPVLRTVFSKEIIEIMQRSLKRHVQEDILARRFVDTLGCTAQKKKASENLSF**

**CteTRZ1 (619) QALKIAREMEAKFTLLTHFSQRFPKIPLMPPEKTR-----N-----AGSAVDHLRVSM-KDLHLVPLFKPLLLQLFKEDHHEVLRKMSSWMEREANREPRKKHKVNQRKRNYKNFSPLGE**

**LgiTRZ2 (646) QAIEIGQKMNAKQIILTHFSQRYSKVPIFTDKFTS-----N-----VGIAFDHMTVRP-CDYPYLSLLLKPLAVMFEDAINEINDVKKKTNQK------KDGIQNCADNT----------**

**LgiTRZ3 (682) QAIEIGQKMKAKQIILTHFSQRFKKVPNFTNIHTS-----N-----VSIAFDHMTVRP-CDYPYLSLLLKPLAVMFEDAIKINNIIKMKRTQKDALKLESEAAQVKSHSS----------**

**HroTRZ1 (819) QAIEVGRQMKASFTILTHFSQRYDKLPIFTDVFGD-----D-----VGVAFDNMRVTL-KDLATLPKLVPAYKRILASEYYEMTQQLENKQFRKEFLATAATTVATKAAATLTTELNR--**

**ShaTRZ2 (730) EAMSVGAKMKAENIILTHFSQRYAHMVPFFNMDMP-----HN----VGIAFDNMQVCP-RTMKSLPKVIPALTAIFSDELQKLEIRNIKRTREQEAKQSVEER-----------------**

**SmaTRZ2 (722) QAIQAGRDMNASFILLNHFSQRYGRVPPIDEFKSD-----------VAASFDFMTVKF-SDLQRLPYYIPYYQYAFAKHWNMARTKADSYTYRKLREADELLESEDGHCAKTNDGVDDYN**

**NveTRZ3 (682) EAISSGLEMSARFIMLNHFSQRYPKIPVFNEKFTK-----H-----TGIAFDHMTIRP-RDFDKIPSLLPALKVLFAEEVEELTQNTEIRRNKLNANES---------------------**

**NveTRZ2 (720) GAIEVSKRMRARYTILTHFSQRYNLKRIVKCRFPPG----------VSVAFDHMTVTS-QDLPSLDRVLPNLRRALYGNKPQSSIKKQRWTNDRSPSHFEVQTRGAILSKSIGKSIIGR-**

**HmaTRZ2 (624) QAFVVAQKMKAKNLLLTHFSQRYPKIPNLNDIKSSIP---------FVLAFDHMEVSM-DMIDEFESILPQLNFLFSIDTQEIQEVHENAQVKTQINAQNNIREDTIKAS----------**

**AquTRZ2 (638) EAIQVARHMNAAFLMLTHFSQRYHNHMRHHDHSCFSLHLFEDLPSNVAVAYDFMTIDM-DELDKLSDVSSTISKIK--------------------------------------------**

**TadTRZ1 (656) EAIEIGVKMNAKFTILTHFSQRYPKIPAFSENYSD-----R-----TGIAYDHMTVNS-ESILFLPRLLKPLNIIFKEEEEIED------------------------------------**

**MbrTRZ2 (734) EAITIANDTRARALLLTHFSQRYPRIPKPESAKPN---------MPTAVAFDLMSIPL-RAVEGLAPYLDVLQYVFEQFYSEEDADELE-------------------------------**

*****

**BtaTRZ2 (800) TEHPLSP--QSKKARAQ-------------**

**CfaTRZ2 (807) VEQPRSP--QSKKVRAQ-------------**

**CpoTRZ2 (811) AEQPQSP--QSKKARAS-------------**

**CjaTRZ2 (814) TEEP-----QSKKVRAQ-------------**

**EcaTRZ2 (806) AEEPQNP--QSKKVRAN-------------**

**LafTRZ2 (805) RDEPPSP--QSKKARAQ-------------**

**LgoTRZ2 (815) TEEP-----QAKKVRAQ-------------**

**HsaTRZ2 (815) TEEP-----QAKKVRAQ-------------**

**MmuTRZ2 (810) TDEPHSP--QSKKVRAQ-------------**

**PtrTRZ2 (815) TEEP-----QAKKVRAQ-------------**

**OcuTRZ2 (811) TGEPHSP--QSKKVRA--------------**

**RnoTRZ2 (813) SEEPHSP--QSKKVRAQ-------------**

**SscTRZ2 (803) AERPPSP--QSKKVRAQ-------------**

**GgaTRZ2 (824) DKNHQEV--SNKKLKKLNTVN---------**

**AcaTRZ2 (819) AEESHAA--PNKKLKTTN------------**

**XtrTRZ2 (826) TMGNK-------RLKAN-------------**

**DreTRZ2 (879) EQEDHNQENANKRLKAN-------------**

**GacTRZ2 (854) PQGQQ-----AKRPKTS-------------**

**OlaTRZ2 (853) KRGQQEALMDTKRLKSS-------------**

**SsaTRZ2 (859) EPEEPTQDAGSKRLKTN-------------**

**TruTRZ2 (835) GGGVQ-----GKRAKSS-------------**

**TniTRZ2 (797) ------------------------------**

**CinTRZ2 (728) ------------------------------**

**CsaTRZ2 (794) ------------------------------**

**BflTRZ2 (786) ------------------------------**

**SpuTRZ2 (932) ------------------------------**

**SkoTRZ2 (790) ------------------------------**

**AaeTRZ1 (818) ------------------------------**

**AdaTRZ1 (814) ------------------------------**

**AecTRZ1 (811) ------------------------------**

**AgaTRZ1 (832) ------------------------------**

**ApiTRZ1 (805) ------------------------------**

**BmoTRZ1 (861) GQHRKSPRSRAASSSPTARARYRESNASR-**

**CflTRZ1 (770) ------------------------------**

**CpiTRZ1 (785) ------------------------------**

**CquTRZ1 (785) ------------------------------**

**DpuTRZ1 (732) ------------------------------**

**DanTRZ1 (769) ------------------------------**

**DerTRZ1 (767) ------------------------------**

**DgrTRZ1 (792) ------------------------------**

**DmeTRZ1 (767) ------------------------------**

**DmoTRZ1 (770) ------------------------------**

**DsiTRZ1 (767) ------------------------------**

**DseTRZ1 (767) ------------------------------**

**DviTRZ1 (785) ------------------------------**

**DwiTRZ1 (773) ------------------------------**

**DyaTRZ1 (767) ------------------------------**

**HmeTRZ1 (900) VENIDIMTDSKQIVNNLIDNVDNKSDIGS-**

**NviTRZ1 (850) ------------------------------**

**TcaTRZ1 (828) ------------------------------**

**AsuTRZ1 (870) RKAAGDADT---------------------**

**BmaTRZ1 (809) SDAVPSITS---------------------**

**CbrTRZ1 (833) ------------------------------**

**CreTRZ1 (832) ------------------------------**

**CelTRZ1 (834) ------------------------------**

**LloTRZ1 (817) SDSIPSVTS---------------------**

**PpaTRZ1 (878) ------------------------------**

**TspTRZ1 (776) ------------------------------**

**WbaTRZ1 (814) SDAVPSITS---------------------**

**CteTRZ1 (728) GHSSSSTEDLSPKRVSPSAKTKQAKKMTTN**

**LgiTRZ2 (739) ------------------------------**

**LgiTRZ3 (781) ------------------------------**

**HroTRZ1 (926) ------------------------------**

**ShaTRZ2 (823) ------------------------------**

**SmaTRZ2 (830) RLLNEQSVKG--------------------**

**NveTRZ3 (770) ------------------------------**

**NveTRZ2 (828) ------------------------------**

**HmaTRZ2 (724) ------------------------------**

**AquTRZ2 (713) ------------------------------**

**TadTRZ1 (729) ------------------------------**

**MbrTRZ2 (813) ------------------------------**
