# Supplementary material for: Identification and Sequence Analysis of Metazoan tRNA 3′-End Processing Enzymes tRNase Zs
Source: PLoS One. 2012 Sep 4;7(9):e44264. doi: 10.1371/journal.pone.0044264 (PMC3433465; doi:10.1371/journal.pone.0044264)
Supplement: Figure S2 — Alignment of candidate tRNase ZSs identified in metazoans. The accession numbers for the candidates are listed in Table S1. The annotation of the alignment is described in the legend to Figure 3. (DOC) [file pone.0044264.s002.doc]

**Figure S2: Alignment of candidate tRNase Z^S^s identified in metazoans**

**PxPxRG loop Motif I**

**AmeTRZ1 ( 1 ) ----------------------------------------------------MSMDVTFLGTGAAYPSPTRGASALVLRCE----GECWLFDCGEGTQTQLMKSQLKAG-RITKIFITHL**

**BtaTRZ1 ( 1 ) ----------------------------------------------------MSMDVTFLGTGAAYPSPTRGASALVLRCE----GECWLFDCGEGTQTQLMKSQLKAG-RITKIFITHL**

**CpoTRZ1 ( 1 ) ----------------------------------------------------MSMDVTFLGTGAAYPSPTRGASALVLRCE----GECWLFDCGEGTQTQLMKSQLKAG-RITKIFITHL**

**ChoTRZ1 ( 1 ) ----------------------------------------------------MSMDVTFLGTGAAYPSPTRGASALVLRCE----GDCWLFDCGEGTQTQLMKSQLKAG-RITKIFITHL**

**CjaTRZ1 ( 1 ) ----------------------------------------------------MSMDVTFLGTGAAYPSPTRGASAVVLRCE----GECWLFDCGEGTQTQLMKSQLKAG-RITKIFITHL**

**CfaTRZ1 ( 1 ) ----------------------------------------------------MSMDVTFLGTGAAYPSPTRGASALVLRCE----GECWLFDCGEGTQTQLMKSQLKAG-RITKIFITHL**

**EteTRZ1 ( 1 ) ----------------------------------------------------MSMDVTFLGTGAAYPSPTRGASALVLRCE----GECWLFDCGEGTQTQLMKSQLKAG-RITKIFITHL**

**EeuTRZ1 ( 1 ) ----------------------------------------------------MSMDVTFLGTGAAYPSPTRGASALVFRCE----GECWLFDCGEGTQTQLMKSQLKAG-RITKIFITHL**

**EcaTRZ1 ( 1 ) ----------------------------------------------------MSMDVTFLGTGAAYPSPTRGASALVLRCE----GECWLFDCGEGTQTQLMKSQLKAG-RITKIFITHL**

**HsaTRZ1 ( 1 ) ----------------------------------------------------MSMDVTFLGTGAAYPSPTRGASAVVLRCE----GECWLFDCGEGTQTQLMKSQLKAG-RITKIFITHL**

**LafTRZ1 ( 1 ) ----------------------------------------------------MSMDVTFLGTGAAYPSPTRGASAVVLRCE----GECWLFDCGEGTQTQLMKSQLKAEGRITKIFITHL**

**LgoTRZ1 ( 1 ) ----------------------------------------------------MSMDVTFLGTGAAYPSPTRGASAVVLRCE----GECWLFDCGEGTQTQLMKSQLKAEGRITKIFITHL**

**OcuTRZ1 ( 1 ) ----------------------------------------------------MSMDVTFLGTGAAYPSPTRGASAVVLRCE----GECWLFDCGEGTQTQLMKSQLKAG-RITKIFITHL**

**OprTRZ1 ( 1 ) ----------------------------------------------------MSMDVTFLGTGAAYPSPTRGASAVVLRCE----GECWLFDCGEGTQTQLMKSQLKAG-RITKIFITHL**

**OgaTRZ1 ( 1 ) ----------------------------------------------------MSMDVTFLGTGAAYPSPTRGASAVVLRCE----GECWLFDCGEGTQTQLMKSQLKAG-RITKIFITHL**

**RnoTRZ1 ( 1 ) ----------------------------------------------------MSMDVTFLGTGAAYPSPTRGASAVVLRCE----GECWLFDCGEGTQTQLMKSQLKAG-RITKIFITHL**

**MeuTRZ1 ( 1 ) ----------------------------------------------------MSMDVTFLGTGSAYPSPTRGASAVALRCE----GSCWLFDCGEGTQTQFMKSQLKAG-RITKIFITHL**

**MmuTRZ1 ( 1 ) ----------------------------------------------------MSMDVTFLGTGAAYPSPTRGASAVVLRCE----GECWLFDCGEGTQTQLMKSQLKAG-RITKIFITHL**

**NleTRZ1 ( 1 ) ----------------------------------------------------MSMDVTFLGTGAAYPSPTRGASAVVLRCE----GECWLFDCGEGTQTQLMKSQLKAG-RITKIFITHL**

**PtrTRZ1 ( 1 ) ----------------------------------------------------MSMDVTFLGTGAAYPSPTRGASAVVLRCE----GECWLFDCGEGTQTQLMKSQLKAG-RITKIFITHL**

**SarTRZ1 ( 1 ) ----------------------------------------------------MAMDVTFLGTGAAYPSPTRGASALVLRCE----GECWLFDCGEGTQTQLMKSQLKAG-RITKIFITHL**

**StrTRZ1 ( 1 ) ----------------------------------------------------MSMDVTFLGTGAAYPSPTRGASAVVFRCE----GECWLFDCGEGTQTQLMKSQLKAG-RITKIFITHL**

**TsyTRZ1 ( 1 ) ----------------------------------------------------MSMDVTFLGTGAAYPSPTRGASAMVLRCE----GECWLFDCGEGTQTQLMKSQLKAG-RITKIFITHL**

**AcaTRZ1 ( 1 ) ----------------------------------------------------MSMDVTFLGTGAAYPSPTRGASATVVRFE----GECWLFDCGEGTQTQFMKSQLKAG-RITKIFITHL**

**XtrTRZ1 ( 1 ) ----------------------------------------------------MSMDVTFLGTGSAYPSPCRGASAVVFRTE----GECWLFDCGEGTQTQFMRSPLKAG-RITKIFITHL**

**DreTRZ1 ( 1 ) ----------------------------------------------------MSMDVTFLGTGSAYPSPHRGASALVLRTE----GENWLFDCGEGTQIQLMKSTLRAG-KISKVFISHL**

**GacTRZ1 ( 1 ) ----------------------------------------------------MTMDVTFLGTGSAYPSPHRGASALVLRTE----GECWLFDCGEGTQTQLMKSPLRAG-RITKVFISHL**

**OlaTRZ1 ( 1 ) ----------------------------------------------------MTMDITFLGTGSAYPSPHRGASALVLRLD----GDCWLFDCGEGTQTQLMKSQLRAG-RITKVFISHL**

**SsaTRZ1 ( 1 ) ----------------------------------------------------MTMDLTFLGTGSAYPSPHRGASALVLRTE----GECWLFDCGEGTQTQLMKSQLKAS-RITKVFISHL**

**TruTRZ1 ( 1 ) ----------------------------------------------------MTMDVTFLGTGSAYPSPHRGASALVLRTE----GECWLFDCGEGTQTQLMKSQLRAG-RITKVFISHL**

**TniTRZ1 ( 1 ) ----------------------------------------------------MTMDVTFLGTGSAYPSPHRGASALVLRTE----GECWLFDCGEGTQTQLMRSQLKAG-RISKVFISHL**

**BflTRZ1 ( 1 ) ----------------------------------------------------MSMDLTFLGTASCYPMPNRGVSCIVFKSD----RQCWMFDCGEGSQTQVMKSSIKAA-KINKIFITHL**

**CinTRZ1 ( 1 ) ------------------------------------------------------MDVAFLGTGSGYPTPCRGASALAIRYEG----DVWLIDCGEGTQTQIMKLKTIRPGQISKIFITHL**

**CsaTRZ1 ( 1 ) ------------------------------------------------------MDVAFLGTGSGYPTPCRGASAIALRYEG----EVWLVDCGEGTQTQVMKLKTIRPGQISKIFITHL**

**OdiTRZ1 ( 1 ) ---------------------------------------------------MSDLQVTCLGTASQQPSVKRALNSCVYRNK----GISYLVDCGEGTQAQLTASGVRPS-SIKTVLITHL**

**SkoTRZ1 ( 1 ) ------------------------------------------------------MDLTFLGTASCYPCPNRGVSCTVLRNE----GDCWMFDCGEGSQIQIQKSNIRPT-RITKIFITHL**

**SpuTRZ1 ( 1 ) ------------------------------------------------------MDLIFLGTASAHPSPSRGVSGTVLRSE----REAWLIDCGEGTQIQLMRSNVRPG-RITKIFITHL**

**ShaTRZ1 ( 1 ) ---------------------------------------------------MNTLALTFLGTASAYPTPSRCVSCTALSHD----EGIWLFDCGEGAQVQLMKSPLKPG-KISKIFITHL**

**SmaTRZ1 ( 1 ) ---------------------------------------------MINCVTSLFQELVFLGTASGYPSPHRGASGVVLRDLCS--GSQWLFDCGEGVQIQAQKSSFVHFGKINSIFISHL**

**SjaTRZ1 ( 1 ) ------------------------------------------------------MELVFLGTASGYPSPHRGASGVVLRDLCS--GSQWLFDCGEGVQIQAQKSPFVHLGKIKCIFVSHL**

**LgiTRZ1 ( 1 ) ------------------------------------------------------MDITFLGTASAYPTPTRGVSCTVFRTD----SVCWLFDCGEGSQTQLMRSQLKAS-KIYKIFISHL**

**NveTRZ1 ( 1 ) ------------------------------------------------------MEIHFLGTGSAYPTPHRGASCVILWYN----GSCWMFDCGEGTQTQLTKSVIKSS-KITKIFISHL**

**HmaTRZ1 ( 1 ) ------------------------------------------------------MELHFLGTGSAYPSPNRSSSCLALRHN----GDIWVFDCGEGSQIQFQKSKLRSS-RISKIFITHL**

**AquTRZ1 ( 1 ) -------------------------------------------MRAGILKLSMAIELIFLGTGSAYPSPVRGASCIALRSGKNKVGGCWLFDCGEGSQTQLMRSTVRLG-RITKIFITHL**

**MbrTRZ1 ( 1 ) MRVMQIAYLWLYCCVCVCECECVCVCVVCSIFFVDAHNNASEMAAGTLYRAAQLSRLTFLGTSAGCPTKQRNVTSHVLTFSN---GRQWMLDCGEATQHQMLHCPEVKGSRLDAILITHL**

**Motif II**

**AmeTRZ1 ( 64) HGDHFFGLPGLLCTISLQSGSVV------------TKQP--IEIYGPAGLRDFIWRTMELSHTELVFPYVVH----ELVPTADQCPTEE------------LKECMYVSKTDSHP---KE**

**BtaTRZ1 ( 64) HGDHFFGLPGLLCTISLQSGSMV------------TKQP--IEIYGPVGLRDFIWRTMELSHTELVFPYVVH----ELVPTADQCPTEE------------LQESVQVDKTDNPP---KE**

**CpoTRZ1 ( 64) HGDHFFGLPGLLCTISLQSSSVV------------TKQP--IEIYGPVGLRDFIWRTMELSHTELVFPYVVH----ELVPTADQCPTEE------------LRESSHVAKADSAP---KE**

**ChoTRZ1 ( 64) HGDHFFGLPGLLCTISLQSGSAV------------TKQP--IEIYGPVGLRDFIWRIMELSHTELVFPYVVH----ELMPTEDQCPMEE------------MKEFTHVSKAGSPP---KE**

**CjaTRZ1 ( 64) HGDHFFGLPGLLCTISLQSGSIV------------SKQP--IEIYGPVGLRDFIWRTMELSHTELVFHYVVH----ELVPTVDQCPAEE------------LKEFTHVN-RAGSPS--KE**

**CfaTRZ1 ( 64) HGDHFFGLPGLLCTISLQSGSVV------------TKQP--IEIYGPAGLRDFIWRTMELSHTELVFPYVVH----ELVPTADQCPTEE------------QKEFTHVNEMDSLP---KE**

**EteTRZ1 ( 64) HGDHFFGLPGLLCTISLQSSSTG------------AKQP--VHIYGPVGLRDFLQRTMELSHTELVFPYVVH----ELVPTADQCPADE------------QAGITHEERTGCRPG---A**

**EeuTRZ1 ( 64) HGDHFFGLPGLLCTISLQSGSTV------------TKQP--IEIYGPAGLRDFIWRTMELSHTELVFPYVVH----ELVPTEDQCPPEE------------MKESVNTNRVDNSP---NE**

**EcaTRZ1 ( 64) HGDHFFGLPGLLCTISLQSGSTV------------TKQP--IEIYGPAGLRDFIWRTMELSHTELVFPYVVH----ELVPTADQCPTEE------------LKDFVHVNRADSPP---KD**

**HsaTRZ1 ( 64) HGDHFFGLPGLLCTISLQSGSMV------------SKQP--IEIYGPVGLRDFIWRTMELSHTELVFHYVVH----ELVPTADQCPAEE------------LKEFAHVN-RADSPP--KE**

**LafTRZ1 ( 65) HGDHFFGLPGLLCTISLQSGSMV------------TKQP--LEIYGPVGLRDFIWQTMELSQTELVFPYVVH----ELVPTADQCPTEE------------QKEFTHVK-RADSPP--KE**

**LgoTRZ1 ( 65) HGDHFFGLPGLLCTISLQSGSVV------------SKQP--IEIYGPVGLRDFIWRTMELSHTELVFHYVVH----ELVPTADQCPAEE------------LKEFAHVN-RADSPP--KE**

**OcuTRZ1 ( 64) HGDHIFGLPGLLCTISLQSSCTV------------SRQP--IEIFGPVGLRDFIWRTMELSHTELVFPYVVH----ELVPTADQCPTEA------------LKELAQVHPADGPP---KE**

**OprTRZ1 ( 64) HGDHLFGLPGLLCTISLQSGPTV------------PKQP--IEIFGPVGLRDFIWRTMQLSHTELVFPYVVH----ELVPTADQCPAEA------------LRELAGMDKADSPA---AQ**

**OgaTRZ1 ( 64) HGDHFFGLPGLLCTISLQSSSVV------------AKQP--IEIYGPIGLRDFIWRTMELSHTELVFPYVVH----ELVPTADQCPTEE------------LKEFARVN-VADGPP--TE**

**RnoTRZ1 ( 64) HGDHFFGLPGLLCTISLQSGSVV------------SRQP--IEIYGPVGLRDFIWRTMELSHTELVFPYVVH----ELVPTADQCPVEE------------LRESAQMDEADSSPK----**

**MeuTRZ1 ( 64) HGDHFFGLPGLLCTISLQSSPSA------------SKPP--MEIYGPVGLRNFIWKTMELSRSELTFPYVVH----ELVPTEDQCPAGE------------LKDFSYLDGTDSPP---KE**

**MmuTRZ1 ( 64) HGDHFFGLPGLLCTISLQSGSVV------------ARQP--IEIYGPVGLRDFIWRTMELSHTELVFPYVVH----ELVPTADQCPVEE------------LREFAHMDETDSSPK----**

**NleTRZ1 ( 64) HGDHFFGLPGLLCTISLQSGSMV------------SKQP--IEIYGPVGLRDFIWRTMELSHTELVFHYVVH----ELVPTADQCPAEE------------LKGFAHVN-RADSPP--KE**

**PtrTRZ1 ( 64) HGDHFFGLPGLLCTISLQSGSMV------------SKQP--IEIYGPVGLRDFIWRTMELSHTELVFHYVVH----ELVPTADQCPAEE------------LKEFAHVN-RADSPP--KE**

**SarTRZ1 ( 64) HGDHFFGLPGLLCTISLQSGSAG------------AKQP--IEIYGPAGLRDFIRKTMALSHTELVFPYVVH----ELVPTADQCPAEE------------LKQWAPESGADTPPTE---**

**StrTRZ1 ( 64) HGDHFFGLPGLLCTISLQSGSVV------------TKQP--IEIYGPVGLRDFIWRTMELSHTELVFPYVVH----ELVPTEDQCPTEV------------LKESSHVDKATESPPSPKE**

**TsyTRZ1 ( 64) HGDHLFGLPGLLCTISLQSGSMV------------SRQP--LEIYGPVGLRDFIWRTMALSHTELVFPYVVH----ELVPTTDQCPAEE------------SQEFALLNNRAESPP--KE**

**AcaTRZ1 ( 64) HGDHFFGLPGLLCTISLQSDPAG------------NKLP--VDIYGPLGLRSFLWRIMALSHSQLTFPYVVH----ELVPTPEQCPEEE------------RKELSFVDRDAALP---EG**

**XtrTRZ1 ( 64) HGDHMFGLPGFLCTVSLQCGCTP------------SKQL--VDIYGPVGLREFVQRSLEISHSQLVFPYVVH----ELVPSPDQCPAEE------------FKDFSSRGGESCLATE---**

**DreTRZ1 ( 64) HGDHLFGLPGLLCTISLNLNPQP------------DQPPPCVDIYGPRGLRLFIRVALQLSGSQLLFPYAVH----ELQPSDEQCPEEG--------------RLSPALTTASETLHPQE**

**GacTRZ1 ( 64) HGDHLFGLPGLLCTVSLNTNPE-------------QQHLSCVHIYGPRGLRHFLRVTLGLTGSQLLFPYAVH----ELEPTPDQSPEEG--------------QLSAEMTAECGPLHPQE**

**OlaTRZ1 ( 64) HGDHLFGLPGLLCTVSLNSSAES------------QQNLKCVDIYGPRGLRHFLRVTLGLTGSQLLFPYAVH----ELEPTPDQCPQEG---------LLSIEPLFHQLATERGPLHPQE**

**SsaTRZ1 ( 64) HGDHLFGLPGLLCTVSLNLNPCP------------TQPPTCVDIYGPQGLRQFLRVALELTSSQLLFPYSVH----ELEPTTDQCPTEG--------------QLSPDVTADSGRLHPQE**

**TruTRZ1 ( 64) HGDHLFGLPGLLCTVSLNSNPDP------------QQRLGRVDIYGPRGLRHFLRVTLGLTGSQLLFPYAVH----ELEPTSDQSPEE-------G-------QLSPEVTAD-GPLHPQE**

**TniTRZ1 ( 64) HGDHLFGLPGLLCTVSLNSNPDP------------QQSLSRVDIYGPRGLRHFLRVTLGLTGSQLLFPYAGKSEGQNLVPVNISVMAKRFFSNIPSFFCFVTLPPSLQVTADRGPLHPQE**

**BflTRZ1 ( 64) HGDHLFGLQGFMCTLSLNLTSQQTS---------QTPSTPSVEIYGPVGLRRYLRACLELSRSPISFSYVVH----ELEPVEEQLPGDWEDWHP----------EHTS----CGPLHPGE**

**CinTRZ1 ( 63) HGDHMFGLPGLLCTISMALKPTEDGNLDTIFSEKTVSNLKLVEIYGPLGLRRFLREALNLSRSVLTFKYVVH----EIVPNRIQYDGWKPG-------DPDWNMWDPEHTAKGCLHFCEE**

**CsaTRZ1 ( 63) HGDHMFGLPGLLCTISMAFKPNDDANSDAILPEETSPTRKCVEIFGPIGLRRFLREALNLSRSVLSFKYVVH----ELVPNNVQYNDIKVG-------DPDWNSWSPEHTAKGSLHFCEE**

**OdiTRZ1 ( 65) HGDHCFGLPDTGSLLEAVKKVKY--------------EYPTIEVYGPVGLRRYLRVALQLSRSRLACNYIVH----ELVPTQAQIGGSHPS-----------FDSWNPPANDDFERYYNE**

**SkoTRZ1 ( 62) HGDHLFGLPGFLCTIGQ-------S---------GAERDTPLELYGPIGLRRYIRTSLELSRSMLGFDYIVN----ELAVPEKELPADWESWQP----------VHDS----TGALHPNE**

**SpuTRZ1 ( 62) HGDHSFGLPGLMCTLSAAFMDP-------------DREGSIMHIYGPQGLRKYVRVSLELSRSELSYSYVVH----ELQVPDTEFPHDWETWKP----------SHEA----EGSLHPNE**

**ShaTRZ1 ( 65) HGDHSFGLPGLLCTVSQN-----------------SLRTEPLEIYGPVGLRHFVCTNLALSRADVCFDFIVH----ELETIPEQQPLDAEEWPL----------QVPS----SVPFHPCE**

**SmaTRZ1 ( 74) HGDHMFGLPGFLCTIDQKGEANNVILED----SNPQNSSASINIYGPKGLRRFLRLALALSRANLSYTYAVH----ELILEEKHCPPNWQD------------WACLEADITRDPPLFCE**

**SjaTRZ1 ( 65) HGDHMFGLPGLLCTIDQKGEANYIISGN----ESPHNDGASVNIYGPKGLRRFLRLALALSRANLSYTYAVH----ELILNEKHCPSDWHD------------WACLEADVTRDPPLFCE**

**LgiTRZ1 ( 62) HGDHVFGLPGLLCTISQN-----------------KRRNKPVDIYGPVGLRKLLRINLQLSRTQLEFDYTVH----ELQTLDHEYPADWQSWPV----------NHIS----DEDLHPNE**

**NveTRZ1 ( 62) HGDHVFGLPGLLCTIGLT----------------APDENKHVDIYGPVGLRKYLRTSLDLCQSILGYSYAIH----EIHTADSTSSQVTAEVD---------------------LLHPDE**

**HmaTRZ1 ( 62) HGDHLFGLPGLLCTIGFN----------------QNDTIDHVDIYGPIGLKNFVRASLLLSCSLLSFSYSVH----EIMIPSFQGVSNNFDSKV----------LIPDISKVELPLHPNE**

**AquTRZ1 ( 77) HGDHLFGLPGLLCTINQN----------------ADRCHIPVDIYGPYGLCHYLRTTLSLSRSFLAFKYRVH----ELLTDTNPSDIDGIVRNE----------NWNPHYVPDSVLHPNE**

**MbrTRZ1 (118) HADHVLGLPGLLATLSMAGG---------------RSESQPLKVIGPTGLRRLIETILELTFSYLTFPIDYH----ELDPLETHELGP--------------------------------**

**Flexible arm**

**Motif III GP Motif Motif IV**

**AmeTRZ1 (151) GQGRTILLDS--EENSYLLVDDEQ--FVVKAFRLFHRIPSFGFSVVEKKRPGKLNAQKLKDLGVPPGPAYGKLKNGISVVLENGVTISPQDVLKKPIVGRK--ICILGDCSGVVG-----**

**BtaTRZ1 (151) GEGRTILLDS--EENSYLLVDDEQ--FVVKAFRLFHRIPSFGFSVVEKKRPGKLNAQKLKDLGVPPGPAYGKLKNGISVVLENGVTISPQDVLKKPIVGRK--ICILGDCSGVVD-----**

**CpoTRZ1 (151) GQGRTILLDS--EENSYLLVDDEQ--FVVKAFRLFHRIPSFGFSVVEKKRPGKLNAQKLKDLGVPPGPAYGKLKNGISVVLENGITISPQDVLKKPIVGRK--ICILGDCSGVVG-----**

**ChoTRZ1 (151) GQGRTILLDS--EEKSYLLVDDEQ--FVVKAFRLFHRIPSFGFSVMEKKRPGKLNAQKLKDLGVPPGPAYGKLKNGISVVLENGVTISPQDVLKKPIIGRK--ICILGDCSGVVG-----**

**CjaTRZ1 (151) EQGRTILLDS--EENSYLLFDDEQ--FVVKAFRLFHRIPSFGFSVLEKKRPGKLNAQKLKDLGVPPGPAYGKLKNGISVVLENGVTVSPQDVLKKPIVGRK--ICILGDCSGVVG-----**

**CfaTRZ1 (151) GQGRTILLDS--EENSYLLVDDEQ--FVVKAFRLFHRIPSFGFSVVEKKRPGKLNAQKLKDLGVPPGPAYGKLKNGISVVLDNGVTISPQDVLKKPIVGRK--ICILGDCSGVVG-----**

**EteTRZ1 (151) DQGRTIHLDT--EEDAYLLVDEEQ--FVVKAFRLFHRIPSFGFSVIEK-RPGKLNAQKLKDLGVPPGPAYGKLKNGISVVLENGVTISPQDVLKKP**VVGRK**--ICILGDCAGVVG-----**

**EeuTRZ1 (151) EQGRTILLDS--EENSYLLVDDEQ--FVVKAFRLFHRIPSFGFSVVEKKRPGKLNAQKLKDLGVPPGPAYGKLKNGISVVLENGVTISPQDVLKKPIVGRK--ICILGDCSGIVG-----**

**EcaTRZ1 (151) GQGRTILLDS--EENSYLLVDDEQ--FVVKAFRLFHRIPSFGFSVMEKKRPGKLNAQKLKDLGVPPGPAYGKLKNGISVVLENGVTISPQDVLKKPIVGRK--ICILGDCSGVVG-----**

**HsaTRZ1 (151) EQGRTILLDS--EENSYLLFDDEQ--FVVKAFRLFHRIPSFGFSVVEKKRPGKLNAQKLKDLGVPPGPAYGKLKNGISVVLENGVTISPQDVLKKPIVGRK--ICILGDCSGVVG-----**

**LafTRZ1 (152) GQGRTILLDS--EENSYLLVDDEQ--FVVKAFRLFHRIPSFGFSVMEKKRPGKLNAQKLKDLGVPPGPAYGKLKNGISVVLENGVTVFPQDVLKTPIVGRK--ICILGDCSGVVG-----**

**LgoTRZ1 (152) EQGRTILLDS--EENSYLLFDDEQ--FVVKAFRLFHRIPSFGFSVMEKKRPGKLNAQKLKDLGVPPGPAYGKLKNGISVVLENGVTISPQDVLKKPIVGRK--ICILGDCSGVVG-----**

**OcuTRZ1 (151) GRGRTILLDP--EEDSYLLVDDEQ--FVVKAFRLFHRIPAFGFSVMEKKRPGKLNAQKLKDLGVPPGPAYGKLKNGISIVLENGVTISPQEVLKKPVSGRK--ICILGDCSGVVG-----**

**OprTRZ1 (151) GQGRTILLDS--EEDAYLLVDEEQ--FVVKAFRLFHRIPAFGFSVMEKKGPGKLNAQKLKDLGVPPGPAYGKLKNGISVVLENGVTVSPQDVLKKPVSGRK--ICILGDCSGVVG-----**

**OgaTRZ1 (151) GQGRTILLDS--EENSYLLVDDEQ--FVVKAFRLFHRIPSFGFSVMEKKRPGKLNAQKLKDLGVPPGPAYGKLKNGISVVLDNGVTVFPEDVLKKPIVGRK--ICILGDCSGVVG-----**

**RnoTRZ1 (150) GQGRTILLDA--EENSYCLVDDEQ--FVVKAFRLFHRIPSFGFSVVEKKRTGKLSAQKLRDLGVPPGPAYGKLKNGISVVLDNGVTISPQDVLKKPMVGRK--VCILGDCSGVVG-----**

**MeuTRZ1 (151) IQGRTILLDS--VEGSYVLVNDEQ--FLVKAFRLFHRIPSFGFSVAEKERPGRLNAQKLKDLGVPPGPAYGKLKNGVTVVLENGVKVSPQDVLEKPVPGRK--ICILGDCSGVVG-----**

**MmuTRZ1 (150) GQGRTILLDA--EENSYCLVDDEQ--FVVKAFRLFHRIPSFGFSVVEKKRAGKLNAQKLRDLGVPPGPAYGKLKNGISVVLDNGVTISPQDVLKKPMVGRK--VCILGDCSGVVG-----**

**NleTRZ1 (151) EQGRTILLDS--EENSYLLFDDEQ--FVVKAFRLFHRIPSFGFSVVEKKRPGKLNAQKLKDLGVPPGPAYGKLKNGISVVLENGVTISPQDVLKKPIVGRK--ICILGDCSGVVG-----**

**PtrTRZ1 (151) EQGRTILLDS--EENSYLLFDDEQ--FVVKAFRLFHRIPSFGFSVVEKKRPGKLNAQKLKDLGVPPGPAYGKLKNGISVVLENGVTISPQDVLKKPIVGRK--ICILGDCSGVVG-----**

**SarTRZ1 (151) GQGRTILLDE--EENAYLLVDDEQ--FVVKAFRLFHRIPSFGFSVVEKKRAGKLNAQKLRDLGVPPGPAYGKLKNGISVVLENGVTISPQDVLKKPVVGRK--ICILGDCSGVVG-----**

**StrTRZ1 (154) GQGRTILLDS--EENSYLLVDDEQ--FVVKAFRLFHRIPSFGFSVVEKKRPGKLNAQKLKDLGVPPGPAYGKLKNGISVVLENGVTISPQDVLKKPIVGRK--ICILGDCSGVVG-----**

**TsyTRZ1 (152) GQGRTILLDS--EESSYLLVDDEQ--FVVKAFRLFHRIPSFGFSVIEKKRPGKLNAQRLKDLGVPPGPAYGKLKNGISVVLENGVTIYPQDVLKKPIVGRK--ICILGDCSGVVG-----**

**AcaTRZ1 (151) TPGRTVYLDP--TDNSYPLVDNEQ--FVMKAFRLFHRIPSFGFLVEEKPRPGRLNVQKLKELGVQPGPMYGKLKEGITVVLENGTTISPSDVLEEPLPGRK--ICILGDCCSMVG-----**

**XtrTRZ1 (151) EQGRTIYASP--EDGSYELIADEQ--FTVKAFKLFHRIPSFGFVIEERERPGKLNMQKLKELGIQPGPVCGKLKLGATITLDNGQKVSPSDVLGDPIPGRK--VCILGDCSGVIA-----**

**DreTRZ1 (154) RPGRTVHPDP--DTDCYHLIDDKQ--FVVKAFKLFHRVPSFGFSVEERERPGRLNTDLLKELGLKPGPLYGRLKNGQSVTLDSGRVLTAGEVLEPPLKGRK--VCVFGDCSAPLG-----**

**GacTRZ1 (153) QPGRTIFLDV--SSDSYLLFEDNK--FSVKAFRLFHRIPSFGFCVQERDRPGRLRTELLKELGLKPGPLYGRLKAGQPVTLASGRVLLSGEALEEAVPGRK--VCVFGDCSGAVG-----**

**OlaTRZ1 (159) QPGRTICLDA--ESDSYLLFEDKK--FVVKAFRLFHRIPSFGFCIQEHDRPGRLKTELLKELGMKPGPLFGRLKAGETVTLENGRVVLPSEVLEKAISGRK--VCILGDCSSVLG-----**

**SsaTRZ1 (154) RAGRTIPLDV--NSDCYVILEEKR--FVVKAFRLFHRVPSFGFSVQEHDWPGRLNTELLKDLGLKPGPLYGRLKAGESVTLENGQVVKPSEVLEEAIPGRK--VCVLGDCSSLLG-----**

**TruTRZ1 (153) QPGRTISLDV--SSECYLLFEDKR--FVVQAFRLFHRIPSFGFCVQEHDRTGRLKTELLKELGLKPGPLYGRLKAGEAVTLESGRLVLPAEVLEDATPGRK--VCILGDCSSVLG-----**

**TniTRZ1 (172) QPGRTISLDV--SSECYLLFDDQR--FVVKAFRLFHRVPSFGFCVQEHHRPGRLKTELLKELGLKPGPVYGRLKAGKAVTLEDGRVVLPSEVLEDPIPGRK--VCILGDCSSVLG-----**

**BflTRZ1 (157) GEGRTIRRD---ETGAWHLFEDSR--FVVKAGAIYHRIPCFGFVIQEKTLPGKLDAAKLKELGVPPGPLYSRIKQGETVTLDNGQVIDPKDVVGPSRPGR--KVVILGDTHDPS------**

**CinTRZ1 (172) GFGHAIFYDE--VMKCWPLCSNKN--MTVVAGGLKHRIPSFGFVFTECDKPGKLNNAKLIEYGVPKGPLFGRIKNGEEITLEDGRILKPEDFVGPVQSGKK--VAVLQDTCDSW------**

**CsaTRZ1 (172) GYGRAIYYDH--ALKSWPLCSNKN--LSAAAGQLKHRIPSFGFVFKESAKPGKLNCAKLIEFGVPKGPLFGRIKNGEVVTLEDGRCVDPADFVGPVQLGKT--VAILQDTCDSS------**

**OdiTRZ1 (156) LPGRQIYPTDYHGQNIWKLCKDSNN-VTVTAVELLHHAPSFGYVFEEPSIIGLLDKERLEHLGLRNSPLCGKLARG-NEVEHNGRIIKPCDVQRPEVKGRK--VAVLGDSRDSN------**

**SkoTRZ1 (148) KPGRLIEPD---EHGVWHCYGDKR--ITVKAAPLTHRVPTFGFVIEEATLPGKLDSKKLLDLGVPPGPLYGRIKNGENMTLDNGDVLTPQDVLGPQRLGR--KIVILGDTSCSE------**

**SpuTRZ1 (151) KTGQAIFMT---AEGTYKLHEDDN--FIIHAAAITHRVPCFGFVFLEKPQPGRLDAEMLKARGIKPGPLYARIKAGQEIEAPDGSVIKPEDVVGPTRPGRKIGNIILGDTKESS------**

**ShaTRZ1 (150) REGRLIRPD---DNLIWHLYSDPK--LTVKAVWVKHRVPSFTFVVQESPKSGKLDASKLKAMGVPPGPLYAKLKAGQTISLENGQMVSPSEVVGPSIPGR--TVVVSGDSCDSS------**

**SmaTRZ1 (174) RSGRDIFANN--DGFWYNITNNEENCEMVHAMSIKHTIPSVGWLIIKPDQSPSLCVETAIKLGVPKGRLMGELKRG-NTIVIDGKTVRPEDVLKPRLRGHR--IAIMGDTYDSSELLRLM**

**SjaTRZ1 (165) RPGRDIYANY--DGFWYNITNNEENGEIVYAVSIKHTIPSLGWLIIKPDRPPSLCAETAMKLGVPVGRLMGELKQG-KTVVVDGKTIQPEDVLKPRLRGHR--IAIMGDTYDSSELLRLM**

**LgiTRZ1 (147) TPGTTIVPD---SNNILHLFEDDE--FIVTSVWTKHRIPSYGFIIEEKSRPGTLDVSILVSKGVPIGPLYGKIKSGETITLENGTVIKPEDVVGPPIKGR--KVIIMGDSTDSW------**

**NveTRZ1 (141) NMGREIHEN---PSGVWEICKDGD--LQVFAAPLKHRVTCFGYVVEEKDQPGALDAALLKSKGVPPGPLYAKIKNGESVTTSDGVVVDPKDVLGPSRPGRK--VVILGDTCDSS------**

**HmaTRZ1 (152) TKGKEIQFE---YDGYFDVCQTTF--LTVIAVPLKHSIPCIGYVLKEKDLPGKLNTLFLNSKGVPAGPLFGKLKMGEPVTLANGDIILPEQCLGPAKKGRK--IVILGDTCNSD------**

**AquTRZ1 (167) MQPCQIRPN---ASGVWEVCISGP--LKVLAGHLKHRVTTFGYVTIEDDIPGKLQVERLKELGIPPGPIYGKLKKGETVITPSGNTITPSDVIGPSEKGR-----KIGDTCDSY------**

**MbrTRZ1 (187) -----MF------------GPDTP---ELAAYPIKHAVPCLGYVITESPPTPKIDAARAKALGVS-GPLLGTLRRE-GSVTIEGQLVHLEDVCHAALPPRK--VVLLGDTSDASS-----**

**HEAT HST Motif V**

**AmeTRZ1 (260) ----DGGMKLCFEADLLIHEATLDDAQMDKAKEHGHSTPQMAAAFAKLCQAKRLVLTHFSQRYKPVALAREGETD---------------------GIVELKKQAESVLDLQ----EVTL**

**BtaTRZ1 (260) ----DAGVKLCFEADLLIHEATLDDTQMDKAKEHGHSTPQMAATFAKLCQAKRLVLTHFSQRYKPVALAREGEAD---------------------GIVELKKQAESVLDLQ----EVTL**

**CpoTRZ1 (260) ----DGGVKLCSDADLLIHEATLDDAQMDKAKEHGHSTPQMAATFAKLCRAKRLILTHFSQRYKPVALAREGEAD---------------------GIAELKKQAESVLDLQ----EVTL**

**ChoTRZ1 (260) ----DGGVKLCFEADLLIHEATLDDTQMDKAKEHGHSTPQMAAAFAKLCQAKRLVLTHFSQRYKPVALAKEGETD---------------------GIAELKKQAESVLDLQ----EVIL**

**CjaTRZ1 (260) ----DEGVKLCFEADLLIHEATLDDAQMDKAKEHGHSTPHMAAMFAKMCRAKRLVLTHFSQRYKPVALAREGETD---------------------GTAELKKQAESVLDLQ----EVTL**

**CfaTRZ1 (260) ----DGGVKLCFEADLLIHEATLDDAQMDKAKEHGHSTPQMAAAFAKLCQAKRLVLTHFSQRYKPVALAREGEAD---------------------GIVELKKQAESVLDLQ----EVTL**

**EteTRZ1 (259) ----DGGVELCLEADLLIHEATLDDTQMDKAKERGHSTPQMAAAFANRCQAKRLVLTHFSQRYKPVTLAKEGESD---------------------GTAELKKQAESMFEHQ----EVTL**

**EeuTRZ1 (260) ----DGAVKLCFEADLLIHEATLDDTQMDKAKEHGHSTPQMAAEFAKLCQAKRLVLTHFSQRYKPVALAREGETD---------------------GIVELKKQAESVLDLQ----EVTL**

**EcaTRZ1 (260) ----DGGVKLCFEADLLIHEATLDDAQMDKAKEHGHSTPQMAAAFAKLCQAKRLVLTHFSQRYKPVALAREGETD---------------------GIVELKKQAESVLDLQ----EVTL**

**HsaTRZ1 (260) ----DGGVKLCFEADLLIHEATLDDAQMDKAKEHGHSTPQMAATFAKLCRAKRLVLTHFSQRYKPVALAREGETD---------------------GIAELKKQAESVLDLQ----EVTL**

**LafTRZ1 (261) ----DGGVKLCSEADLLIHEATLDDTQMDKAKEHGHSTPQMAATFAKLCQAKRLVLTHFSQRYKPVALAREGETD---------------------GIAELKKQAESVLDLQ----EVTL**

**LgoTRZ1 (261) ----DGGVKLCFEADLLIHEATLDDAQMDKAKEHGHSTPQMAATFAKLCRAKRLVLTHFSQRYKPVALAREGETD---------------------GIAELKKQAESVLDLQ----EVTL**

**OcuTRZ1 (260) ----DGGVKLCFEADLLIHEATLDDAQMDKAKEHGHSTPWMAATFAKLCHAKRLVLTHFSQRYKPAALAKEGEAD---------------------SIVELKRQAESVSDRQ----EVTL**

**OprTRZ1 (260) ----EGGVKLCFEADLLIHEATLNDAQMDKAKEHGHSTPQMAATFAKLCCAKRLVLTHFSQRYKPTSLAREGEPD---------------------GTMELKRQAEAVLELQ----EVTL**

**OgaTRZ1 (260) ----DGGVKLCFEADLLIHEATLDDAQMDKAKEHGHSTPQMAATFAKSCHAKRLVLTHFSQRYKPVALAREGETD---------------------GIAELKKQAESVLDLQ----EVTL**

**RnoTRZ1 (259) ----DGGVKLCFEADLLIHEATLDDSQMDKAKEHGHSTPQMAATFAKLCRAKRLVLTHFSQRYKPTALAREGEAD---------------------GITELKKQAESVLELQ----EVTL**

**MeuTRZ1 (260) ----DEGVKLCFEADLLVHEATLDDTHMDKAKEYGHSTPRMAAEFAKLCKVKRLVLTHFSQRYKPPLLSGEGETD---------------------DIRELKKQAESVLDFQ----EVTL**

**MmuTRZ1 (259) ----DGGVKLCFEADLLIHEATLDDSQMDKAREHGHSTPQMAAAFAKLCRAKRLVLTHFSQRYKPTALAREGEAD---------------------GIAELRKQAEAVLELQ----EVTL**

**NleTRZ1 (260) ----DGGIKLCFEADLLIHEATLDDAQMDKAKEHGHSTPQMAATFAKLCHAKRLVLTHFSQRYKPVALAREGETD---------------------GIAELKKQAESVLDLQ----EVTL**

**PtrTRZ1 (260) ----DGGVKLCLEADLLIHEATLDDAQMDKAKEHGHSTPQMAATFAKLCRAKRLVLTHFSQRYKPVALAREGETD---------------------GIAELKKQAESVLDLQ----EVTL**

**SarTRZ1 (260) ----DGGVRLCFEADLLIHEATLDDSQMDKAKEHGHSTPQMAAAFAKLCQAKRLVLTHFSQRYKPVALAREGETD---------------------GTAELKKQAESVLDLQ----EVTL**

**StrTRZ1 (263) ----DGGVKLCFEADLLIHEATLDDTQMDKAKEHGHSTPQMAATFAKMCQAKRLVLTHFSQRYKPVALAREGETD---------------------GIAELKKQAESVLDLQ----EVTL**

**TsyTRZ1 (261) ----DGGVKLCFEADLLIHEATLDDAQMDKAKEHGHSTPQMAATFAKLCHAKKLVLTHFSQRYKPVALAREGETD---------------------GIAELKKQAESVLDLQ----EVTL**

**AcaTRZ1 (260) ----EGAASLCHGADILVHEATLDDSQMDKAREHGHSTPKMAAEFAKRCQVKRLVLSHLSQRYKPTSQVGAGDLD----------------------VLELKRQAELALDGQ----EVVL**

**XtrTRZ1 (260) ----VGGAKLCCDADVLIHEATLDDSQMDKAKEHGHSTPKMAADFANLCCARKLVLTHFSQRYKPPGLVTEGDED----------------------VTVLKQQAESALPGQ----HVTL**

**DreTRZ1 (263) ----EGFKRACYGADVLVHEATLENGQQEKAVEHGHSTPGMAAAVALTCEAKTLVLHHFSQRYKPENLRRDDDED---------------------DVSELKRQAELVLQGSRT--DVIL**

**GacTRZ1 (262) ----DGALRLCSEADVLVHEATLGEEQREKAVDHGHSTPVMAAAVARACRARRLVLHHFSQRYKPGSLQREGDED---------------------DVSQLKRQAEEALRDSGI--EVTL**

**OlaTRZ1 (268) ----EAALRLCHGANVLVHEATLGNEHQEKAVEHGHSTPGMAAAVARACCAQRLVLYHFSQRYKPSSLLKDGDEN---------------------EVLGLKRDAEEALQGRDV--EVTL**

**SsaTRZ1 (263) ----EGPLRACHRADILVHEATLSDEHREKAVDHGHSTPSMAAAVAQACCARMLVLYHFSQRYKPAGQHKEGDED---------------------DVLELRRQAEEALQGTGI--EVSL**

**TruTRZ1 (262) ----EGPLRLCHRADILVHEATLANEHREKAVEHGHSTPEMAAAVAQACRARRLVLHHFSQRYKPCGAQKEGGKD---------------------DVSELKRQAEDALQDSGV--EVVL**

**TniTRZ1 (281) ----EKPLMLCHGADILVHEATLANEHREKAQDHGHSTPGMAAAVARACCARRLVLHHFSQRYKPGGAQKEGDKD---------------------DVSELKRQAEEALQDSGV--DVVL**

**BflTRZ1 (264) -----PLTSLAQGADVLVHEATLENSMRSKAIEVGHSTPEMAATFAKTIGAKTLILTHFSQRYRPQGATLEKGEE---------------------SVCKLQEEAAAVFGPG----DVIA**

**CinTRZ1 (280) -----HMREICNNCTLLIHEATNENCQQDRCIANGHSTPAMVAQFANAIGAQKIVLTHVSQRYKHPLTHDVEPDD---------------------QTTEILETETRTDFSG----PVIA**

**CsaTRZ1 (280) -----HMREMCQNCSLLIHEATNENTMQKKCIANGHSTPGMAASFARMVTAEKLILTHVSQRYKHHLTHEVSPDD---------------------QTTDILVNEAKLDFSG----PIIA**

**OdiTRZ1 (266) -----LASNYCHAADLIMHEATLENDMESTAIEHGHSTPRMAVEFARRCGAKKLVLNHFSQRYRSEKDEKYNLEES------------------ITDQILLKQAIQCALELGESENFVGI**

**SkoTRZ1 (255) -----QITSVAMDTDILVHEATLENELLDKCIENGHSTPGMAAEFANKINAKKLILTHFSQRYRAVSDTLKEGEE---------------------SVGKLIKQAELVFSRG----DVVA**

**SpuTRZ1 (260) -----RIIPIAMNADLLVHEATLADENHDLCTGRGHSTPAMAGQFAAQIQCEHLVITHFSQRYYPLSHQGKEGDE---------------------TVQKLLDQTRATFPGG----KVTA**

**ShaTRZ1 (257) -----ELNKVARGATVLVHEATLENSLWEQCVQNGHSTPEMTATLAKELDVKMLVITHVSQRYKTRAAELKEGDR---------------------SVQILLDEALEILPAD----KVIL**

**SmaTRZ1 (289) QVLHTANQITSITLDTLVHEATLDDSLYEDALSKGHSVPSVVMKLACQLNVRQLVLTHFSHRYDRIDSSDENTIKNKIGEQAIYSNKSKSDKKNKPSLQIILDQAKSTDFSG----DIIL**

**SjaTRZ1 (280) QVLHTANHITSITLDTLVHEATLDDSLYEDALNKGHSVPSTVAKLACQLNVRQLVLTHFSHRYDRIESSIENAVKSEVDEQVNCSKKSKSDKKSKPSLQIILDQARATDFTG----EIIL**

**LgiTRZ1 (254) -----NLKHIGQDADVLIHEATLENSLSEKCIENGHSTPEMVAKLAKSLNVKQLILTHFSQRYKPLSCQLKEGDD---------------------SVQTLLNEAEAILGTG----SVIC**

**NveTRZ1 (248) -----AIENLAYKADCVIHEATLEDEMKEKAIENGHSTPGMAGAFAKTIQAKQLVLTHFSQRYKDSDSVAKH----------------------------VQQATHAFSC-D----RVLA**

**HmaTRZ1 (259) -----EIIPFAIDADILVHEATNENADEKKSVEHGHSTAGMAGRFAALVRARTLIITHFSQRYKTISDESEN--D---------------------SIQKLKKEAEENFN-G----QVIA**

**AquTRZ1 (271) -----RIAEIAKNADVLVHEATNENSHFEKCVENGHSTPSMAAEFALSIGARKLILTHFSQRYKDLNEELEENEE---------------------SVEKLLKEAQEIFG-N----EVYV**

**MbrTRZ1 (278) ------LHAAGQGCHTVVHEATLASGMEDQAVANGHSTTNMAVDTALALGASRLILTHISPRYAVASPEETTPAKS--------------------TDLVLLDEARNAATARSSALEVEL**

**AxDx**

**AmeTRZ1 (351) AEDFMVIGIPMKK----------**

**BtaTRZ1 (351) AEDFMVISIPIKK----------**

**CpoTRZ1 (351) AEDFMVIGIPIKK----------**

**ChoTRZ1 (351) AEDFMVIGIPVKK----------**

**CjaTRZ1 (351) AEDFMVISIPMKK----------**

**CfaTRZ1 (351) AEDFMVNKFRSRSETIFLMRLTC**

**EteTRZ1 (350) AEDFMVISLPIKK----------**

**EeuTRZ1 (351) AEDFLVIGIPIKK----------**

**EcaTRZ1 (351) AEDFMVIGIPIKK----------**

**HsaTRZ1 (351) AEDFMVISIPIKK----------**

**LafTRZ1 (352) AEDFMVIGIPIKK----------**

**LgoTRZ1 (352) AEDFMVISIPIKK----------**

**OcuTRZ1 (351) AEDFMVISVPMKK----------**

**OprTRZ1 (351) AEDFMVISIPIKK----------**

**OgaTRZ1 (351) AEDFMVIGIPIKK----------**

**RnoTRZ1 (350) AEDFMVIGIPIKK----------**

**MeuTRZ1 (351) AEDFMVIDIPKKKT---------**

**MmuTRZ1 (350) AEDFMVIGIPIKK----------**

**NleTRZ1 (351) AEDFMVISIPIKK----------**

**PtrTRZ1 (351) AEDFMVISIPIKK----------**

**SarTRZ1 (351) AEDFMVIGIPIKK----------**

**StrTRZ1 (354) AEDFMVIGIPIKK----------**

**TsyTRZ1 (352) AEDFMVIGIPIKK----------**

**AcaTRZ1 (350) AEDFMTIAVPLKKTR--------**

**XtrTRZ1 (350) AEDFMTITIPMKKQ---------**

**DreTRZ1 (356) AEDFLTLPVVLKRNSTT------**

**GacTRZ1 (355) AEDFLTLPVPLRR----------**

**OlaTRZ1 (361) AEDFMTLPVPLRR----------**

**SsaTRZ1 (356) AEDFLTIPIPLIRLH--------**

**TruTRZ1 (355) AEDFLTLNVPIRCDQSLPVR---**

**TniTRZ1 (374) AEDFLTLSVPIRT----------**

**BflTRZ1 (354) AQDLQTVHILQHNS---------**

**CinTRZ1 (370) AYDGLVVNVNKKVF---------**

**CsaTRZ1 (370) AYDGLVVNVNKGTF---------**

**OdiTRZ1 (363) ARDLKVIDVIRAK----------**

**SkoTRZ1 (345) ADDLMQIVIPQH-----------**

**SpuTRZ1 (350) AEDFLTISVPARR----------**

**ShaTRZ1 (347) AEDFAVYTVPQQPPGGSASNMT-**

**SmaTRZ1 (405) ADDQLLVKIPPVTQ---------**

**SjaTRZ1 (396) ADDQLLVKIPPVTHNHNIGV---**

**LgiTRZ1 (344) AEDFMVYNVARNK----------**

**NveTRZ1 (330) ASDFSKVVIPLQK----------**

**HmaTRZ1 (346) ADDLLFIPVLFCDS---------**

**AquTRZ1 (360) ASDFKTFNVPLHRN---------**

**MbrTRZ1 (372) ARDFATFSF--------------**
