# Supplementary material for: Identification and Sequence Analysis of Metazoan tRNA 3′-End Processing Enzymes tRNase Zs
Source: PLoS One. 2012 Sep 4;7(9):e44264. doi: 10.1371/journal.pone.0044264 (PMC3433465; doi:10.1371/journal.pone.0044264)
Supplement: Figure S3 — Comparison of the sequences flanking the two in-frame AUG start codons in 58 metazoan tRNase ZL mRNAs. Sequences were aligned by the start codon. The AUG start codons are indicated in red. In each sequence, nucleotides at positions −3 and +4, which are the most important determinants of context are shown in blue and green, respectively. Although the consensus sequences surrounding AUG start codons vary considerably between eukaryotic groups, they share a strong preference for purines at the position −3 and G at the position +4 for translational initiation. (DOC) [file pone.0044264.s003.doc]

Figure S3：Comparison of the sequences flanking the two in-frame AUG start codons in 58 metazoan tRNase Z^L^ mRNAs

*H. sapiens* -15 GGTGGAGACGGG**C**GC**ATGT**GGGCG // GCGGCCGGACGC**A**CC**ATGT**CGCAG +54

*P. troglodytes* -15 GGTGGAGACGGG**C**GC**ATGT**GGGCG // GCGGCCGGACGC**A**CC**ATGT**CGCAG +54

*L. gorilla* -15 GGTGGAGACGGG**C**GC**ATGT**GGGCG // GCGGCCGGACGC**A**CC**ATGT**CGCAG +54

*C. porcellus* -15 GGTGGGGACAGG**C**GC**ATGT**GGGCG // GTGGCCGGGCGC**A**CC**ATGT**CTCAG +54

*C. jacchus* -15 TGTGGAGACAGG**C**GC**ATGT**GGGCG // GCGGCTAGGCGC**A**CC**ATGT**CGCAG +54

*D. novemcinctus* -15 GGTAGAGGCGCG**C**GC**ATGT**GGGCA // GCGGCCGGGCGC**G**CC**ATGT**CGCAG +54

*D. ordii* -15 GGCTGAACGGGG**C**GC**ATGT**GGGCG // GGGGCCGGGCGC**G**CC**ATGT**CACAG +54

*E. caballus* -15 GGTGGAGGCTGG**C**GC**ATGT**GGGCT // GCGGCTGGGCGC**G**CC**ATGT**CGCAG +57

*E. europaeus* -15 GGTGGGCACTGG**C**GC**ATGT**GGACT // GCGGCCAGGCGC**A**CC**ATGT**CGCAG +54

*N. leucogenys* -15 GGTGGAGACGGG**C**GC**ATGT**GGGCG // GCGGCCGGACGC**A**CC**ATGT**CGCAG +54

*O. cuniculus* -15 TGGAGACACAGG**C**GC**ATGT**GGACG // GTGGCCGGGCGC**A**CC**ATGT**CGCAG +54

*O. garnettii* -15 GGCGGAGACGGG**C**GC**ATGT**GGGCT // GTGGCGGGGCGC**A**CG**ATGT**CACAG +54

*O. princeps* -15 TGGAGCAGCAGG**C**GC**ATGT**GGGCG // GTGGCCGGGCGC**A**CC**ATGT**CGCAG +54

*P. vampyrus* -15 GGTGGAAGCTGG**C**GC**ATGT**GGGCG // GCGGCCGGGCGC**A**CC**ATGT**CGCAG +60

*S. tridecemlineatus* -15 GGTGGAGACAGG**C**GC**ATGT**GGGCG // GTGGCCGGGCGC**G**CC**ATGT**CTCAG +54

*B. Taurus* -15 AAGAGGAGGCTG**C**GC**ATGT**GGGCA // GCGGCCGGGCGC**A**CC**ATGT**CACAG +60

*T. Syrichta* -15 GGTGGAGTCGGT**C**GC**ATGT**GGGCG // GCGGCCGGTCGC**A**CC**ATGT**CGCAG +54

*T. truncates* -15 GGGAGGAGGGGG**C**GC**ATGT**GGGCT // GCGGCCGGGCGC**G**CC**ATGT**CTCAG +60

*C. familiaris* -15 GGTGGAGGCTGG**T**GC**ATGT**GGCTC // GCGGCCGGGCGC**G**CC**ATGT**CGCAG +60

*E. telfairi* -15 TTCTGAGGAGGG**T**GC**ATGT**GGGCT // GCGGCTGGGCGC**A**CC**ATGT**CGCAG +54

*L. Africana* -15 GAGGGACGCAGG**T**GC**ATGT**GGGCT // GCGGCTGGGGGC**A**CC**ATGT**CGCAG +54

*M. musculus* -15 GGTGTGGTCGGG**T**GC**ATGT**GGGCG // CTTGGCCTGCGC**A**CC**ATGT**CGCAG +54

*R. norvegicus* -15 GGTGAGGACTGG**T**GC**ATGT**GGGCC // CTTGGCCTGCGC**A**CC**ATGT**CGCAG +54

*S. scrofa* -15 ACCAGGCGGTGG**T**GC**ATGT**GGGCG // GTGGCTGGGCGC**A**CC**ATGT**CGCAG +60

*T. belangeri* -15 GGTGGAGACCGG**T**GC**ATGT**GGACG // GCGGCCGGGCGC**G**CC**ATGT**CCCAG +54

*G. gallus* -15 GGGCGCTGCACG**C**GG**ATGT**GTAGG // GGTGCGGGCACG**G**TC**ATGG**CCGAG +66

*A. Carolinensis* -15 GTGGAGGGCTGC**T**GG**ATGT**GGCGG // GGGGCGGGCACA**A**CC**ATGT**CGGTG +54

*X. tropicalis* -15 TTCCATGCTGTC**T**GA**ATGT**GGTTA // CTGTCTCGTTTT**G**CC**ATGT**CTGAA +63

*G. aculeatus* -15 GTGTCTTTATTA**T**GA**ATGC**GGTCC // CAGTTTTTTCGA**A**CA**ATGG**CGTCT +140

*O. latipes* -15 GAGTTGGCGTTT**A**GC**ATGA**ACGCG // TGGGGTTTCCGG**A**CC**ATGG**CGTCC +141

*T. rubripes* -15 CCCGCCCGGTTT**A**TT**ATGA**TCAGG // CAGCTTTTCCGA**A**CC**ATGG**CGAGC +117

*T. Nigroviridis* -15 TGGAGGCTTCCT**A**GT**ATGA**GCCCT // CACTCTTCCCCA**A**CC**ATGT**CGAGC +114

*S. kowalevskii* -15 CACGTTAAGTAC**C**CG**ATGG**CAGTT // CACCCGAATGAG**A**AC**ATGA**GTGGG +120

*D. melanogaster* -15 TCTGACGAAATA**C**GA**ATGT**ATTTA // ACAACCAGTAAT**T**TG**ATGG**CGGCC +78

*D. erecta* -15 TACGAAGAAGTA**C**GA**ATGT**ATTTG // TCAACGAGAAAT**T**TG**ATGG**CGGCC +78

*D. sechellia* -15 TCCGACGAAATA**C**GA**ATGT**ATTTA // ACAACGAGTAAT**T**TG**ATGT**CGGCC +78

*D. simulans* -15 TCCGACGAAATA**C**GA**ATGT**ATTTA // ACAACGAGTAAT**T**TG**ATGT**CGGCC +78

*D. yakuba* -15 TCCGAAGACATA**C**GA**ATGT**ATTTA // ACAACTTGTAAT**T**TG**ATGT**CGGCA +78

*D. mojavensis* -15 GAAATGTGGTTG**C**CA**ATGT**TGAAA // AACCATTGCATC**A**GA**ATGA**GCAGC +75

*D. willistoni* -15 CTGACTCAATTG**C**CA**ATGA**TTTTT // CATCGCCTCATC**A**AC**ATGA**GTAGC +90

*D. grimshawi* -15 GCTGTTGGACGC**A**TG**ATGT**GGTTG // AAGCACAGTATC**A**GA**ATGA**GCATC +87

*D. virilis* -15 CACGGTAAACGC**G**AA**ATGT**GGCTG // GAAAATTGCATC**A**GG**ATGA**GTAGC +87

*C. remanei* -15 ACAATTTGGCAG**C**TG**ATGC**GACGA // AATCTCCGTCAA**T**CG**ATGG**CTTCT +406

*C. elegans* -15 GAAAGCTCGAAG**C**TG**ATGC**TCGGA // AATCTGCGACAA**T**CA**ATGG**CGGCA +1248

*C. Briggsae* -15 TGTCTTTATCTT**C**AG**ATGA**AGATT // AACCTGCGTCAA**T**CC**ATGG**CTTCT +571

*T. castaneum* -15 TCTCATCAAAAA**C**TA**ATGT**CTAGT // AACATTTTGAAG**A**CC**ATGC**CTAAG +89

*A. aegypti* -15 CCTCTCTCAGAC**C**TA**ATGT**AGTTG // CGTAAGTCCCCG**A**AA**ATGC**CGCTG +80

*A. echinatior* -15 AAGAAGACCGAA**C**GA**ATGA**GGTCC // CACAACAATCAG**A**TC**ATGC**CACGT +153

*A. gambiae* -15 ACCAGTCCACAA**T**AT**ATGT**ATACA // AAGCTGCTCAGC**A**AA**ATGC**CGCTC +111

*B. mori* -15 CAGAACACATCA**A**CT**ATGC**CAAAA // CAAACAGCTCGA**A**TA**ATGG**AGATA +36

*D. ananassae* -15 GTTGACTTAATA**C**GA**ATGT**TTGTG // ATTAGAACTTTC**A**AA**ATGA**GCAGC +66

*H. melpomene* -15 GTAACCTCAAAA**C**AC**ATGT**ACGGT // GAATTTCTAGGC**A**CA**ATGC**CTAAA +111

*N. Vitripennis* -15 TAGTCTTCAGCA**C**AC**ATGA**TTTTC // TTTCGACCCTTG**A**TT**ATGC**CAAAG +108

*S. Mansoni* -15 GCTTGCAGTGAG**T**GA**ATGC**TGCGT // CGCCCTGAAGAT**A**GG**ATGA**TACCA +90

*N. vectensis* -15 GTTCAAAAGTCG**A**CT**ATGA**TTGTT // CTCTCTACAACT**T**GT**ATGG**GTTTG +261

*H. magnipapillata* -15 AATATCTAATAA**A**TA**ATGT**CTCCT // TTCGACCAAGTA**A**AA**ATGT**TATCA +38

*T. adhaerens* -15 CTCATCTTTTCT**T**CA**ATGA**TATTC // AGGATATCAAAA**T**CA**ATGT**CTGCC +57

*M. brevicollis* -15 CCGCTTGGATCG**G**GC**ATGT**GGGGG // CACTCGTGCGGG**C**TG**ATGG**ATGCG +48
