# Supplementary figures and images for: Identification and Sequence Analysis of Metazoan tRNA 3′-End Processing Enzymes tRNase Zs
Source: PLoS One. 2012 Sep 4;7(9):e44264. doi: 10.1371/journal.pone.0044264 (PMC3433465; doi:10.1371/journal.pone.0044264)

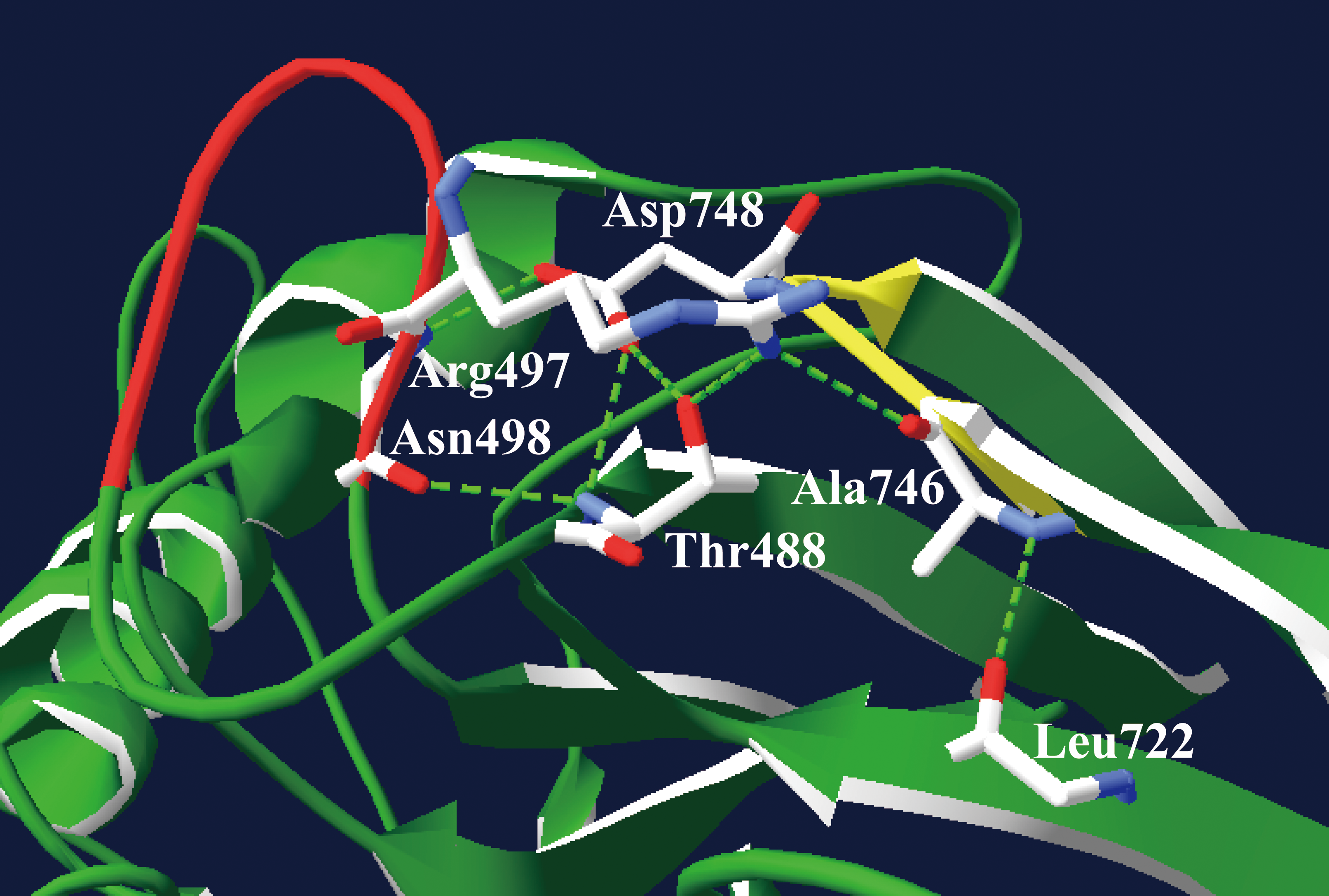

Supplement: Figure S4 — The predicted hydrogen bonding network in the AxDx strand/loop region of human tRNase ZL. The published structure of human tRNase ZS (PDB code 3ZWF) was used as a template to build a modeled structure of C-terminal region of human tRNase ZL (residues 481–754) using the homology modeling server SWISSMODEL (http://swissmodel.expasy.org/). The picture is labeled as described in the legend to Figure 8. The potential hydrogen bond network is formed by the O atom of Ala746 and the NH1 atom of Arg497 (3.07Å), the OD2 atom of Asp748 and the HN atom of Asn498 (2.78Å), the HN atom of Ala 746 and the O atom of Leu722 (2.86Å), the OD2 atom of Asp748 and the HN atom of Thr488 (3.06Å), and the OD2 atom of Asp748 and the OG1 atom of Thr488 (2.71Å), the OG1 atom of Thr488 and the NH1 atom of Arg497 (2.81Å) and the O atom of Asn498 and the HN atom of Thr488 (3.01Å). The figure was prepared using Swiss-PdbViewer [59]. (TIF) [file pone.0044264.s004.tif]
