# Supplementary material for: Identification and Sequence Analysis of Metazoan tRNA 3′-End Processing Enzymes tRNase Zs
Source: PLoS One. 2012 Sep 4;7(9):e44264. doi: 10.1371/journal.pone.0044264 (PMC3433465; doi:10.1371/journal.pone.0044264)
Supplement: Table S1 — Distribution of candidate tRNase Zs identified in metazoans. Abbreviations for species names are indicated in the parentheses. +The number of amino acids in metazoan tRNase Z and tRNase Z-like proteins. *Indicates that mispredicted sequences obtained from the databases have been corrected. ?Indicates the sequence could not be correctly predicted. (DOC) [file pone.0044264.s005.doc]

Table S1: Distribution of candidate tRNase Zs identified in metazoans

| Species | Protein name | Forms | Accession number | Database | No. aa+ |
| --- | --- | --- | --- | --- | --- |
| **Deuterostomes** |  |  |  |  |  |
| *Anolis carolinensis* | AcaTRZ1 | tRNase Z^S^ | XP_003227333.1 | NCBI | 364 |
| *Anolis carolinensis* | AcaTRZ2 | tRNase Z^L^ | XP_003217198.1 | NCBI | 834* |
| *Ailuropoda melanoleuca* | AmeTRZ1 | tRNase Z^S^ | XP_002916832.1 | NCBI | 363 |
| *Ailuropoda melanoleuca* | AmeTRZ2 | tRNase Z^L^ | XP_002930135.1 | NCBI | ? |
| *Branchiostoma floridae* | BflTRZ1 | tRNase Z^S^ | XP_002598072.1 | NCBI | 367* |
| *Branchiostoma floridae* | BflTRZ2 | tRNase Z^L^ | XP_002603583.1 | NCBI | 785* |
| *Bos taurus* | BtaTRZ1 | tRNase Z^S^ | NP_001039792.1 | NCBI | 363 |
| *Bos taurus* | BtaTRZ2 | tRNase Z^L^ | XP_872127 | NCBI | 814* |
| *Callithrix jacchus* | CjaTRZ1 | tRNase Z^S^ | XP_002757289.1 | NCBI | 363 |
| *Callithrix jacchus* | CjaTRZ2 | tRNase Z^L^ | XP_002747939.1 | NCBI | 825* |
| *Canis familiaris* | CfaTRZ1 | tRNase Z^S^ | XP_849493.1 | NCBI | 374 |
| *Canis familiaris* | CfaTRZ2 | tRNase Z^L^ | XP_546630.2 | NCBI | 821 |
| *Cavia porcellus* | CpoTRZ1 | tRNase Z^S^ | [ENSCPOP00000011325](http://www.ensembl.org/Cavia_porcellus/Transcript/ProteinSummary?db=core;g=ENSCPOG00000012588;r=scaffold_14:14505163-14512109;t=ENSCPOT00000012709) | Ensembl | 363 |
| *Cavia porcellus* | CpoTRZ2 | tRNase Z^L^ | ENSCPOP00000008171 | Ensembl | 825 |
| *Choloepus hoffmanni* | ChoTRZ1 | tRNase Z^S^ | ENSCHOP00000002534 | Ensembl | 363 |
| *Choloepus hoffmanni* | ChoTRZ2 | tRNase Z^L^ | ENSCHOP00000004181 | Ensembl | ? |
| *Ciona intestinalis* | CinTRZ1 | tRNase Z^S^ | 299192 | Metazome | 383 |
| *Ciona intestinalis* | CinTRZ2 | tRNase Z^L^ | 295609 | Metazome | 727 |
| *Ciona savignyi* | CsaTRZ1 | tRNase Z^S^ | tBlastn | Metazome | 383 |
| *Ciona savignyi* | CsaTRZ2 | tRNase Z^L^ | tBlastn | Metazome | 792* |
| *Danio rerio* | DreTRZ1 | tRNase Z^S^ | NP_001003503.1 | NCBI | 372 |
| *Danio rerio* | DreTRZ2 | tRNase Z^L^ | XP_003198113.1 | NCBI | 895 |
| *Echinops telfairi* | EteTRZ1 | tRNase Z^S^ | ENSETEP00000003177 | Ensembl | 362 |
| *Echinops telfairi* | EteTRZ2 | tRNase Z^L^ | ENSETEP00000013299 | Ensembl | ? |
| *Equus caballus* | EcaTRZ1 | tRNase Z^S^ | XP_001499894.1 | NCBI | 363 |
| *Equus caballus* | EcaTRZ2 | tRNase Z^L^ | XP_001503380.2 | NCBI | 820 |
| *Erinaceus europaeus* | EeuTRZ1 | tRNase Z^S^ | ENSEEUP00000009212 | Ensembl | 363 |
| *Erinaceus europaeu* | EeuTRZ2 | tRNase Z^L^ | ENSEEUP00000009849 | Ensembl | ? |
| *Gallus gallus* | GgaTRZ1 | tRNase Z^S^ |  |  | ? |
| *Gallus gallus* | GgaTRZ2 | tRNase Z^L^ | XP_415584.2 | NCBI | 835* |
| *Gasterosteus aculeatus* | GacTRZ1 | tRNase Z^S^ | ENSGACG00000007275 | Metazome | 367 |
| *Gasterosteus aculeatus* | GacTRZ2 | tRNase Z^L^ | ENSGACG00000014454 | Metazome | 865* |
| *Lowland gorilla* | LgoTRZ1 | tRNase Z^S^ | [ENSGGOP00000002584](http://www.ensembl.org/Gorilla_gorilla/Transcript/ProteinSummary?db=core;g=ENSGGOG00000002624;r=18:48821633-48841736;t=ENSGGOT00000002638) | Ensembl | 364 |
| *Lowland gorilla* | LgoTRZ2 | tRNase Z^L^ | Q9GL73.1 | Uniprot | 826 |
| *Loxodonta africana* | LafTRZ1 | tRNase Z^S^ | ENSLAFP00000001283 | Ensembl | 364 |
| *Loxodonta africana* | LafTRZ2 | tRNase Z^L^ | ENSLAFP00000025345 | Ensembl | 819 |
| *Homo sapiens* | HsaTRZ1 | tRNase Z^S^ | NP_061166.1 | NCBI | 363 |
| *Homo sapiens* | HsaTRZ2 | tRNase Z^L^ | NP_060597.4 | NCBI | 826 |
| *Macropus eugenii* | MeuTRZ1 | tRNase Z^S^ | ENSMEUP00000015312 | Ensembl | 364 |
| *Macropus eugenii* | MeuTRZ2 | tRNase Z^L^ | ENSMEUP00000000013 | Ensembl | ? |
| *Mus musculus* | MmuTRZ1 | tRNase Z^S^ | NP_444485.2 | NCBI | 362 |
| *Mus musculus* | MmuTRZ2 | tRNase Z^L^ | CAI24609 | NCBI | 824 |
| *Nomascus leucogenys* | NleTRZ1 | tRNase Z^S^ | ENSNLEP00000014405 | Ensembl | 363 |
| *Nomascus leucogenys* | NleTRZ2 | tRNase Z^L^ | ENSNLEP00000017789 | Ensembl | ? |
| *Ochotona princeps* | OprTRZ1 | tRNase Z^S^ | [ENSOPRP00000012382](http://www.ensembl.org/Ochotona_princeps/Transcript/ProteinSummary?db=core;g=ENSOPRG00000013590;r=GeneScaffold_518:104198-110868;t=ENSOPRT00000013570) | Ensembl | 363 |
| *Ochotona princeps* | OprTRZ2 | tRNase Z^L^ | ENSOPRP00000010201 | Ensembl | ? |
| *Oikopleura dioica* | OdiTRZ1 | tRNase Z^S^ | FN654938.1 | Uniprot | 375 |
| *Oikopleura dioica* | OdiTRZ2 | tRNase Z^L^ |  |  | ? |
| *Oryzias latipes* | OlaTRZ1 | tRNase Z^S^ | ENSORLG00000004777 | Metazome | 373 |
| *Oryzias latipes* | OlaTRZ2 | tRNase Z^L^ | ENSORLG00000001024 | Metazome | 869 |
| *Oryctolagus cuniculus* | OcuTRZ1 | tRNase Z^S^ | XP_002713584.1 | NCBI | 363 |
| *Oryctolagus cuniculus* | OcuTRZ2 | tRNase Z^L^ | XP_002718955.1 | NCBI | 824 |
| *Otolemur garnettii* | OgaTRZ1 | tRNase Z^S^ | ENSOGAP00000010807 | Ensembl | 363 |
| *Otolemur garnettii* | OgaTRZ2 | tRNase Z^L^ | ENSOGAP00000011316 | Ensembl | ? |
| *Pan troglodytes* | PtrTRZ1 | tRNase Z^S^ | XP_001155672.1 | NCBI | 363 |
| *Pan troglodytes* | PtrTRZ2 | tRNase Z^L^ | NP_001009034.1 | NCBI | 826 |
| *Rattus norvegicus* | RnoTRZ1 | tRNase Z^S^ | 62664619 | JGI | 362 |
| *Rattus norvegicus* | RnoTRZ2 | tRNase Z^L^ | NP_758829.1 | NCBI | 827 |
| *Saccoglossus kowalevskii* | SkoTRZ1 | tRNase Z^S^ | tBlastn | NCBI | 356 |
| *Saccoglossus kowalevskii* | SkoTRZ2 | tRNase Z^L^ | tBlastn | NCBI | 789* |
| *Salmo salar* | SsaTRZ1 | tRNase Z^S^ | EST | NCBI | 370 |
| *Salmo salar* | SsaTRZ2 | tRNase Z^L^ | C0H8T6 | Uniprot | 875 |
| *Spermophilus tridecemlineatus* | StrTRZ1 | tRNase Z^S^ | ENSSTOP00000009507 | Ensembl | 366 |
| *Spermophilus tridecemlineatus* | StrTRZ2 | tRNase Z^L^ | ENSSTOP00000005982 | Ensembl | ? |
| *Sorex araneus* | SarTRZ1 | tRNase Z^S^ | ENSSARP00000004681 | Ensembl | 363 |
| *Sorex araneus* | SarTRZ2 | tRNase Z^L^ | ENSSARP00000011868 | Ensembl | ? |
| *Strongylocentrotus purpuratus* | SpuTRZ1 | tRNase Z^S^ | XP_001189573.1 | NCBI | 362 |
| *Strongylocentrotus purpuratus* | SpuTRZ2 | tRNase Z^L^ | XP_783904.2 | NCBI | 931* |
| *Sus scrofa* | SscTRZ1 | tRNase Z^S^ | ENSSSCP00000004877 | Ensembl | ? |
| *Sus scrofa* | SscTRZ2 | tRNase Z^L^ | XP_003132056.2 | NCBI | 817 |
| *Takifugu rubripes* | TruTRZ1 | tRNase Z^S^ | 561259 | JGI | 374* |
| *Takifugu rubripes* | TruTRZ2 | tRNase Z^L^ | 595822 | JGI | 846* |
| *Tarsius syrichta* | TsyTRZ1 | tRNase Z^S^ | ENSTSYP00000005466 | Ensembl | 364 |
| *Tarsius syrichta* | TsyTRZ2 | tRNase Z^L^ | ENSTSYP00000004760 | Ensembl | ? |
| *Tetraodon nigroviridis* | TniTRZ1 | tRNase Z^S^ | CAG10876.1 | Uniprot | 386 |
| *Tetraodon nigroviridis* | TniTRZ2 | tRNase Z^L^ | Q4SAV4 | Uniprot | 832* |
| *Xenopus tropicalis* | XtrTRZ1 | tRNase Z^S^ | 476074 | Metazome | 363 |
| *Xenopus tropicalis* | XtrTRZ2 | tRNase Z^L^ | 157533 | Metazome | 835* |
| **Protostomes** |  |  |  |  |  |
| *Acromyrmex echinatior* | AecTRZ1 | tRNase Z^L^ | EGI66981.1 | NCBI | 810 |
| *Acyrthosiphon pisum* | ApiTRZ1 | tRNase Z^L^ | XP_001946795.2 | NCBI | 804 |
| *Aedes aegypti* | AaeTRZ1 | tRNase Z^L^ | XP_001654456.1 | NCBI | 817* |
| *Anopheles darlingi* | AdaTRZ1 | tRNase Z^L^ | EFR21319.1 | NCBI | 813* |
| *Anopheles gambiae str. PEST* | AgaTRZ1 | tRNase Z^L^ | XP_318827.4 | NCBI | 831* |
| *Ascaris suum* | AsuTRZ1 | tRNase Z^L^ | F1KW26 | Uniprot | 878 |
| *Bombyx mori* | BmoTRZ1 | tRNase Z^L^ | Bmb008309 | Metazome | 889* |
| *Brugia malayi* | BmaTRZ1 | tRNase Z^L^ | XP_001900149.1 | NCBI | 817* |
| *Caenorhabditis briggsae* | CbrTRZ1 | tRNase Z^L^ | XP_002634173.1 | NCBI | 849 |
| *Caenorhabditis elegans* | CelTRZ1 | tRNase Z^L^ | NP_001023109.1 | NCBI | 833 |
| *Caenorhabditis remanei* | CreTRZ1 | tRNase Z^L^ | XP_003108315.1 | NCBI | 831 |
| *Camponotus floridanus* | CflTRZ1 | tRNase Z^L^ | EFN69529.1 | NCBI | 769 |
| *Capitella teleta* | CteTRZ1 | tRNase Z^L^-Like | 218896 | JGI | 738 |
| *Capitella teleta* | CteTRZ2 | tRNase Z^L^ | 228344 | JGI | 758 |
| *Culex pipiens* | CpiTRZ1 | tRNase Z^L^ | CpipJ_CPIJ007046.3 | Broad | 784 |
| *Culex quinquefasciatus* | CquTRZ1 | tRNase Z^L^ | XP_001848575.1 | NCBI | 784 |
| *Daphnia pulex* | DpuTRZ1 | tRNase Z^L^ | EFX83742.1 | NCBI | 731* |
| *Drosophila ananassae* | DanTRZ1 | tRNase Z^L^ | XP_001959270.1 | NCBI | 768 |
| *Drosophila erecta* | DerTRZ1 | tRNase Z^L^ | XP_001969068.1 | NCBI | 766* |
| *Drosophila grimshawi* | DgrTRZ1 | tRNase Z^L^ | XP_001987185.1 | NCBI | 791* |
| *Drosophila melanogaster* | DmeTRZ1 | tRNase Z^L^ | NP_724916.1 | NCBI | 766 |
| *Drosophila mojavensis* | DmoTRZ1 | tRNase Z^L^ | XP_002004950.1 | NCBI | 769* |
| *Drosophila sechellia* | DseTRZ1 | tRNase Z^L^ | XP_002033225.1 | NCBI | 766* |
| *Drosophila simulans* | DsiTRZ1 | tRNase Z^L^ | XP_002080889.1 | NCBI | 766* |
| *Drosophila virilis* | DviTRZ1 | tRNase Z^L^ | XP_002050528.1 | NCBI | 784* |
| *Drosophila willistoni* | DwiTRZ1 | tRNase Z^L^ | XP_002068882.1 | NCBI | 772* |
| *Drosophila yakuba* | DyaTRZ1 | tRNase Z^L^ | XP_002089926.1 | NCBI | 766* |
| *Loa loa* | LloTRZ1 | tRNase Z^L^ | LOAG_01193.3 | BROAD | 825* |
| *Lottia gigantea* | LgiTRZ1 | tRNase Z^S^ | 218281 | JGI | 356 |
| *Lottia gigantea* | LgiTRZ2 | tRNase Z^L^ | 156489 | JGI | 738* |
| *Lottia gigantea* | LgiTRZ3 | tRNase Z^L^ | 230203 | JGI | 780 |
| *Heliconius melpomene* | HmeTRZ1 | tRNase Z^L^ | [D0AB78](http://www.uniprot.org/uniprot/D0AB78) | Uniprot | 928 |
| *Helobdella robusta* | HroTRZ1 | tRNase Z^L^ | 189299 | JGI | 925* |
| *Nasonia vitripennis* | NviTRZ1 | tRNase Z^L^ | XP_001604122.1 | NCBI | 849* |
| *Pristionchus pacificus* | PpaTRZ1 | tRNase Z^L^ | PP32102 | WORM | 877* |
| *Sea hare* | ShaTRZ1 | tRNase Z^S^ | GL007682.1 | NCBI | 368 |
| *Sea hare* | ShaTRZ2 | tRNase Z^L^ | GL007017.1 | NCBI | 822* |
| *Schistosoma mansoni* | SmaTRZ1 | tRNase Z^S^ | XP_002578028.1 | NCBI | 418 |
| *Schistosoma mansoni* | SmaTRZ2 | tRNase Z^L^ | XP_002574556.1 | NCBI | 839 |
| *Schistosoma japonicum* | SjaTrz1 | tRNase Z^S^ | AAW27216 | NCBI | 415 |
| *Schistosoma japonicum* | SjaTrz2 | tRNase Z^L^ | tblastn | NCBI | ? |
| *Tribolium castaneum* | TcaTRZ1 | tRNase Z^L^ | XP_968692.2 | NCBI | 827 |
| *Trichinella spiralis* | TspTRZ1 | tRNase Z^L^ | EFV60381.1 | NCBI | 775* |
| *Wuchereria bancrofti* | WbaTRZ1 | tRNase Z^L^ | WUBG_08362.1 | BROAD | 822* |
| **Basal metazoans** |  |  |  |  |  |
| *Amphimedon queenslandica* | AquTZ1 | tRNase Z^S^ | Aqu1.217211 | JGI | 373* |
| *Amphimedon queenslandica* | AquTZ2 | tRNase Z^L^ | XP_003385508.1 | NCBI | 712* |
| *Nematostella vectensis* | NveTRZ1 | tRNase Z^S^ | 186754 | JGI | 342 |
| *Nematostella vectensis* | NveTRZ2 | tRNase Z^L^ | 11980 | JGI | 827* |
| *Nematostella vectensis* | NveTRZ3 | tRNase Z^L^ | 108544 | JGI | 769* |
| *Hydra magnipapillata* | HmaTRZ1 | tRNase Z^S^ | Hma2.228140 | JGI | 359 |
| *Hydra magnipapillata* | HmaTRZ2 | tRNase Z^L^ | Hma2.206546 | JGI | 723* |
| *Trichoplax reptans* | TreTRZ1 | tRNase Z^L^ | B3RMG1 | Uniprot | 728* |
| **Protozoans** |  |  |  |  |  |
| *Monosiga brevicollis* | MbrTRZ1 | tRNase Z^S^ | 25108 | JGI | 380* |
| *Monosiga brevicollis* | MbrTRZ2 | tRNase Z^L^ | 23750 | JGI | 812* |

^#^The number of amino acids in metazoan tRNase Z proteins

*Indicates that mispredicted sequences obtained from the databases have been corrected.

“?” indicates that the protein sequence cannot be accurately predicted due to gaps in the nucleotide sequences.
