# Supplementary material for: Identification and Sequence Analysis of Metazoan tRNA 3′-End Processing Enzymes tRNase Zs
Source: PLoS One. 2012 Sep 4;7(9):e44264. doi: 10.1371/journal.pone.0044264 (PMC3433465; doi:10.1371/journal.pone.0044264)
Supplement: Table S2 — Prediction of the number of introns in metazoan tRNase Z genes. (DOC) [file pone.0044264.s006.doc]

Table S2: Prediction of the number of introns in metazoan tRNase Z genes

| Species | Gene name | Form | Intron number | Gene sizes (bp) |
| --- | --- | --- | --- | --- |
| Mammalians |  |  |  |  |
| *B. taurus* | *BtaTRZ1* | *tRNase ZS* | 2 | 8,439 |
| *B. taurus* | *BtaTRZ2* | *tRNase ZL* | 23 | 15,868 |
| *C. jacchus* | *CjaTRZ1* | *tRNase ZS* | 2 | 8,619 |
| *C. jacchus* | *CjaTRZ2* | *tRNase ZL* | 23 | 23,302 |
| *C. familiaris* | *CfaTRZ1* | *tRNase ZS* | 2 | 8,704 |
| *C. familiaris* | *CfaTRZ2* | *tRNase ZL* | 23 | 19,860 |
| *C. porcellus* | *CpoTRZ1* | *tRNase ZS* | 2 | 6,947 |
| *C. porcellus* | *CpoTRZ2* | *tRNase ZL* | 23 | 20,624 |
| *E. caballus* | *EcaTRZ1* | *tRNase ZS* | 2 | 8,820 |
| *E. caballus* | *EcaTRZ2* | *tRNase ZL* | 23 | 21,220 |
| *H. sapiens* | *HsaTRZ1* | *tRNase ZS* | 2 | 12,681 |
| *H. sapiens* | *HsaTRZ2* | *tRNase ZL* | 23 | 25,130 |
| *L. africana* | *LafTRZ1* | *tRNase ZS* | 2 | 11,163 |
| *L. africana* | *LafTRZ2* | *tRNase ZL* | 23 | 30,392 |
| *M. musculus* | *MmuTRZ1* | *tRNase ZS* | 2 | 8,490 |
| *M. musculus* | *MmuTRZ2* | *tRNase ZL* | 23 | 22,125 |
| *O. cuniculus* | *OcuTRZ1* | *tRNase ZS* | 2 | 10,736 |
| *O. cuniculus* | *OcuTRZ2* | *tRNase ZL* | 23 | 21,070 |
| *P. troglodytes* | *PtrTRZ1* | *tRNase ZS* | 2 | 13,000 |
| *P. troglodytes* | *PtrTRZ2* | *tRNase ZL* | 23 | 25,029 |
| *R. norvegicus* | *RnoTRZ1* | *tRNase ZS* | 2 | 8,267 |
| *R. norvegicus* | *RnoTRZ2* | *tRNase ZL* | 23 | 22,373 |
| *S. scrofa* | *SscTRZ2* | *tRNase ZL* | 23 | 30,717 |
| *T. syrichta* | *TsyTRZ1* | *tRNase ZS* | 2 | 5,508 |
| Non-mammalian vertebrates |  |  |  |  |
| *A. carolinensis* | *AcaTRZ1* | *tRNase ZS* | 2 | 4,663 |
| *A. carolinensis* | *AcaTRZ2* | *tRNase ZL* | 22 | 30,431 |
| *C. intestinalis* | *CinTRZ1* | *tRNase ZS* | 0 | 1,152 |
| *C. intestinalis* | *CinTRZ2* | *tRNase ZL* | 0 | 2,184 |
| *O. latipes* | *OlaTRZ1* | *tRNase ZS* | 4 | 2,838 |
| *O. latipes* | *OlaTRZ2* | *tRNase ZL* | 23 | 8,733 |
| *T. nigroviridis* | *TniTRZ1* | *tRNase ZS* | 3 | 1,633 |
| *T. nigroviridis* | *TniTRZ2* | *tRNase ZL* | 22 | 4,673 |
| *T. rubripes* | *TruTRZ1* | *tRNase ZS* | 4 | 1,540 |
| *T. rubripes* | *TruTRZ2* | *tRNase ZL* | 22 | 4,889 |
| Insects |  |  |  |  |
| *A. echinatior* | *AecTRZ1* | *tRNase ZL* | 5 | 3,762 |
| *A. pisum* | *ApiTRZ1* | *tRNase ZL* | 13 | 7,737 |
| *A. aegypti* | *AaeTRZ1* | *tRNase ZL* | 4 | 13,598 |
| *A. gambiae* | *AgaTRZ1* | *tRNase ZL* | 5 | 2,848 |
| *D. ananassae* | *DanTRZ1* | *tRNase ZL* | 1 | 2,379 |
| *D. erecta* | *DerTRZ1* | *tRNase ZL* | 1 | 2,357 |
| *D. grimshawi* | *DgrTRZ1* | *tRNase ZL* | 1 | 2,558 |
| *D. melanogaster* | *DmeTRZ1* | *tRNase ZL* | 1 | 2,430 |
| *D. sechellia* | *DseTRZ1* | *tRNase ZL* | 1 | 2,358 |
| *D. simulans* | *DsiTRZ1* | *tRNase ZL* | 1 | 2,359 |
| *D. virilis* | *DviTRZ1* | *tRNase ZL* | 1 | 3,181 |
| *D. willistoni* | *DwiTRZ1* | *tRNase ZL* | 1 | 2,341 |
| *D. yakuba* | *DyaTRZ1* | *tRNase ZL* | 1 | 2,357 |
| *H. melpomene* | *HmeTRZ1* | *tRNase ZL* | 14 | 11,845 |
| *N. vitripennis* | *NviTRZ1* | *tRNase ZL* | 6 | 3,495 |
| *T. castaneum* | *TcaTRZ1* | *tRNase ZL* | 8 | 3,349 |
| Nematodes |  |  |  |  |
| *B. malayi* | *BmaTRZ1* | *tRNase ZL* | 14 | 5,298 |
| *C. briggsae* | *CbrTRZ1* | *tRNase ZL* | 6 | 3,035 |
| *C. elegans* | *CelTRZ1* | *tRNase ZL* | 6 | 4,595 |
| *C. remanei* | *CreTRZ1* | *tRNase ZL* | 6 | 2,888 |
| *L. loa* | *LloTRZ1* | *tRNase ZL* | 15 | 5,939 |
