# Supplementary material for: Identification and Sequence Analysis of Metazoan tRNA 3′-End Processing Enzymes tRNase Zs
Source: PLoS One. 2012 Sep 4;7(9):e44264. doi: 10.1371/journal.pone.0044264 (PMC3433465; doi:10.1371/journal.pone.0044264)
Supplement: Table S3 — Subcellular localization prediction of metazoan tRNase ZLs. The putative NLSs were predicted using PSORT (http://psort.hgc.jp/form.html), while the putative MTSs were predicted using MITOPROT (http://ihg2.helmholtz-muenchen.de/ihg/mitoprot.html). Nuc: stand for the nucleus; Mito: mitochondria. N indicates nuclear localization and M denotes mitochondrial localization. “-”, the localization or targeting sequence could not be predicted. The numbers refer to amino acid positions starting from the N-terminus. (DOC) [file pone.0044264.s007.doc]

**Table S3: Prediction of the nuclear localization signals and the mitochondrial targeting sequences in metazoan tRNase Z^L^**s

| Protein name | Nuc | Mito | Predicted nuclear localization signal | Predicted mitochondrial targeting sequence |
| --- | --- | --- | --- | --- |
| AaeTRZ1 | N | M | ^438^RPKK^441^, ^241^KRHEETVMAYICKLKLR^257^ | ^1^MYKLLTSLRPNVVGSASVIRFYSSNLKPQRK^31^ |
| AcaTRZ2 | N | M | ^24^RRPR^27^ | ^1^MWRRLLGSVAGAG^13^ |
| AecTRZ1 | - | M | - | - |
| AdaTRZ1 | N | M | ^244^KREEESVMAYICKLKPR^260^ | ^1^MYSVFRLVNPYVVSHYSISRRWY^23^ |
| AgaTRZ1 | N | M | ^248^KREESSVMAYICKLKPR^264^ | ^1^MYTISRLVNSNVVRSKVPVRW^21^ |
| ApiTRZ1 | N | M | ^216^KRKR^219^ | - |
| AquTZ2 | - | M | - | ^1^MIPLIRFSLWRR^12^ |
| AsuTRZ1 | N | M | ^868^RKRK^872^ | ^1^MSLCCSSFVLLQGSCFGKTIVALLRPTKLLRSLSSRSRYS^40^ |
| BflTRZ2 | N | - | ^10^KKRK^13^, ^11^KRKK^14^, ^219^KRRK^222^, ^239^KPRK^242^  ^689^KRKR^292^, ^690^RKRH^693^, ^773^KRHK^776^, ^774^RHKR^777^ | - |
| BmaTRZ1 | N | - | ^799^KKKP8^02^, ^787^RKFVDTLGCTAQKKKPS^803^ | - |
| BmoTRZ1 | N | M | ^174^KKPK^177^ | - |
| BtaTRZ2 | N | M | ^24^RRPR^27^, ^421^RPRR^424^, ^796^KRAPTEHPLSPQSKKAR^812^ | ^1^MWALRSLLGLRCAAGRTMSQGPARRP^26^ |
| CbrTRZ1 | N | M | ^647^KRPK^650^, ^741^RKRH^744^, ^645^PGKRPKL^651^ | ^1^MKICLNLIFSCFEKTSVQIVISQLYSILLFRIPRN^35^ |
| CelTRZ1 | N | M | ^636^KRPR^639^, ^730^RKRH^733^, ^634^PGKRPRL^640^ | ^1^MLGAIARKTVENRILVSRHLISSTSCL^27^ |
| CfaTRZ2 | N | M | ^420^RPRR^423^, ^803^KRALVEQPRSPQSKKVR^819^ | ^1^MWLLRSLLLLRSAAGRAMSQGPARRQ^27^ |
| CflTRZ1 | - | M | - | ^1^MPRSSTHVLNMQLTREKLKKKSVKYLGNVNVSLQILGSGARG^42^ |
| CinTRZ2 | N | - | ^497^PRKR^500^ | - |
| CjaTRZ2 | N | M | ^428^RPRR^431^ | ^1^MWALCCLLRFAARRTMSQERTLSQGSARRQ^31^ |
| CreTRZ1 | N | M | ^629^KRPR^632^, ^723^RKRH^726^, ^627^PGKRPRL^633^ | ^1^MRRAVSRIVENRTLLTRFFSSTSPLF^26^ |
| CpoTRZ2 | N | M | ^424^RPRR427, ^807^KRALAEQPQSPQSKKAR^823^ | ^1^MWALRSVLRSVAGRTMSQGPARRQ^25^ |
| CsaTRZ2 | - | M | - | ^1^MFCCLKTFHFLKNFNILNHSCYTCWNNGGINLVNGVRNSSINFRRNY^47^ |
| CteTRZ1 | N | - | ^706^PRKK^709^, ^707^RKKH^710^, ^708^KKHK^711^  ^692^RKMSSWMEREANREPRK^708^ | - |
| DanTRZ1 | N | M | ^407^RPKK^410^ ^761^RKRK^764^ | ^1^MFVVKSRCWPLYGTIRTFKMSSSVA^25^ |
| DerTRZ1 | N | M | ^405^RPRK^408^, ^759^RKRK^762^, ^371^KKTKLDETDSEDKRNSK^387^ | ^1^MYLVKSAGSPIYRTLRTLSTRNLMA^25^ |
| DgrTRZ1 | N | M | ^428^RPKK^431^, ^784^RKRK^787^ | ^1^MWLPLLKLQRIALHRTLRKLTKHSIRM^27^ |
| DmeTRZ1 | N | M | ^405^RPRK^408^, ^759^RKRK^762^ | ^1^MYLVKSARSPIYRTLRTLTTSNLMA^25^ |
| DmoTRZ1 | N | M | ^408^RPKK^411^ | ^1^MLKLQRIVPHGPLRSLTNHCIRM^23^ |
| DreTRZ2 | N | - | ^877^KREQEDHNQENANKRLK^893^ | - |
| DseTRZ1 | N | M | ^405^RPRK^408^, ^759^RKRK^762^ | ^1^MYLVKSAGSPIYRTLRTLTTSNLMS^25^ |
| DsiTRZ1 | N | M | ^405^RPRK^408^, ^759^RKRK^762^ | ^1^MYLVKSAGSPIYRTLRTLTTSNLMS^25^ |
| DviTRZ1 | N | M | ^423^RPKK^426^, ^777^RKRK^780^ | ^1^MWLAMLKLQRIAPHGKLRTLTENCIRM^27^ |
| DwiTRZ1 | N | M | ^411^RPRK^414^, ^765^RKRK^768^ | ^1^MIFLKFPRPLLYGQHLRRRQQIHRLINMSSSVV^33^ |
| DyaTRZ1 | N | M | ^405^RPRK^408^, ^759^RKRK^762^ | ^1^MYLVKSAGSPILRTLRTLTTCNLMS^25^ |
| EcaTRZ2 | N | M | ^419^RPRR^422^, ^802^KRAHAEEPQNPQSKKVR^818^ | ^1^MWALRSLLLRAAAGRA^17^ |
| GgaTRZ2 | N | M | ^860^KRPK^863^ | ^1^MCRLAWTLRRGLAWGAGTV^9^ |
| GacTRZ2 | N | M | ^28^RRPK^31^, ^28^RRPKDVPRHVWARERRR^44^  ^820^KRKQDKNHQEVSNKKLK^836^  ^821^RKQDKNHQEVSNKKLKK^837^ | ^1^MNAVREKLGSIPLLFIKGRSLSAGPPAVPSCYFPVPRTVLQFFRT^45^ |
| HmaTRZ2 | N | - | ^379^RKRK^382^ | - |
| HmeTRZ1 | N | M | ^511^RPKK^514^, ^273^KKHNHCTVAYICILKKR^289^ | - |
| HroTRZ1 | - | - |  | - |
| HsaTRZ2 | N | M | ^31^RPRK^34^, ^428^RPRR^431^ | ^1^MWALCSLLRSAAGRTMSQGRTISQAPARRE^30^ |
| LafTRZ2 | N | M | ^422^RPRR^425^, ^801^KRVPRDEPPSPQSKKAR^817^ | ^1^MWALRSLLRAAAGGTMSQGPARRQ^24^ |
| LgiTRZ2 | N | - | ^8^PKRK^11^, ^181^KKPR^184^ | - |
| LgiTRZ3 | N | M | ^213^KKPK^216^, ^200^RKKEKTDEAAFASKKPK^216^  ^201^KKEKTDEAAFASKKPKL^217^ | ^1^MGLKLGTSGESFVLCKFHKIQTRTY^25^ |
| LgoTRZ2 | N | M | ^31^RPRK^34^, ^428^RPRR^431^ | ^1^MWALCSLLRSAAGRTMSQGRTISQAPARRE^30^ |
| LloTRZ1 | N | - | ^795^RRFVDTLGGTVQKKKAS^811^ | - |
| MbrTRZ2 | N | M | ^396^KRRTRQDRGRESKRAKT^412^ | ^1^MWGRRLVWHSCGLMDAAVQRRAYRM^25^ |
| MmuTRZ2 | N | M | ^22^RRPR^25^, ^424^RPKR^427^  ^806^KRAHTDEPHSPQSKKVR^822^ | ^1^MWALCSLLRSAAGRTMSQGRTISQGSARRQ^30^ |
| NveTRZ2 | N | M | ^781^RRALYGNKPQSSIKKQR^797^ | ^1^MIVGFLQCRTPCFRGFTFKSCTG^23^ |
| NveTRZ3 | - | M | - | ^1^MLSCRVIQSYLTKLSSVVQKCLLGRK^26^ |
| NviTRZ1 | - | M | - | ^1^MIFSKFYCLTTFHRRTTIIIRQY^23^ |
| OcuTRZ2 | N | M | ^424^RPRR^427^, ^807^KRAHTGEPHSPQSKKVR^823^ | ^1^MWTLRSLWRSVAGRTMSQGPARRQ^24^ |
| OlaTRZ2 | - | M | - | ^1^MNAVHHVKVRFFTSLLRFGQSLSVGPSIFSPPSLRRTVLWGFRT^44^ |
| PpaTRZ1 | N | - | ^781^RKKH^784^ | - |
| PtrTRZ2 | N | M | ^31^RPRK^34^, ^428^RPRR^431^ | ^1^MWALCSLLRSAAGRTMSQGRTISQAPARRE^30^ |
| RnoTRZ2 | N | M | ^22^RRPR^25^, ^427^RPKR^430^, ^809^KRAHSEEPHSPQSKKVR^825^ | ^1^MWALRSLLRPLGLRTMSQGSARRP^24^ |
| ShaTRZ2 | - | M | - | ^1^MGRRKGKAGKGAPHHKNSGNVEAAANAR^29^ |
| SkoTRZ2 | - | M | - | ^1^MAVASISRITKIIIRNRHLNCVRSVFTRRNRR^32^ |
| SmaTRZ2 | N | M | ^20^KHKR^23^, ^174^KRRK^177^ | ^1^MLRPSLCYVRLITTPKRFL^19^ |
| SpuTRZ2 | N | M | ^352^KKRK^358^, ^825^KKKR^828^, ^826^KKRH^829^  ^911^KKAERKVINKQMTKKKM^927^ | ^1^MYLSSFSVSTARCCKNVIIRNTLSKNGIACPSFTFSCKHSSTSSTSLSLGLLSRSRLKIVTENTNFLSICNVRRISSGKNWL^82^ |
| SsaTRZ2 | N | M | ^61^KKPR^64^, ^857^KREPEEPTQDAGSKRLK^873^ | ^1^MFSMTKVSLPCKSIAWLFVRHTSTGRLQSFACSEHRFPRTIFLFLRT^47^ |
| SscTRZ2 | N | M | ^422^RPRR^425^, ^799^KRAHAERPPSPQSKKVR^815^ | ^1^MWALRSLLGLRSVAGRTMSQGPARRQ^26^ |
| TcaTRZ1 | N | M | ^459^RPKK^462^, ^501^KRRH^504^, ^417^PQPKKIK^423^ | ^1^MSSRVSFIQARRY^13^ |
| TniTRZ2 | N | M | ^47^PRKARHK^53^ | ^1^MSPGCLRLFLGAGRLPAARCRL^22^ |
| TadTRZ1 | - | M | - | ^1^MIFKQLSRKLSRISKSM^17^ |
| TruTRZ2 | N | M | ^53^KKEPLRLIKSKESRSKR^69^ | ^1^MIRGHLKLLTLPFTGVLSAARCHCPPHRTVFQLFRT^36^ |
| TspTRZ1 | - | M | - | ^1^MIYFQQLIFALTKKRCSIWPSSSSVWLNIRLLSTMPPSP^39^ |
| WbaTRZ1 | N | - | ^792^RRFVDTLGCTAQKKKAS^808^ | - |
| XtrTRZ2 | N | M | ^32^RKDKPPKDTLRHIKRRE^48^  ^817^KRDLEGASHTMGNKRLK^833^ | - |
