# Supplementary material for: Identification and Sequence Analysis of Metazoan tRNA 3′-End Processing Enzymes tRNase Zs
Source: PLoS One. 2012 Sep 4;7(9):e44264. doi: 10.1371/journal.pone.0044264 (PMC3433465; doi:10.1371/journal.pone.0044264)
Supplement: Table S4 — Percentage amino acid identity among tRNase ZLs from selected metazoans. The pairwise percent identity scores were generated with Clustal W [52]. tRNase ZLs are from H. sapiens (Hsa), C. familiaris (Cfa), M. musculus (Mmu), R. norvegicus (Rno), O. cuniculus (Ocu), S. scrofa (Ssc), A. carolinensis (Aca), X. tropicalis (Xtr), D. rerio (Dre), G. aculeatus (Gac), C. intestinalis (Cin), C. savignyi (Csa), B. mori (Bmo), D. melanogaster (Dme), C. elegans (Cel), H. robusta (Hro), S. mansoni (Sma), T. adhaerens (Tad), M. brevicollis, (Mbr), A. thaliana (Ath), and S. pome (Spo). (DOC) [file pone.0044264.s008.doc]

Table S4：Percentage amino acid identity among tRNase Z^L^s from selected metazoans

|  | Cfa | Mmu | Rno | Ocu | Ssc | Aca | Xtr | Dre | Gac | Cin | Csa | Bmo | Dme | Cel | Hro | Sma | Tad | Mbr | Ath | Spo |
| --- | --- | --- | --- | --- | --- | --- | --- | --- | --- | --- | --- | --- | --- | --- | --- | --- | --- | --- | --- | --- |
| Hsa | 82 | 82 | 81 | 82 | 79 | 65 | 60 | 52 | 54 | 19 | 20 | 29 | 32 | 24 | 26 | 26 | 29 | 22 | 24 | 24 |
| Cfa |  | 82 | 82 | 82 | 82 | 65 | 60 | 52 | 55 | 20 | 20 | 29 | 31 | 23 | 25 | 26 | 29 | 25 | 24 | 23 |
| Mmu |  |  | 95 | 82 | 79 | 66 | 60 | 52 | 54 | 20 | 19 | 29 | 31 | 23 | 25 | 25 | 29 | 23 | 25 | 25 |
| Rno |  |  |  | 82 | 79 | 66 | 59 | 52 | 54 | 20 | 18 | 29 | 31 | 24 | 24 | 25 | 29 | 22 | 25 | 25 |
| Ocu |  |  |  |  | 80 | 66 | 59 | 52 | 55 | 20 | 19 | 30 | 31 | 23 | 26 | 25 | 29 | 23 | 25 | 24 |
| Ssc |  |  |  |  |  | 65 | 60 | 51 | 55 | 19 | 21 | 30 | 31 | 23 | 27 | 25 | 30 | 23 | 25 | 25 |
| Aca |  |  |  |  |  |  | 60 | 52 | 53 | 19 | 18 | 29 | 31 | 22 | 25 | 24 | 28 | 22 | 23 | 24 |
| Xtr |  |  |  |  |  |  |  | 54 | 55 | 19 | 20 | 28 | 31 | 24 | 25 | 24 | 28 | 24 | 27 | 25 |
| Dre |  |  |  |  |  |  |  |  | 58 | 17 | 20 | 28 | 28 | 24 | 24 | 24 | 27 | 21 | 25 | 23 |
| Gac |  |  |  |  |  |  |  |  |  | 16 | 19 | 29 | 29 | 24 | 24 | 23 | 27 | 21 | 26 | 24 |
| Cin |  |  |  |  |  |  |  |  |  |  | 35 | 17 | 19 | 17 | 18 | 16 | 20 | 18 | 17 | 17 |
| Csa |  |  |  |  |  |  |  |  |  |  |  | 16 | 17 | 18 | 16 | 16 | 22 | 15 | 18 | 16 |
| Bmo |  |  |  |  |  |  |  |  |  |  |  |  | 36 | 21 | 24 | 23 | 26 | 21 | 22 | 22 |
| Dme |  |  |  |  |  |  |  |  |  |  |  |  |  | 23 | 25 | 25 | 27 | 23 | 24 | 23 |
| Cel |  |  |  |  |  |  |  |  |  |  |  |  |  |  | 21 | 20 | 20 | 20 | 22 | 18 |
| Hro |  |  |  |  |  |  |  |  |  |  |  |  |  |  |  | 23 | 23 | 20 | 21 | 19 |
| Sma |  |  |  |  |  |  |  |  |  |  |  |  |  |  |  |  | 22 | 20 | 20 | 20 |
| Tad |  |  |  |  |  |  |  |  |  |  |  |  |  |  |  |  |  | 23 | 24 | 25 |
| Mbr |  |  |  |  |  |  |  |  |  |  |  |  |  |  |  |  |  |  | 22 | 20 |
| Ath |  |  |  |  |  |  |  |  |  |  |  |  |  |  |  |  |  |  |  | 23 |
