# Supplementary material for: Identification and Sequence Analysis of Metazoan tRNA 3′-End Processing Enzymes tRNase Zs
Source: PLoS One. 2012 Sep 4;7(9):e44264. doi: 10.1371/journal.pone.0044264 (PMC3433465; doi:10.1371/journal.pone.0044264)
Supplement: Table S5 — Percentage amino acid identity among tRNase ZSs from selected metazoans. The pairwise percent identity scores were generated with Clustal W [52]. H. sapiens, Hsa; M. musculus, Mmu; R. norvegicus, Rno; O. cuniculus, Ocu; S. araneus, Sar; T. syrichta, Tsy; A. carolinensis, Aca; X. tropicalis, Xtr; D. rerio, Dre; G. aculeatus, Gac; C. intestinalis, Cin; C. savignyi, Csa; B. floridae, Bfl, L. gigantean, Lgi; S. mansoni, Sma; A. queenslandica, Aqu; N. vectensis, Nve; M. brevicollis, Mbr; B. subtilis, Bsu; E. coli, Eco. (DOC) [file pone.0044264.s009.doc]

Table S5：Percentage amino acid identity among tRNase Z^S^s from selected metazoans

|  | Mmu | Rno | Ocu | Sar | Tsy | Aca | Xtr | Dre | Gac | Cin | Csa | Blf | Lgi | Sma | Aqu | Nve | Mbr | Bsu | Eco |
| --- | --- | --- | --- | --- | --- | --- | --- | --- | --- | --- | --- | --- | --- | --- | --- | --- | --- | --- | --- |
| Hsa | 92 | 92 | 90 | 92 | 94 | 72 | 70 | 57 | 59 | 41 | 40 | 49 | 49 | 35 | 41 | 45 | 28 | 34 | 33 |
| Mmu |  | 97 | 86 | 90 | 89 | 71 | 68 | 57 | 58 | 41 | 41 | 47 | 47 | 34 | 40 | 46 | 28 | 34 | 32 |
| Rno |  |  | 88 | 90 | 89 | 70 | 68 | 56 | 58 | 41 | 41 | 47 | 47 | 35 | 40 | 45 | 28 | 34 | 32 |
| Ocu |  |  |  | 86 | 87 | 70 | 68 | 57 | 58 | 41 | 41 | 48 | 46 | 34 | 40 | 45 | 28 | 33 | 34 |
| Sar |  |  |  |  | 89 | 71 | 67 | 57 | 52 | 42 | 41 | 48 | 48 | 35 | 41 | 45 | 28 | 34 | 33 |
| Tsy |  |  |  |  |  | 70 | 68 | 58 | 59 | 41 | 41 | 48 | 48 | 34 | 43 | 46 | 27 | 34 | 34 |
| Aca |  |  |  |  |  |  | 68 | 59 | 59 | 41 | 41 | 47 | 49 | 34 | 43 | 43 | 28 | 32 | 33 |
| Xtr |  |  |  |  |  |  |  | 57 | 59 | 42 | 42 | 49 | 48 | 34 | 42 | 45 | 29 | 32 | 34 |
| Dre |  |  |  |  |  |  |  |  | 72 | 43 | 43 | 49 | 49 | 33 | 44 | 46 | 26 | 33 | 32 |
| Gac |  |  |  |  |  |  |  |  |  | 41 | 41 | 47 | 46 | 34 | 44 | 46 | 27 | 34 | 33 |
| Cin |  |  |  |  |  |  |  |  |  |  | 77 | 44 | 42 | 33 | 41 | 39 | 25 | 29 | 31 |
| Csa |  |  |  |  |  |  |  |  |  |  |  | 46 | 42 | 34 | 42 | 42 | 26 | 30 | 32 |
| Bfl |  |  |  |  |  |  |  |  |  |  |  |  | 53 | 35 | 47 | 50 | 28 | 35 | 33 |
| Lgi |  |  |  |  |  |  |  |  |  |  |  |  |  | 35 | 47 | 48 | 26 | 35 | 34 |
| Sma |  |  |  |  |  |  |  |  |  |  |  |  |  |  | 32 | 33 | 24 | 24 | 26 |
| Aqu |  |  |  |  |  |  |  |  |  |  |  |  |  |  |  | 48 | 27 | 33 | 33 |
| Nve |  |  |  |  |  |  |  |  |  |  |  |  |  |  |  |  | 28 | 38 | 36 |
| Mbr |  |  |  |  |  |  |  |  |  |  |  |  |  |  |  |  |  | 30 | 31 |
| Bsu |  |  |  |  |  |  |  |  |  |  |  |  |  |  |  |  |  |  | 48 |
